# DNA transposons and the role of recombination in mutation accumulation in *Daphnia pulex*

**DOI:** 10.1186/gb-2010-11-4-r46

**Published:** 2010-04-30

**Authors:** Sarah Schaack, Eunjin Choi, Michael Lynch, Ellen J Pritham

**Affiliations:** 1 Department of Biology, University of Texas-Arlington, 501 S. Nedderman Drive, Arlington, TX 76019, USA; 2 Department of Biology, Indiana University, 1001 E. Third St, Bloomington, IN 47405, USA

## Abstract

**Background:**

We identify DNA transposons from the completed draft genome sequence of *Daphnia pulex*, a cyclically parthenogenetic, aquatic microcrustacean of the class Branchiopoda. In addition, we experimentally quantify the abundance of six DNA transposon families in mutation-accumulation lines in which sex is either promoted or prohibited in order to better understand the role of recombination in transposon proliferation.

**Results:**

We identified 55 families belonging to 10 of the known superfamilies of DNA transposons in the genome of *D. pulex*. DNA transposons constitute approximately 0.7% of the genome. We characterized each family and, in many cases, identified elements capable of activity in the genome. Based on assays of six putatively active element families in mutation-accumulation lines, we compared DNA transposon abundance in lines where sex was either promoted or prohibited. We find the major difference in abundance in sexuals relative to asexuals in lab-reared lines is explained by independent assortment of heterozygotes in lineages where sex has occurred.

**Conclusions:**

Our examination of the duality of sex as a mechanism for both the spread and elimination of DNA transposons in the genome reveals that independent assortment of chromosomes leads to significant copy loss in lineages undergoing sex. Although this advantage may offset the so-called 'two fold cost of sex' in the short-term, if insertions become homozygous at specific loci due to recombination, the advantage of sex may be decreased over long time periods. Given these results, we discuss the potential effects of sex on the dynamics of DNA transposons in natural populations of *D. pulex*.

## Background

The role of recombination (hereafter used interchangeably with sex) in transposable element (TE) proliferation has been of great interest for nearly three decades [1]; however, the question of whether or not sex leads to a net increase or decrease in TE abundance over time persists. Generally, a switch to asexuality is thought to eliminate the possibility of reconstructing the least-loaded class via recombination, and thus to irreversibly larger mutation loads (that is, Muller's ratchet [2,3]). In the special case of TEs, however, sex can result in an increased rate of both gain and loss, thereby complicating the predictions of the net effects of reproductive strategy over long time periods. This is because, although there are several mechanisms of gain and loss that do not differ between sexuals and asexuals, only sexuals undergo meiosis. Furthermore, the two main components of meiosis (crossover - ectopic and homologous - and independent assortment) both impact the rate at which new copies are propagated or purged from the genome (for example, [4]).

Previous studies have looked at the accumulation of TEs in selection lines, natural populations, or sister taxa in which outcrossing and inbreeding are used as proxies for high and low recombination, respectively [5-8]. Although these studies provide insight into TE behavior under certain circumstances, none allow for a comparison of TE behavior in sexual versus asexual backgrounds without introducing confounding variables (for example, selection, genetic variation, or species differences). Other studies have considered the relationship between local recombination rate and TE abundance in sexually reproducing organisms (for example, [9,10]), but these data do not provide insight into the consequences of a complete switch between sexual versus asexual reproduction. Cyclical parthenogenesis offers an ideal system to address the role of recombination in TE proliferation because sexuals and asexuals can be compared directly and the results can be generalized to help elucidate the maintenance of sex, as well as the repeated evolution of asexuality as a strategy within otherwise sexual clades.

*Daphnia pulex *is an aquatic microcrustacean found mainly in freshwater habitats throughout North America (class Branchiopoda, order Cladocera). Like other closely related taxa in this clade, most *D. pulex *are cyclical parthenogens: a reproductive strategy composed primarily of asexual reproduction with a seasonal switch to sex that produces hardy, diapausing eggs prior to the onset of winter. These meiotically produced eggs are encased in ephippia that hatch in response to seasonal cues, such as changes in day length and temperature. Newly hatched offspring develop and reproduce via asexual reproduction until environmental conditions change the following year. *D. pulex *is the first crustacean and first cyclical parthenogen for which whole genome sequence data are available.

In order to examine TE proliferation in this species, we surveyed the genome of *D. pulex *for DNA transposons (Class 2). Autonomous transposons encode a transposase and mobilize using a cut-and-paste mechanism of replication, which typically involves excision, transposition of a DNA intermediate, and integration into a new site in the genome (subclass 1) [11]. The mechanism of replication for the more recently discovered subclass 2 elements (*Helitrons *and *Mavericks*), however, is not known (see [12] for review). Although, DNA transposons are generally not thought to exhibit replicative gains when mobilized, for members of subclass 1, copy number can increase due to homologue-dependent DNA repair after excision at homozygous loci, which can result in the reconstitution of a TE in the donor location and, therefore, replicative gain. Class 1 elements (copy-and-paste retrotransposons) include a more diverse array of mechanisms of replication but, generally, do not excise, and the successful reintegration of the RNA intermediate typically results in a net increase in TE abundance, regardless of whether the mobilized element is homozygous or heterozygous. These and other differences may impact patterns of TE spread for the two major classes, thus we restrict our survey here to those belonging to Class 2, but including both autonomous and non-autonomous families and representatives of the recently discovered *Helitron *subclass.

Using representatives of several TE superfamilies identified in our survey of the genome, we assayed six families of DNA transposons in mutation-accumulation (MA) lineages of *D. pulex *in which sex was either promoted or prohibited. Based on the factors influencing DNA transposon dynamics in sexuals versus asexuals, we predicted lab-reared lineages undergoing sex would exhibit both higher rates of both DNA transposon gain and loss than their asexual counterparts. We describe the general landscape of DNA transposons in *D. pulex*, survey the relative abundance of each TE family in MA lines with and without sex, and discuss the implications of the patterns observed for the role of DNA transposons in shaping the genomes of species with multiple reproductive strategies over longer time periods.

## Results

### DNA transposons in *D. pulex*

**Figure 1:**
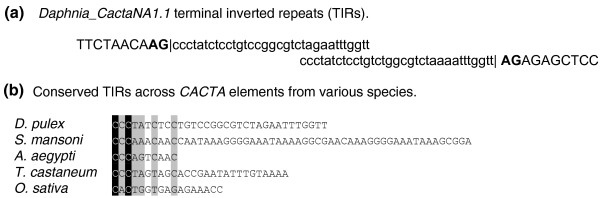
**Classification of *CACTA *DNA transposons in *D. pulex *based on alignments of terminal inverted repeats (TIRs)**. Alignment of **(a) **TIRs for Daphnia_*CACTANA1.1 *elements and **(b) **conserved TIR structure from *CACTA *elements from various taxa including *Daphnia*.

**Figure 2:**
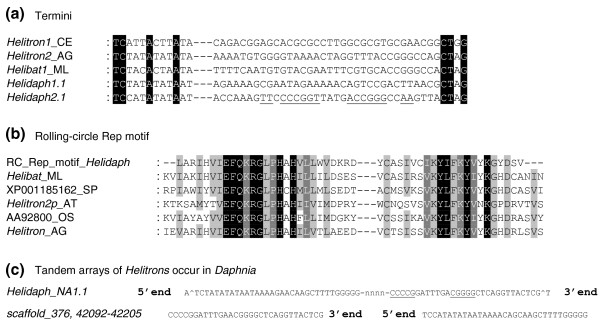
**Classification of *Helitrons *in *D. pulex *based on structural features and conserved coding region**. Alignment of **(a) ***Helitron *termini showing conservation across species, including *HelidaphNA1.1 *and *HelidaphNA2.1*, **(b) **the rolling-circle Rep domain showing conservation across species, including *D. pulex*, and **(c) **5' and 3' ends of *HelidaphNA1.1 *copies found in tandem arrays in the genome.

Using a combination of homology-based and structural search strategies (see Materials and methods), we discovered new elements belonging to nine superfamilies of DNA transposons in *D. pulex*, the first cyclical parthenogen and microcrustacean for which the whole genome sequence is available (Table 1; Table S1 in Additional file 1). In addition to the previously characterized PiggyBac transposon family, *Pokey *[13,14], we found 56 families representing a total of 10 superfamilies in the whole genome sequence (approximately 8× coverage; see Additional file 2 for Supplemental Dataset S1 containing FASTA files of all canonical representatives available and locations on scaffolds available in Table S4). Membership of each complete TE identified to a given superfamily was validated by verifying the presence of the structural characteristic features of that superfamily [12]. Alignments showing homologous regions of one or more representative(s) of each major group found in *D. pulex *with those from various taxa reveal conserved motifs in protein-coding regions (Additional file 3a-j), such as those with predicted catalytic function (for example, *hAT*, *PIF*/*Harbinger*, *Merlin*, *P*, and *Tc1*/*mariner *[15-18]) or polymerase activity (for example, *Maverick *[19]). The *Mutator *superfamily representatives in the *D. pulex *genome all shared high levels of similarity with a recently discovered subgroup called *Phantom *[20]; Additional file 3f). In addition to homologous proteins, superfamily identity was determined by structural motifs such as, in the case of *CACTA *elements, terminal inverted repeats (Figure 1) [21] and, in the case of *Helitrons*, palindromes and the identification of tandem arrays of elements (Figure 2) [22], which is characteristic of this group.

**Table 1:** Estimated copy numbers and total length for families of Class 2 DNA transposons identified in *D. pulex *listed by subclass and superfamily.

Superfamily	Element type	Number of copies (BLASTN)	Number of copies (RM)	Total DNA length
Subclass 1				
*CACTA*	CactaA1.1*^§^	18	26	26,334
	CactaA2.1*	3	4	2,494
	CactaA3.1*^§^	11	11	10,397
	CactaA4.1*^§^	10	12	16,296
	CactaA5.1*^§^	5	7	8,933
	CactaA6.1*	3	6	6,614
	CactaA7.1*^§^	11	10	8,537
	CactaA8.1*	3	7	7,483
	CactaA9.1*^§^	11	19	24,438
	CactaA10.1*	5	7	10,255
				
*hAT*	hATA1.1*^§^	2	3	6,007
	*hATNA1.1*	8	2	711
	hATA2.1*	5	3	3,680
	hATA3.1*^§^	7	7	9,743
	hATA4.1*^§^	5	13	12,565
	hATA5.1*^§^	10	5	8,087
				
*Merlin*	MerlinA1.1*^§^	11	26	36,439
				
*Mutator*	MutatorA1.1*^§^	7	22	21,983
	MutatorA2.1*^§^	24	39	32,291
	MutatorA3.1*^§^	4	28	15,279
	MutatorA4.1*^§^	9	15	18,443
	MutatorA5.1*^§^	13	9	8,898
	MutatorA6.1*^§^	19	20	8,207
	MutatorA7.1*	4	8	8,831
	MutatorA8.1*	9	23	10,058
	MutatorA9.1*^§^	6	5	3,015
	MutatorA10.1*^§^	6	26	22,288
				
*P-element*	PelementA1.1*	6	8	12,256
	PelementA2.1*	20	21	34,335
	PelementA3.1*	1	5	7,053
	PelementA4.1*	5	6	11,047
	PelementA5.1*	3	3	6,727
	PelementA6.1*	1	6	5,300
	*PelementA7.1*	4	2	1,605
	PelementA8.1*	6	2	717
	*PelementNA9.1*^§^	19	17	14,218
				
*PIF*	PIFA1.1*^§^	4	6	5,918
	*PIFA2.1*^§^	4	9	8,033
				
*PiggyBac*/*TTAA*^a^	Pokey*^§^	35	123	271,056
	TTAANA1.1*^§^	130	445	216,417
	TTAANA2.1*^§^	40	117	39,552
				
*Tc1*/*mariner*	AntA1.1*	1	1	1,041
	PogoA1.1*^§^	4	14	34,069
	PogoA2.1*^§^	9	35	37,953
	PogoA3.1*	3	4	7,615
	Tc1A1.1*^§^	2	2	3,519
	Tc1NA1.1*^§^	247	143	63,916
	Tc1NA2.1*^§^	7	18	23,803
				
Sublclass 2				
*Helitron*	HeliDaphA1.1*^§^	58	60	136,160
	HeliDaphA2.1*^§^	42	55	107,599
	*HeliDaphNA1.1*^§^	346	389	159,331
	*HeliDaphNA2.1*^§^	27	69	52,123
				
*Maverick*	MaverickA1.1*	2	1	2,179
	MaverickA2.1*	1	1	1,181
	MaverickA3.1*	2	1	3,380
	MaverickA4.1*	2	2	1,892
				
Total		1,260	1,708	1,466,236

Copy number estimates are based on filtered outputs from BLASTN (e-value < 0.00001 and >20% of the length of the query) and RepeatMasker (RM; >50 bp in length, >70% similarity, and >20% of the length of the query), respectively. Families with asterisks (*) contain ORFs greater than 100 amino acids in length and families with section sign (^§^) have hits in the *D. pulex *genome of >90% over >80% of their length at the nucleotide level, indicating they may be capable of current activity. ^a^Elements flanked by TTAA nucleotides, but with insufficient evidence to be confirmed *PiggyBac *elements, were classified as *TTAA *elements, along with the previously identified *Pokey *element [14], known to exhibit this characteristic flanking sequence.

### Mutation-accumulation experiment

To assess the relative abundance and behavior of DNA transposons in *D. pulex*, representatives from five of the nine recently identified TE superfamilies and the previously identified *PiggyBac *family, *Pokey*, were surveyed in the MA lineages. Families were chosen based on sequence data indicative of potentially recent activity (for example, intact ORFs and between element alignments). Single-copy families or families for which no variation was detected (presence-absence among a subset of MA lines after more than 20 generations) were not assayed. The TE families, referred to here based on their homology to other known DNA transposon families in other species (*Tc1A1.1*, *Tc1NA2.1*, *Helidaph NA1.1*, *Helidaph NA2.1*, *hATA1.1*), as well as *Pokey*, were surveyed across lab-reared lineages using transposon display (TD; see Materials and methods). These lineages had undergone approximately 40 generations of mutation accumulation (see Additional file 4 for the number of generations for each lineage individually) during which they experienced minimal selection and were propagated exclusively via asexual reproduction. Environmental cues were used to induce sexual reproduction (selfing), which, when it occurred, generated sexual sublines that experienced at least one bout of sex but were otherwise treated the same (hereafter treatments referred to as asexuals and sexuals, respectively; see Materials and methods).

**Figure 3:**
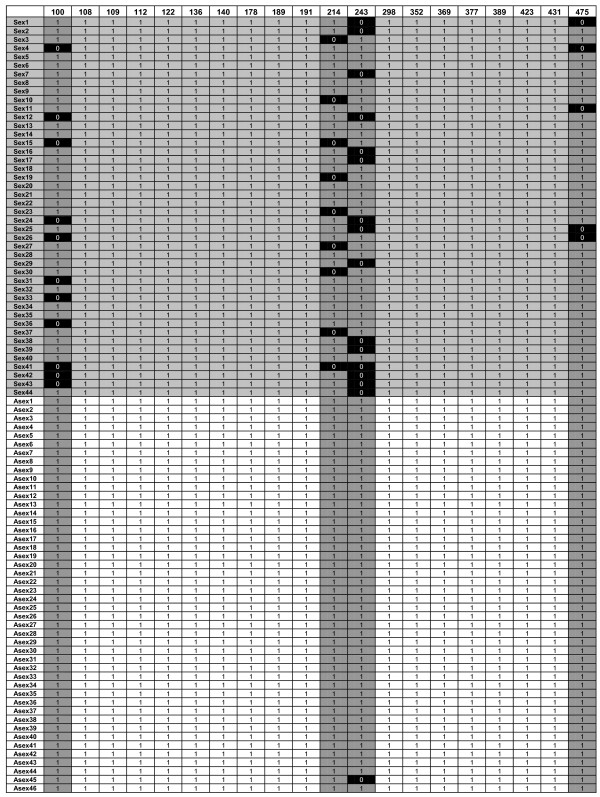
**Example of the data matrix generated for each family based on transposon display data (*Tc1NA2.1 *shown here)**. Each row represents one lineage (sexuals in light gray, asexuals in white). Each column represents a locus occupied in the ancestor (numbers indicate size of fragment produced by transposon display) and dark gray columns represent high loss loci (losses observed in more than three lineages at a given locus).

**Figure 4:**
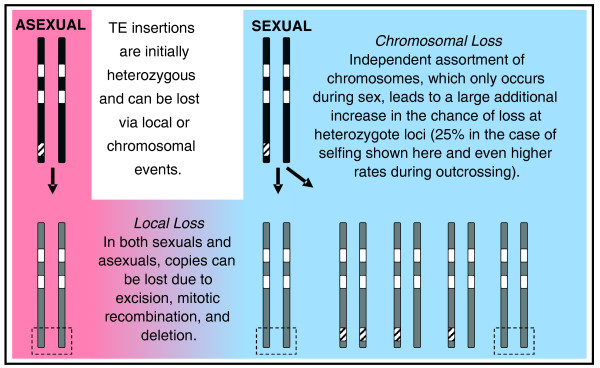
**Schematic of how TE copies are lost in asexually versus sexually reproducing organisms outlining the significant increase in rates of loss introduced by independent assortment during meiosis**. Dark gray bars represent parental chromosomes, white rectangles represent old insertions, hashed rectangles represent new insertions, light gray bars represent offspring chromosomes after local or chromosomal loss (indicated by dashed boxes).

**Figure 5:**
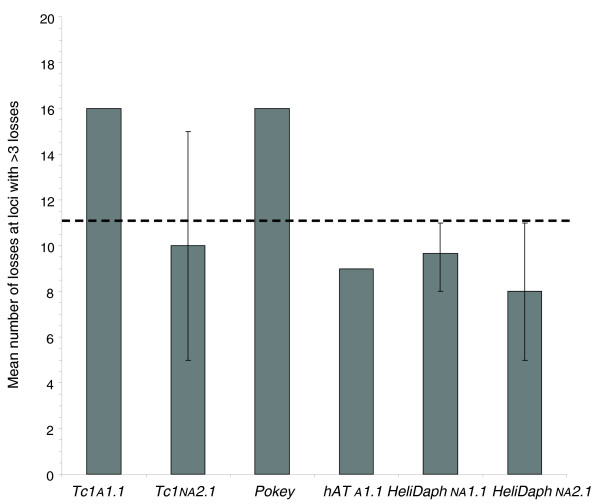
**Mean number of losses observed at high loss loci within each family in sexual lines (bars represent ranges)**. The dashed line shows the predicted number of losses at heterozygous loci (11.25) based on independent assortment after one bout of sex for the number of lineages assayed (n = 44 or 45 depending on the TE family).

The number of loci occupied by DNA transposons was assayed using TD after approximately 40 generations of mutation accumulation and rates of both loss and gain were calculated and compared between sexuals and asexuals. Rates of loss (per element per generation) were much higher than rates of gain (Table 2) but were almost completely restricted to lineages that had undergone at least one bout of sexual reproduction (Figure 3; Additional file 4). For each family, element loss was not random among occupied loci, but instead was usually observed at a subset of specific loci across all lines (Figure 3), suggesting that these sites were heterozygous in the ancestor used to start the experiment and that losses represent the segregation of heterozygotic copies after meiosis (Figure 4). Independent assortment among chromosomes during selfing (as seen here) would result in a 25% chance of loss of a heterozygotic TE and even higher rates of loss when outcrossing. Concurrently, redistribution of heterozygous copies after sex would result in homozygosity 25% of the time in the case of selfing, which would dramatically reduce the risk of future loss because of homologue-dependent DNA repair. The frequency of loss at designated 'high-loss loci' (where an ancestrally occupied site demonstrates a loss in more than three lineages) among sexual lines conformed well to predictions of approximately 25% chance of loss based on independent assortment in all families of DNA transposons assayed (Figure 5). The three families in which the number of losses at these loci occasionally exceeded expectations based on independent assortment alone (*Tc1A1.1*, *Tc1NA2.1*, and *Pokey*) are also the families for which loss was observed in asexual lineages (Table 2). This indicates the number of losses observed among sexual lines for these three families may represent a combination of both local removal (excision, mitotic recombination, or deletion) and chromosomal loss (via independent assortment).

**Table 2:** Rates of loss per ancestral insertion per generation (with standard errors) for six families of DNA transposons across mutation-accumulation lineages where sex was promoted (sexuals) and prohibited (asexuals). Number of high-loss loci (loci where losses were observed in more than three lineages) and *t*-test results are shown.

	N (sex/asex)	Number of high loss loci	Rate of loss (per element per generation)			
					
Element			Sexuals	Asexual	*T*	*P*
*Tc1A1.1*	46/46	1	0.00040 (± 0.00009)	0.00021 (± 0.00009)	2.0	0.02
*Tc1NA2.1*	44/46	4	0.00051 (± 0.00008)	0.000015 (± 0.00002)	6.3	<0.000001
*Pokey*	47/46	1	0.00078 (± 0.00002)	0.000058 (± 0.00006)	3.3	0.0007
*hATA1.1*	47/46	1	0.00094 (± 0.0003)	0	3.4	0.0004
*HeliDaphNA1.1*	47/46	3	0.00046 (± 0.0008)	0	5.4	<0.000001
*HeliDaphNA2.1*	46/46	6	0.0020 (± 0.0003)	0	7.8	<0.000001

In order to compare rates of loss with those reported previously in the literature, it is important to exclude sexual lines where estimates are conflated by the dramatic loss due to independent assortment. Losses observed in asexual lineages are not only attributable to excision, however, and could be alternatively explained by random spatial processes, such as deletion or mitotic recombination (known to occur in *D. pulex *[23]). These alternatives seem unlikely, however, because losses among asexuals were observed only for three DNA transposon families, and these same families also had rates of loss in sexuals in excess of the predictions based on independent assortment. Regardless of the mechanism of local loss, the rates calculated for asexuals (that is, excluding the impact of independent assortment) are on par with those previously reported in the literature (approximately 10^-5 ^and 10^-6 ^[24,25]).

Across the six element families, there was only evidence for one potential germline gain of a DNA transposon and it was observed in the *hATA1.1 *family. This new peak was robust and was observed in five separate TD replicates (Figures S4 and S5 in Additional files 5 and 6, respectively), and was not accompanied by a loss of another peak (which could be an indication of a simple mutation at the downstream restriction site). One germline gain among all lineages surveyed yields an estimate of the transposition rate for this family of 9.8 × 10^-5 ^per element per generation (lower than previously reported rates of approximately 10^-4 ^based only on a single observation; reviewed in [24,25]). Although we cannot conclude whether rates of transposition differ with and without sex, this gain suggests *hAT *elements in *D. pulex *are actively transposing.

**Figure 6:**
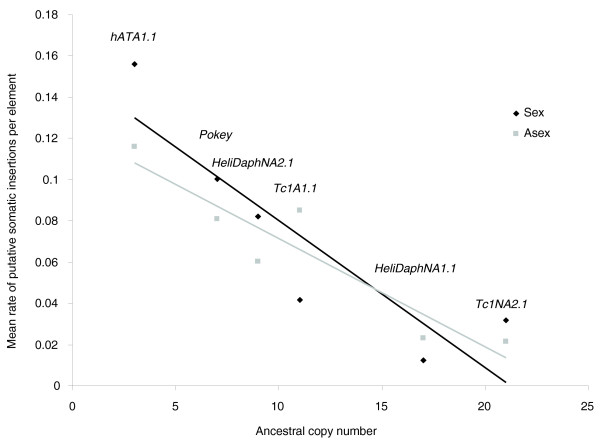
**Mean rate of putative somatic gains per element decreases with ancestral copy number for each DNA transposon family surveyed (lines indicate a best fit for each treatment; sample sizes for each family presented in Table 2)**.

In addition to this potential germline gain, TD revealed many new, robust peaks that could not be replicated in every reaction. Because these peaks were above thresholds for inclusion, but were not observed consistently, they were scored as new putative somatic insertions (Additional file 6). Somatic transposition is known to occur in many systems (for example, [26-28]), although theory suggests it would be selected against over time because it carries phenotypic negative costs with no heritable gains for the TE. There was no difference between sexual and asexual lineages in the rate of gain of putative somatic copies for four families, but in *Tc1A1.1 *and *Helidaph NA1.1 *(among the largest families), rates per element were higher in asexuals than in lineages where sex had occurred (Supplemental Table S2 in Additional file 1). Although one can envision a scenario where, over time, asexual lineages may accumulate mutations inactivating loci responsible for suppression of somatic activity, it seems unlikely to have occurred on the timescale of this experiment. Across families, there is a striking negative correlation between the rate of putative somatic transposition per copy and TE family size (Figure 6; regression for pooled treatments, R^2 ^= 0.66, df = 1, F = 19.38, and *P *= 0.001). This relationship could be explained if larger families have co-evolved with the host genome for a longer period of time, and therefore are subject to an increased level of silencing from the host, thereby reducing somatic activity. Alternatively, high copy number families may simply be composed of more inactive copies, resulting in the appearance of lower somatic activity per copy.

## Discussion

### TE composition and potential for activity

We found representative elements from the ten currently recognized Class 2 superfamilies in the genome of *D. pulex*. The proportion of the genome composed of DNA transposons, 0.72%, is within the range of most other arthropods for which such data exist (for example, the *Drosophila melanogaster *genome is composed of 0.31% DNA transposons [29] and that of *Apis mellifera *is 1% DNA transposons [30]). Based on four lines of evidence, it appears that the families assayed here are currently active in the genome of *D. pulex*. First, based on the structure of the elements (intact ORFs, where applicable, and percent identity between copies) there is sequence evidence indicating the elements have been active relatively recently and may be capable of further mobilization. Second, there is evidence for a germline gain of a copy of a *hAT *element that suggests this family is actively transposing in *D. pulex*. Third, evidence for possible excision was found for three of the six families based on the observed loss of copies in purely asexual lineages (*Tc1A1.1*, *Tc1NA2.1*, and *Pokey*) and an excess of loss in sexuals above that which would be predicted by independent assortment alone. Fourth, the observation of putative somatic insertions in all six families suggests these families are capable of activity and could mobilize in the germline as well.

### The role of recombination in long-term TE dynamics

The dynamics observed in lineages where sex was either prohibited or promoted supports the prediction that reproductive mode does, in fact, strongly influence patterns of TE proliferation in the genome. The major source of these differences in DNA transposon abundance appears to be the large impact of independent assortment of chromosomes on heterozygous loci. The observation of losses at or near the levels predicted by independent assortment during selfing (approximately 25%) not only means that this mechanism can hasten the loss of heterozygous DNA transposon copies, but simultaneously suggests an increased rate of homozygosity (also approximately 25%) at these loci as well. This elevated risk of homozygosity in sexuals has two major consequences. The first is the potentially large phenotypic impact resulting from the unmasking of recessive, negative effects of the DNA transposon once the insert is present at the same locus on both chromosomes. The second is the dramatic reduction in the probability of future loss of the DNA transposon at this particular locus once it occupies the site on both homologues, even if it does not have large phenotypic effects in the homozygous state. Homozygosity eliminates the chance of loss by mitotic recombination and reduces the chance of loss by excision because both homologs harbor the DNA transposon copy. Even if one copy is excised, homologue-dependent DNA repair can result in its reconstitution because the existing copy is used as a template to repair the site after removal [31]. Because DNA repair is typically imperfect, it is possible that the reconstituted copy will not be full length, although it may still be capable of transposition.

The chance of a heterozygous insertion becoming homozygous via sex decreases when effective population size is large. Despite the likelihood of large global effective population size for *Daphnia*, the probability of an insertion becoming homozygous in a given generation could be significant given the habitat for *D. pulex *is typically small, ephemeral ponds. It has been suggested previously that avoiding the risk of homozygosity of deleterious mutations may explain the repeated success of asexuals in nature [32]. Whereas any new insertions in a sexually recombining genome can become homozygous, asexuals carry only the homozygous insertions they inherited from their sexual progenitor (the so-called 'lethal hangover' from sex [33]). Populations found in nature may represent those isolates descended from sexual progenitors with particularly low mutation loads (but see [34]). These asexual lineages may be quite competitive with sexuals not only because they avoid many of the classic costs associated with sex, but also because they have a reduced risk of future homozygosity at mutated loci, such as those where TEs have inserted. The benefits (and risks) of genetic segregation and recombination during sex can be mimicked in asexuals via mitotic recombination [35], although the frequency of mitotic recombination in *Daphnia *(shown in both sexuals and asexuals [23]) should be lower than the frequency of meiotic recombination. Although occasional sex is the norm in *D. pulex*, populations where it has been lost have been recorded frequently [36]. Over long time periods, the impact of independent assortment on new heterozygous copies clearly could result in considerably different distributions and abundance of TEs in sexuals versus asexuals. Because obligately asexual *D. pulex *populations occur naturally, it is possible to further investigate the mutational consequences of switching reproductive modes and therefore the evolution of sex based on TE accumulation in this species at the population level. Such analyses have been performed and suggest that, despite the short-term advantage observed here, cyclical parthenogens in nature accumulate more TEs than their asexual counterparts [37,38].

## Conclusions

The aim of this study was to characterize DNA transposons and their dynamics across families in the cyclical parthenogen *D. pulex*. The variation among DNA transposon families in abundance reveals patterns of proliferation do not appear to correlate strongly with phylogenetic relatedness among TEs (for example, families within the same superfamily do not necessarily behave similarly), but instead suggest other factors, such as copy number, may play a role. Differences between lineages where sex was prohibited or promoted indicate that recombination has significant effects on TE dynamics, most notably via the redistribution of copies due to independent assortment. Whether or not sex influences rates of excision or germline transposition rate remains an open question and would require a longer period of mutation accumulation to detect. This analysis represents the first multi-element comparison in a cyclical parthenogen and crustacean and suggests TE dynamics in this species vary based on family size and may be significantly impacted by differences in reproductive mode. Our data suggest there may be significant consequences in terms of TE abundance and distribution over long time periods in natural populations capable of reproducing with and without sex.

## Materials and methods

### Transposable element identification

The v1.1 draft genome sequence assembly of *D. pulex *was scanned for protein coding TEs using a homology-based approach. Queries representing the most well-conserved region of the encoded proteins of all known eukaryotic Class 2 DNA transposons were used in TBLASTN searches of the pre-release genome. Contigs identified containing sequences with homology (e-values < 0.01) to known TE proteins were scanned for signature structural characteristics (for example, target site duplications and terminal inverted repeats). Conceptual translations were performed with the ExPASy translation program [39,40] and NCBI ORF Finder [41]. Alignments of DNA transposon proteins with representative known TE proteins were constructed using a combination of ClustalW embedded in MEGA 4.0 [42], BLASTN [43], and MUSCLE [44]. Canonical elements were used to mask the genome (using RepeatMasker [45]) and copy number and genome content estimates were compiled based on these and local BLAST results using default parameters. Repeats were filtered to include only those with a minimum length of 50 bp, >20% of the length of the query, and >70% similarity between query and hit to compile data for Table 1. DNA transposons containing full-length ORFs (within the published standard range, intact target site duplications, or other evidence of potential recent activity) were assayed experimentally (see below). Families that amplified and appeared variable among a subset of lineages (that is, showed evidence for presence-absence polymorphism after approximately 20 generations in a subset of MA lines) were selected for the survey.

### Mutation-accumulation experiment

MA lines were initiated in 2004 from the sequenced isolate of *D. pulex *dubbed The Chosen One (TCO). TCO was collected from Slimy Log Pond, OR in 2000 and maintained in the laboratory until initiation of the experiment. Third-generation descendants of a single female were used to initiate experimental lines, which were clonally propagated each generation soon after first clutch was produced by the focal female in each line, each generation (generation times were approximately 12 days at 20°C). Lines were maintained at constant temperature (20°C) and fed *Scenedesmus obliquus *three times per week. When focal animals were dead or sterile, a back-up system was used to propagate the line. The back-up system consisted of simultaneously isolating two sibling animals during each transfer. These animals were stored in 50 ml uncapped plastic tubes and fed and maintained in the same manner as the focal individuals. Isolating these individuals in parallel allowed us to rescue a line if the focal individual died. In extreme, rare cases, where both the focal individual and the back-up individuals were dead, the line was propagated from beakers of animals from previous generations of the lineage also maintained in the lab (at 10°C) by selecting a random individual to bottle-neck the population and continue the line.

All lines were propagated by transferring either one or five (alternating each generation) random 1- to 2-day-old live female offspring to a new beaker. Females produced one to two clutches of asexual offspring, which were used to propagate each line each generation. The subsequent crowding was used to generate cues inducing meiosis, after which females produced male offspring and then haploid resting eggs, which were fertilized when the females mated with their sons. These eggs were collected and stored in tissue culture plates with 5 to 10 ml H_2_0 per well at 4°C. This occurred typically 4 to 5 days after asexually produced young had been born and transferred to a new beaker to propagate the original asexual line. Any ephippia that hatched after exposing eggs to short, intermittent periods of warmer temperatures (20°C) were used to initiate sexual sublines of asexual lineages. Sexual sublines (identified by their source asexual lineage and the generation at which the bout of sexual reproduction had occurred) were occasionally induced to reproduce sexually a second time, although only three such lineages were included in this survey. Other than hatching (and the conditions immediately preceding hatching), sexual sublines were maintained in the same manner over the course of the experiment as asexuals. The total number of lines used in the assay was 94, with 47 'asexual' lines being propagated exclusively asexually for the duration of the experiment compared to an additional 47 'sexual' lines that were maintained in the same way, but with the occurrence of at least one bout of sex.

Tissue for transposon display was collected after approximately 40 generations and was extracted from 5 to 10 individuals (clonally produced sisters) for each lineage individually. Genomic DNA was extracted by grinding adult tissue in a CTAB (cetyltrimethylammonium bromide) buffer [46] and incubating at 65°C for 1 h. Samples were extracted with a chloroform/isoamyl alcohol solution (1:24) and the DNA was precipitated and washed using 100% and 70% ethanol solutions, respectively. The DNA was resuspended in 50 μl of ddH2O and used for subsequent reactions.

### Transposon display

TD is a PCR-based technique developed by the *Daphnia *Genomics Consortium [45] to estimate the number of TE insertion sites per genome for a given family of elements. TD was performed by using the restriction enzyme EcoR1 to digest genomic DNA from each sample (n = 94; 5 μl template DNA (ranging from approximately 40 to 80 ng/μl), 30 μl H_2_O, 4 μl manufacturer supplied buffer; 0.5 μl EcoR1). Typically, TD is conducted using a 4-bp cutter but our preliminary results indicated the restriction-ligation reaction worked best with EcoR1. Given that our ability to detect fragments is improved by the use of fragment analysis technology and software (described below) and a longer calibration ladder than previous studies (1,200 bp versus 500 bp [37]), we used this digest even though it would undoubtedly result in a longer average fragment length. Digests were performed for 6 h at 37°C followed by 22 minutes at 80°C. Adaptors consisting of approximately 20 bp oligonucleotide pairs with a non-complementary mid-portion were ligated on to the ends of each fragment after the digest (7.5 μl H_2_O, 0.5 T4 ligase, 1 μl manufacturer supplied buffer, 1 μl adaptor (50 mM) added to each restriction digest reaction; 16 h ligation at room temperature). Element-containing fragments were amplified via nested PCR using a fluorescent element-specific primer (forward) and a reverse primer complementary to the non-complementary mid-portion of the ligated adaptors (Supplemental Table S3 in Additional file 1). Only fragments of the genome containing copies of the element being assayed are amplified because the reverse primer cannot anneal unless the element-specific primer binds and elongates and only TE-bearing fragments are scored because only the TE-specific primer is fluorescently labeled. Conditions for the first and second round of PCR were as follows: initial denaturation at 94°C for 3 minutes, followed by 24 cycles of denaturation at 94°C for 30 s, annealing at 5°C below the melting temperature for the element-specific primer (30 s), and elongation at 72°C for 1 minute, ending with a 5-minute elongation step at 72°C. The second round of PCR used a fluorescently labeled (6FAM) element-specific primer slightly more towards the 3' end of the conserved region of the element and the same thermocycler program.

Fragments resulting from the nested PCR were run out on an ABI 3730 Genotyper and analyzed using Genemapper with the LIZ 1200 size standard. All samples were run in triplicate and data were scored manually. Because all lines were initiated from a single common ancestor, differences in banding pattern among descendent lineages indicated loss and/or gain of copies of individual elements within the genome. Losses were scored based on the absence of bands at locations where, in the majority of the samples, peaks were typically found. Gains were only considered germline gains if new peaks were present in all three replicates. New peaks that were above threshold levels but not present in all three replicates tended to be lower height, but still robust (Additional file 6), and were scored as putative somatic insertions. This technique is sensitive but provides a lower-bound estimate for activity levels because long fragments may not amplify due to PCR bias and because of the conservative nature of the scoring regimen. In order to verify that fragments amplified using transposon display indeed represented the 3' end of the specific TE family for which the primer was designed, additional PCR reactions were performed using non-fluorescent element-specific primers under the same conditions. These fragments were cloned using the Invitrogen TOPO PCR cloning kit™ (Invitrogen, Carlsbad, CA, USA) following the manufacturer's protocols. Cloned fragments were PCR amplified using the reverse primer from the initial secondary PCR reaction (complementary to the adaptor) and the successful amplicons were sequenced using ABI's BigDye™ sequencing mix (1.4 μl template PCR product, 0.4 μl BigDye, 2 μl manufacturer supplied buffer, 0.3 μl reverse primer, 6 μl H_2_O; thermocycler program starting with 2 minutes denaturation (96°C) followed by 30 cycles alternating between 96°C (30 s) and 60°C (4 minutes), and cool down at 10°C for 3 minutes). Sequencing reactions were run on an ABI 3730 and sequences were trimmed using CodonCode Aligner (CodonCode Corporation, Dedham, MA, USA) and were aligned and analyzed using MEGA 4.0 [42]. Cloning and sequencing of fragments from TD reactions revealed that all PCR amplicons do, indeed, represent fragments containing the 3' end of the TE family from which the primer was designed, although it is truncated in some cases (data not shown). Not enough clones were sequenced to represent all the inserts detected using TD and putative somatic insertions are swamped by germline copies. Sequenced clones, however, represent a number of independent insertions for each family of elements assayed and the amplification and sequencing process enriches for fragments for which the primer has high affinity, not spurious PCR artifacts that may occasionally occur. The scoring criteria used for TD was conservative (see Additional file 4s for rubric).

### Data analysis

A limitation of the TD technique is its inability to distinguish between loci that are heterozygous or homozygous for a given insertion. Insertions that appeared in the same location on the trace file in multiple lineages are presumed to be ancestral (that is, they were present in the single individual ancestor to the experimental lines, and may only be lost over time, not gained). In addition, because of the pattern revealed in lines in which sex had occurred, it was possible to detect sites that were likely heterozygous in the ancestor based on high rates of loss. The insertion profiles generated for each MA line (presence-absence matrices for each TE family) were analyzed by calculating the mean corrected rates of loss based on the number of losses per lineage per generation per ancestral element copy. Rates of putative somatic gain were calculated by dividing the number of new, non-replicable peaks by the number of ancestral peaks. Mean rates were compared between treatments (sexuals and asexuals) within each element family using a *t*-test and across families using analysis of covariance (ANCOVA) with ancestral copy number as a covariate, and across families using regression.

## Abbreviations

bp: base pair; MA: mutation accumulation; ORF: open reading frame; TD: transposon display; TE: transposable element.

## Authors' contributions

SS, ML, and EP conceived of the study. SS and EP carried out the bioinformatic analysis. SS conceived of and SS and EC carried out the experimental work. SS performed the molecular and statistical analyses. SS, ML, and EP wrote the manuscript. All authors read and approved the final manuscript.

## Supplementary Material

Additional file 1 Tables S1, S2, and S3. Table S1 includes the best hits based on TBLASTN for each DNA transposon family identified in the *D. pulex *genome. Table S2 includes rates of putative somatic gains per ancestral insertion in six families of transposable elements across mutation-accumulation lineages where sex was promoted (sexuals) and prohibited (asexuals). Table S3 contains primer sequences. Click here for file

Additional file 2 Supplemental Data S1 and Table S4. Data S1 is a FASTA file of canonical *D. pulex *Class 2 DNA transposons. Table S4 lists the scaffold and coordinates for all regions of the genome masked by canonical representatives of the DNA transposons in *D. pulex *(note that these are not filtered based on size or similarity). Click here for file

Additional file 3 Alignments showing conserved protein-coding regions for representatives from each major family of TE identified in the *D. pulex *genome **(a) ***Tc1*/*mariner *superfamily, **(b) ***Pogo *(subfamily of *Tc1*/*mariner*), **(c) ***Ant *(subfamily of *Tc1*/*mariner*), **(d) ***hAT*, **(e) ***P-element*, **(f) ***Mutator*, **(g) ***PIF*/*Harbinger*, **(h) ***Merlin*, **(i) ***CACTA*, **(j) ***Maverick*. Click here for file

Additional file 4 Transposon display results for five DNA transposon families assayed across lineages where sex was promoted or prohibited including scoring rubric. Click here for file

Additional file 5 Trace file from transposon display reactions showing evidence for a putative germline gain of a copy of the *hATA1.1 *element. The top four traces show separate runs for the sample, indicating a new, replicable peak is found at 385 bp (red box). A putative somatic insertion is also visible in the top trace file (at 436 bp; blue box) where a new peak was observed in only this replicate. The bottom two traces are from another line and represent the ancestral state for the lineages lacking these new copies. Click here for file

Additional file 6 Peak heights for transposon display performed for *hATA1.1 *using MA lines of *D. pulex*. All peaks were above minimum thresholds for inclusion (see Additional file 4). Peaks were scored based on replicability as a: 1, ancestral insertion (common across lineages and replicable within a lineage); 2, new germline insertion (found only in one lineage, replicable in all TD reactions); or 3, putative somatic insertion (unique to one lineage and not replicable in three TD reactions). Peak heights are based on heights scored by Genemapper software for each of three TD replicate reactions performed for each lineage, with the heights for the new germline insertion shown in pink (described in Additional file 5). Lines represent a best fit for each group. Click here for file
