# Supplementary material for: DNA transposons and the role of recombination in mutation accumulation in Daphnia pulex
Source: Genome Biol. 2010 Apr 30;11(4):R46. doi: 10.1186/gb-2010-11-4-r46 (PMC2884549; doi:10.1186/gb-2010-11-4-r46)
Supplement: Additional file 1 — Tables S1, S2, and S3. Table S1 includes the best hits based on TBLASTN for each DNA transposon family identified in the D. pulex genome. Table S2 includes rates of putative somatic gains per ancestral insertion in six families of transposable elements across mutation-accumulation lineages where sex was promoted (sexuals) and prohibited (asexuals). Table S3 contains primer sequences. [file gb-2010-11-4-r46-S1.doc]

**Additional file 1: Supplemental Tables**

Supplemental Table S1. DNA transposon families identified in *D. pulex* listed by subclass and superfamily. Best hits from NCBI and Repbase with corresponding E value (expectation values, [E]), identity (similarity at the amino acid AA level, [I]), and positives (proportion of positions with similar physico-chemical properties, [P]) are also shown.

| **Superfamily** | **Estimated protein-coding copies** | **Element type** | **Best hit**  (NCBI protein database)  (*Repbase*) |
| --- | --- | --- | --- |
| Subclass 1 |  |  |  |
| CACTA | 130 | CactaA1.1 | XP_001922528.1, DANRE, E = 1^e-78^ I = 197/622 (31%), P = 313/622 (50%)  *EnSpm-10, HYDMA, E = 4^e-78^*  *I = 67/177 (37%), P = 96/177 (54%)* |
|  |  | CactaA2.1 | XP_001951123.1, ACYPI, E = 4^e-90^  I = 211/628 (33%), P = 338/628 (53%)  *EnSpm-10, HYDMA, E = 4^e-50^*  *I = 34/80 (42%), P = 52/80 (65%)* |
|  |  | CactaA3.1 | XP_001922528.1, DANRE, E = 1^e-85^ I = 184/428 (42%), P = 261/428 (60%)  *ENSPM-6, DANRE, E = 2^e-90^*  *I = 70/164 (42%), P = 103/164 (62%)* |
|  |  | CactaA4.1 | XP_001922528.1, DANRE, E = 1^e-72^, I = 190/568 (33%), P = 293/568 (51%)  *EnSpm-5, HYDMA, E = 6^e-64^*  *I = 33/69 (47%), P = 46/69 (66%)* |
|  |  | CactaA5.1 | EEC19829.1, IXOSC, E = 8^e-76^ I = 186/567 (32%), P = 289/567 (50%)  *EnSpm-2, HYDMA, E= 1^e-87^*  *I = 74/167 (44%), P = 106/167 (63%)* |
|  |  | CactaA6.1 | XP_001951183.1, ACYPI, E(2) = 5^e-67^ I = 144/466 (30%), P = 240/466 (51%)  *EnSpm-2, HYDMA, E = 9^e-77^*  *I = 66/166 (39%), P = 102/166 (61%)* |
|  |  | CactaA7.1 | EEC18565.1, IXOSC, E = 1^e-47^ I = 99/240 (41%), P = 143/240 (59%)  *EnSpm-1, AEDAE, E = 5^e-45^*  *I = 62/181 (34%), P = 98/181 (54%)* |
|  |  | CactaA8.1 | XP_001946455.1, ACYPI, E = 1^e-16^ I = 81/285 (28%), P = 134/285 (47%)  *EnSpm-4, HYDMA, E = 4^e-09^*  *I = 21/62 (33%), P = 32/62 (51%)* |
|  |  | CactaA9.1 | EEC19714.1, IXOSC, E = 2^e-31^ I = 73/209 (34%), P = 102/209 (48%)  *EnSpm-2, NASVI, E = 3^e-26^*  *I = 26/63 (41%), P = 35/63 (55%)* |
|  |  | CactaA10.1 | CAK26786.1, STRPU, E = 7^e-19^  I = 68/220 (30%), P = 107/220 (48%)  *EnSpm-12, HYDMA, E = 2^e-25^*  *I = 37/133 (27%), P = 57/133 (42%)* |
|  |  |  |  |
| hAT | 64 | hATA1.1 | CAP94930.1, PENCH, E = 6^e-34^ I = 145/594 (24%), P = 249/594 (41%)  *HAT1, MEDTR, E = 4^e-29^*  *I = 27/66 (40%), P = 37/66 (56%)* |
|  |  | hATNA1.1 |  |
|  |  | hATA2.1 | CAP94930.1, PENCH, E = 6^e-36^ I = 90/353 (25%), P = 172/353 (48%)  *HAT1, MEDTR, E = 1^e-21^*  *I = 41/126 (32%), P = 72/126 (57%)* |
|  |  | hATA3.1 | AAS21248.1, ANOGA, E = 1^e-51^ I = 177/639 (27%), P = 300/639 (46%)  *hAT-2, ACYPI, E = 7^e-51^*  *I = 32/93 (34%), P = 55/93 (59%)* |
|  |  | hATA4.1 | NP_001064320.1,ORYSA, E = 2^e-18^ I = 101/407 (24%), P = 171/407 (42%)  *TAG2-hAT, ARATH, E = 8^e-24^*  *I = 32/66 (48%), P = 42/66 (63%)* |
|  |  | hATA5.1 | CAE05040.1, ORYSA, E = 8^e-31^ I = 115/428 (26%), P = 193/428 (45%)  *AC9-hAT, ZEAMA, E = 9^e-31^*  *I = 31/89 (34%), P = 50/89 (56%)* |
|  |  |  |  |
| Merlin | 25 | MerlinA1.1 | XP_001678363.1, CAEBR, E = 7^e-19^ I = 52/150 (34%), P = 70/150 (46%)  *MERLIN1, SCHME, E = 4^e-29^*  *I = 33/59 (55%), P = 41/59 (69%)* |
|  |  |  |  |
| Mutator | 100 | MutatorA1.1 | EEA45120.1, BRAFL, E = 5^e-28^ I = 59/142 (41%), P = 84/142 (59%)  *MuDR1x, SCHME, E = 3^e-19^*  *I = 27/112 (24%), P = 51/112 (45%)* |
|  |  | MutatorA2.1 | EEA45120.1, BRAFL, E = 4^e-24^ I = 86/302 (28%), P = 127/302 (42%)  *MuDR2x, ACYPI, E = 6^e-14^*  *I = 31/82 (37%), P = 44/82 (53%)* |
|  |  | MutatorA3.1 | EEA45120.1, BRAFL, E = 1^e-40^ I = 67/153 (43%), P = 85/153 (55%)  *MuDR1x, SCHME, E = 3^e-23^*  *I = 28/74 (37%), P = 42/74 (56%)* |
|  |  | MutatorA4.1 | EEA45120.1, BRAFL, E = 2^e-18^ I = 54/146 (36%), P = 82/146 (56%)  *MuDR5x, ACYPI, E = 1^e-07^*  *I = 15/43 (34%), P = 24/43 (55%)* |
|  |  | MutatorA5.1 | EEA45120.1, BRAFL, E = 1^e-33^ I = 80/170 (47%), P = 112/170 (65%)  *MuDR4x, SCHME, E = 8^e-11^*  *I = 18/40 (45%), P = 23/40 (57%)* |
|  |  | MutatorA6.1 | EEA56326.1, BRAFL, E = 8^e-16^ I = 56/179 (31%), P = 92/179 (51%)  *MuDR4x, SCHME, E = 0.026*  *I = 10/12 (83%), P = 10/12 (83%)* |
|  |  | MutatorA7.1 | EEA45120.1, BRAFL, E = 3^e-12^ I = 41/144 (28%), P = 65/144 (45%)  *MuDR11x, ACYPI, E = 4^e-08^*  *I = 21/52 (40%), P = 32/52 (61%)* |
|  |  | MutatorA8.1 | EEA45120.1, BRAFL, E = 6^e-28^ I = 65/152 (42%), P = 96/152 (63%)  *MuDR1x, SCHME, E = 4^e-13^*  *I = 15/49 (30%), P = 30/49 (61%)* |
|  |  | MutatorA9.1 | EEA56326.1, BRAFL, E = 2^e-16^ I = 51/132 (38%), P = 78/132 (59%)  *MuDR9x, ACYPI, E = 0.29*  *I = 13/27 (48%), P = 15/27 (55%)* |
|  |  | MutatorA10.1 | EAT44804.1, AEDAE, E = 1^e-4^ I = 38/101 (37%), P = 54/101 (53%)  *MuDR3x, SCHME, E = 0.001*  *I = 18/40 (45%), P = 25/40 (62%)* |
|  |  |  |  |
| P-element | 59 | PelementA1.1 | XP_001947289.1, ACYPI, E = 3^e-24^  I = 121/448 (27%), P = 191/448 (42%)  *Galileo-P, DROWI, E = 8^e-12^*  *I = 31/92 (33%), P = 54/92 (58%)* |
|  |  | PelementA2.1 | EEC16476.1, IXOSC, E = 1^e-23^  I = 69/197 (35%), P = 105/197 (53%)  *P-3, ACYPI, E = 2^e-10^*  *I = 27/87 (31%), P = 38/87 (43%)* |
|  |  | PelementA3.1 | XP_001948762.1, ACYPI, E = 5^e-16^ I = 89/335 (26%), P = 149/335 (44%)  *P-3, ACYPI, E = 4^e-08^*  *I = 28/85 (32%), P = 39/85 (45%)* |
|  |  | PelementA4.1 | EEC05690.1 IXOSC, E = 2^e-21^, I = 69/222 (31%), P = 112/222 (50%)  *P-14, HYDMA, E = 2^e-16^*  *I = 23/48 (47%), P = 30/48 (62%)* |
|  |  | PelementA5.1 | EEC16581.1, IXOSC, E = 7^e-11^ I = 45/106 (42%), P = 61/106 (57%)  *P-34, HYDMA, E = 4^e-12^*  *I = 23/57 (40%), P = 33/57 (57%)* |
|  |  | PelementA6.1 | XP_001950144.1, ACYPI, E = 2^e-23^  I = 70/235 (29%), P = 117/235 (49%)  *Galileo-P, DROBU, E = 1^e-17^*  *I = 30/81 (37%), P = 39/81 (48%)* |
|  |  | PelementA7.1 | ABC16631.1, ANOGA, E = 2^e-11^ I = 85/337 (25%), P = 135/337 (40%)  *P-32, HYDMA, E = 1^e-08^*  *I = 19/69 (27%), P = 37/69 (53%)* |
|  |  | PelementA8.1 | XP_001950144.1, ACYPI, E = 1^e-35^ I = 123/420 (29%), P = 200/420 (47%)  *P-36, HYDMA, E = 1^e-15^*  *I = 24/64 (37%), P = 32/64 (50%)* |
|  |  | PelementNA9.1 |  |
|  |  |  |  |
| PIF | 44 | PIFA1.1 | XP_795950, STRPU, E = 2^e-44^  I = 62/136 (45%), P = 86/136 (63%)  Harbinger3, DANRE, E = 2^e-41^  I = 27/45 (60%), P = 32/45 (71%) |
|  |  | PIFA2.1 | XP_001944013.1, ACYPI, E = 2^e-10^ I = 43/166 (25%), P = 73/166 (43%)  *Harbinger1, DANRE, E = 1^e-17^*  *I = 33/63 (52%), P = 38/63 (60%)* |
|  |  |  |  |
| PiggyBac/  TTAA[[1]](#endnote-2) | 188 | Pokey | AAM76341.1, DPULI, E = 0 I = 487/487 (100%), P = 487/487 (100%)  *Pokey, DPULI, E = 0.0*  *I = 832/865 (96%), P = 832/865 (96%)* |
|  |  | TTAANA1.1 |  |
|  |  | TTAANA2.1 |  |
|  |  |  |  |
| Tc1/mariner | 270 | AntA1.1 | NP_284940.1, DEIRA, E = 2^e-39^ I = 106/300 (35%), P = 158/300 (52%) |
|  |  |  |  |
|  |  | PogoA1.1 | XP_001944813.1, ACYPI, E = 3^e-33^ I = 69/193 (35%), P = 108/193 (55%)  *Tc1-1, DANRE, E = 2^e-38^*  *I = 60/124 (48%), P = 76/124 (61%)* |
|  |  | PogoA2.1 | XP_001378790.1, MONDO, E = 2^e-36^ I = 105/372 (28%), P = 190/372 (51%)  *Mariner-27, SCHME, E(5) = 3^e-33^*  *I = 21/42 (50%), P = 32/42 (76%)* |
|  |  | PogoA3.1 | XP_589056.1, BOSTA, E = 3^e-36^ I = 77/229 (33%), P = 124/229 (54%)  *Mariner-2, NEMVE, E = 1^e-22^*  *I = 19/49 (38%), P = 28/49 (57%)* |
|  |  | Tc1A1.1 | AF099908_1, HAECO, E = 3^e-44^ I = 77/203 (37%), P = 120/203 (59%)  *Mariner-36, CAEBR, E = 3^e-58^*  *I = 61/153 (39%), P = 91/153 (59%)* |
|  |  | Tc1NA1.1 |  |
|  |  | Tc1NA2.1 |  |
|  |  |  |  |
| Sublclass 2 |  |  |  |
| Helitron | 380 | HeliDaphA1.1 | XP_001185162.1, STRPU, E = 3^e-166^  I = 327/763 (42%), P = 439/763 (57%)  *Helibat1, MYOLU, E = 0.0*  *I = 147/274 (53%), P = 203/274 (74%)* |
|  |  | HeliDaphA2.1 | NP_001042570.1, Oryza sativa, E = 8^e-04^ I = 24/68 (35%), P = 34/68 (50%)  *Helibat1, MYOLU, E = 0.0*  *I = 134/279 (48%), P = 190/279 (68%)* |
|  |  | HeliDaphNA1.1 |  |
|  |  | HeliDaphNA2.1 |  |
|  |  |  |  |
| Maverick | 4 | MaverickA1.1 | XP_001599618.1, NASVI, E = 1^e-40^ I = 160/560 (28%), P = 256/560 (45%)  *Polinton-10, NASVI, E = 7^e-52^*  *I = 57/130 (43%), P = 82/130 (63%)* |
|  |  | MaverickA2.1 | XP_001599249.1, NASVI, E = 5^e-44^ I = 108/345 (31%), P = 183/345 (53%)  *Polinton-10, NASVI, E = 6^e-47^*  *I = 50/113 (44%), P = 75/113 (66%)* |
|  |  | MaverickA3.1 | XP_001601554, NASVI, E = 9^e-58^ I = 64/159 (40%), P = 99/159 (62%)  *Polinton-10, NASVI, E = 4^e-88^*  *I = 60/126 (47%), P = 81/126 (64%)* |
|  |  | MaverickA4.1 | CAP39127, CAEBR, E = 6^e-05^  I = 29/95 (30%), P = 56/95 (58%)  *Polinton-3, TRICA, E = 6^e-08^*  *I = 18/54 (33%), P = 32/54 (59%)* |
|  |  |  |  |

Supplemental Table S2. Rates of putative somatic gains per ancestral insertion in 6 families of transposable elements across mutation-accumulation lineages where sex was promoted (sexuals) and prohibited (asexuals). Sample size, means (with standard errors), and comparisons using t-tests assuming equal variances are shown.

|  |  | Rate of putative somatic gain  (gains per element) | | | | |  | |  |
| --- | --- | --- | --- | --- | --- | --- | --- | --- | --- |
| Element | N  (sex/asex) | | Sexuals |  | Asexuals | df | | *t* | p |
| Tc1A1.1 | 46/46 | | 0.042  (±0.009) |  | 0.085 (±0.017) | 90 | | -2.29 | 0.012 |
| Tc1NA2.1 | 44/46 | | 0.017 (±0.003) |  | 0.015 (±0.004) | 87 | | 1.21 | 0.12 |
| Pokey | 47/46 | | 0.054 (±0.008) |  | 0.043 (±0.013) | 91 | | 0.69 | 0.25 |
| hATA1.1 | 47/46 | | 0.094 (±0.030) |  | 0.068 (±0.026) | 91 | | 0.70 | 0.24 |
| HelidaphNA1.1 | 47/46 | | 0.007  (±0.002) |  | 0.013 (±0.003) | 91 | | -1.71 | 0.044 |
| HelidaphNA2.1 | 46/46 | | 0.062 (±0.015) |  | 0.045 (±0.012) | 90 | | 0.87 | 0.19 |

Supplemental Table S3. Primers used in primary and secondary PCR reactions for transposon display for each family and adaptor sequences and reverse primer for an ECOR1 digest (all sequences 5’ to 3’).

| Oligos | Family | Primary primer | Secondary primer |
| --- | --- | --- | --- |
| Primers | Tc1A1.1 | CCAAGTGGTTCAAGAGTTATTGC | TTTTCAAGACACGTAGTGCCATA |
|  | Tc1NA2.1 | GAGACACTTTGGCCGTAAGC | TGTTAGAACCGCGGAAACACT |
|  | Pokey | GACAACGGTGGCCGAAACGCGG | TGGCCGAAACGCGGTTAGGCCG |
|  | hATA1.1 | CGCTCCAACCCAAGACTT | AGATCCTTTTCGACCCAGTG |
|  | HelidaphNA1.1 | AATCTTCAAGTAAGGTACTGGG | CGGGCAGATGATTRAGCTWCA |
|  | HelidaphNA2.1 | CATTCAAGTAAGGTAGGAAGAA | AAAGGAATTTACTGACTGGGCA |
|  |  |  |  |
|  | Reverse primer | GTAGACTGCGTACCAATTC |  |
|  |  |  |  |
| Adaptors | Adaptor Top | CTCGTAGACTGCGTACC |  |
|  | Adaptor Bottom | AATTGGTACGCAGTCTAC |  |

1. Elements flanked by TTAA nucleotides, but with insufficient evidence to be confirmed PiggyBac elements, were classified as TTAA elements, along with the previously identified Pokey element (Penton et al. 2002) known to exhibit this characteristic flanking sequence. [↑](#endnote-ref-2)
