# Supplementary material for: DNA transposons and the role of recombination in mutation accumulation in Daphnia pulex
Source: Genome Biol. 2010 Apr 30;11(4):R46. doi: 10.1186/gb-2010-11-4-r46 (PMC2884549; doi:10.1186/gb-2010-11-4-r46)
Supplement: Additional file 3 — Alignments showing conserved protein-coding regions for representatives from each major family of TE identified in the D. pulex genome (a) Tc1/mariner superfamily, (b) Pogo (subfamily of Tc1/mariner), (c) Ant (subfamily of Tc1/mariner), (d) hAT, (e) P-element, (f) Mutator, (g) PIF/Harbinger, (h) Merlin, (i) CACTA, (j) Maverick. [file gb-2010-11-4-r46-S3.pdf]

Supplemental Figure S2a. Alignment of Tc1/Mariner superfamily elements from multiple species, including two new families, Tc1_Dappu and Tc2_Dappu from *Daphnia pulex*.

Species abbreviations

Drosophila melanogaster	= DROME
Drosophila biofasciata	= DROBI
Drosophila helvetica	= DROHE
Drosophila willistoni	= DROWI
Drosophila hydei	= DROHY
Drosophila yakuba	= DROYA
Drosophila persimilis	= DROPE
Drosophila pseudoobscura	= DROPS
Drosophila buzzati	= DROBU
Drosophila ananassae	= DROAN
Mus musculus	= MUSMU
Danio rerio	= DANRE
Arabidopsis thaliana	= ARATH
Trichomonas vaginalis	= TRIVA
Fusarium oxysporum	= FUSOX
Podospora anserina	= PODAN
Caenorhabditis briggsae	= CAEBR
Caenorhabditis elegans	= CAEEL
Caenorhabditis remanei	= CAERE
Bombyx mori	= BOMMO
Rana pipiens	= RANPI
Salmo salar salar	= SALSA
Oncorhynchus mykiss	= ONCMY
Pleuronectes platessa	= PLEPL
Anopheles albimanus	= ANOAL
Anopheles gambiae	= ANOGA
Haemonchus contortus	= HAECO
Saccoglossus kowalevskii	= SACKO
Ixodes scapularis	= IXOSC
Halorubrum lacusprofundi	= HALLA
Hahella chejuensis	= HAHCH
Marinobacter aquaeolei	= MARAQ
Acidithiobacillus caldus	= ACICA
Aspergillus niger	= ASPNI
Talaromyces stipitatus	= TALST
Culex quinquefasciatus	= CULQU
Tribolium castaneum	= TRICA
Oryza sativa	= ORYSA
Aaedes aegypti	= AAEAE
Ciona intestinalis	= CIOIN
Trichuris muris	= TRIMU
Schistosoma japonicum	= SCHJA
Schistosoma mansoni	= SCHMA

Antirrhinum majus	= ANTMA
Bactrocera dorsalis	= BACDO
Phakopsora pachyrhizi	= PHAPA
Oryzias latipes	= ORYLA
Zea mays	= ZEAMA
Tolypocladium inflatum	= TOLIN
Strongylocentrotus purpuratus	= STRPU
Nematostella vectensis	= NEMVE
Hydra magnipapillata	= HYDMA
Glyptapanteles indiensis	= GLYIN
Acyrthosiphon pisum	= ACYPI
Branchiostoma floridae	= BRAFL
Candida albicans	= CANAL
Chelonus inanitus bracorvirus	= CHEIN
Cucumis melo	= CUCME
Culex pipiens	= CULPI
Ciona savigny	= CIOSA
Yarrowia lipolytica	= YARLI
Vitis vinifera	= VITVI
Equus caballus	= EQUCA
Entamoeba dispar	= ENTDI
Entamoeba histolytica	= ENTHI
Entamoeba invadens	= ENTIN
Entamoeba moshkovskii	= ENTMO
Glypta fumiferanae ichnovirus	= GLYFU
Helobdella robusta	= HELRO
Staphylococcus epidermidis	= STAEP
Meloidogyne incognita	= MELIN
Schmidtea mediterranea	= SCHME
Mycobacterium smegmatis	= MYCSM
Nassonia vitripennis	= NASVI
Phytopthora infestans	= PHYIN
Phytopthora sojae	= PHYSO
Trichinella spiralis	= TRISP
Penicillium chrysogenum	= PENCH
Medicago truncatula	= MEDTR
Deinococcus radiodurans	= DEIRA
Monodelphis domestica	= MONDO
Bos taurus	= BOSTA
Haemonchus contortus	= HAECO
Daphnia pulicara	= DPULI
Myotis lucifigus	= MYOLU

```
                                     *        20         *        4
gi|171691759_PODAN        : MAPNR---HLTTEERQRIRTLY-FDGHLSQPRIQETTG- :  34
gi|10764493_FUSOX         : MPRGK---ELTPSLRSRICELK-KQGY-SYSQIHKHFPY :  34
gi|14574243_CAEEL         : MPRGS---ALSDTERAQLDVMK-LLNV-SLHEMSRKIS- :  33
gi|157887063_Minos_DROHY : MVRGK---PISKEIRVLIRDYF-KSGK-TLTEISKQLN- :  33
gi|7641_Bari-1_DROME      : MPKTK---ELTVEARAGIVARF-KAGT-PAAKIAEIYQ- :  33
gi|157285978_SALSA        : MAETK---ELSKDVRDKIVDLH-KAGM-GYKTIAKQLG- :  33
gi|37678182_RANPI         : MPRPK---EIQEQLRKKVIEIY-QSGK-GYKAISKALG- :  33
gi|5579035_PLEPL          : -MKTK---ELTKQVRDKVVEKY-EAGL-GYKKISRALN- :  32
gi|118596560_ONCMY        : -MKNK---EHTRQVRDTVVKKF-KAGF-GYKKISQALN- :  32
gi|221124107_HYDMA        : MGK------ISLIERQKVIVLH-EEGY-SQRQISSKTG- :  30
gi|1196520_ANOAL          : MTREE----LSVSKRQDIIRLHGAQGK-SYTEIAMLTN- :  33
gi|4099803_ANOGA          : MGRGK---HCTPEERKDIQGLY-RENV-PIKTICKAFG- :  33
gi|4959263_HAECO          : MARHTGIRNLRQDQVDAIIRSF-HAGL-TSRQVSEIQG- :  36
gi|7495271_CAEEL          : MVKSVGCKNLSLDVKKAIVAGF-EQGI-PTKMLALQIQ- :  36
Tc1_DAPPU                 : MGNRK---ELSIEDRTKIIILR-RSTAMSMLEIGASVG- :  34
Tc2_DAPPU                 : ------------MEKTKIVTLR-QEGA-SLRKISLVLG- :  24

                            0         *        60         *
gi|171691759_PODAN        : YTKYQIRHAIRAPAAEVA-----PRSGRPRVITADQELE :  68
gi|10764493_FUSOX         : IPLGTIKTTCRREAQRGAENTTLPRSGAPRKLTEEQRDQ :  73
gi|14574243_CAEEL         : RSRHCIRVYLKDPVSYGTS----KRAPRRKALSVRDERN :  68
gi|157887063_Minos_DROHY : LPKSSVHGVIQIFKKNGNIENNIANRGRTSAITPRDKRQ :  72
gi|7641_Bari-1_DROME      : ISRRTVYYLIKKFDTVGTLKNK-KRSGRKPVLDQRQCRQ :  71
gi|157285978_SALSA        : EKVTTVGVIIRKWKKHKRTVNL-PRPGAPCKISPRGVAM :  71
gi|37678182_RANPI         : IQRTTVRAIIHKWRRHGTVVNL-PRSGRPPKITPRAQRR :  71
gi|5579035_PLEPL          : ISLSTIKSIIRKWKEYGTTANL-PRGGRPPKLKSRTRRK :  70
gi|118596560_ONCMY        : IPRSTVQAIILKWKEYQTTANL-PRPGRPSKLSAHTRRR :  70
gi|221124107_HYDMA        : YSKTAIREIIKKFRETGSLRNR-KKSGRPPKLTKDDNKY :  68
gi|1196520_ANOAL          : INRNTVARVIQRYKYEGRVSNL-PRKGRPSVCTDRMRRA :  71
gi|4099803_ANOGA          : RSRSFVDNAIRSEATG-------KSTGRPRKTTADVDAR :  65
gi|4959263_HAECO          : VTIRCVQRIWKKYKDTGSVEVK-KHPGAARTTSRLVDRN :  74
gi|7495271_CAEEL          : RSPSTIWKVIKKYQTEKSVALR-ISPGRPRVTTHRMDRN :  74
Tc1_DAPPU                 : CSVSCVKKNLDRYCETNSLEDR-PRSGRPSVMTEKDRHY :  72
Tc2_DAPPU                 : RSVNAVKQAINRFNETECIGKRERKTSSKLKISDASGRY :  63

                            80         *       100         *
gi|171691759_PODAN        : LIRYVCESKAHRRMSFLELSIALFNSLLNW--ITIRNAL : 105
gi|10764493_FUSOX         : IYDTVITDPHVTTRDLLDSVDNVIKK------RSLRYLL : 106
gi|14574243_CAEEL         : VIR-AASNSCKTARDIRNELQLSASK------RTILNVI : 100
gi|157887063_Minos_DROHY : LAKIVKADRRQSLRNLASKWSQTIGKTVKR--EWTRQQL : 109
gi|7641_Bari-1_DROME      : ILGVVAKNPSASPVKIALESKNTIGKQVSS--STIRRRL : 108
gi|157285978_SALSA        : IMRTVRNQPRTTWEDLVNDLN-AAGTIVTK--KTIGNTL : 107
gi|37678182_RANPI         : LIQEVTKDPTTTSKELQASLA-SVKVSVHA--STIRKRL : 107
gi|5579035_PLEPL          : LIREATRRPMVTLEELQRSTA-EVGESVHR--TTISRLL : 106
gi|118596560_ONCMY        : LIRDAAKRPMITLDELQRSTA-EVGDSVHR--TTISGVL : 106
gi|221124107_HYDMA        : LKTLSLRNRKKTSTELAKDINTATGKNVSS--SCIRRHL : 105
gi|1196520_ANOAL          : IKRLVDAEPEISAQSVAIVLNERHGIAISC--ETVRRYI : 108
gi|4099803_ANOGA          : IVEMIRADPFKTCTRIKQELGLQVSA------KTVSRRL :  98
gi|4959263_HAECO          : IVRLARNDPRLTAAEILREISTPEGSNLSL--STVQRRL : 111
gi|7495271_CAEEL          : ILRSAREDPHRTATDIQMIISSPNEPVPSK--RTVRRRL : 111
Tc1_DAPPU                 : VVLLAKRNRFKTLPVLHEEFNCGRKKEEKSLQNCYHKAL : 111
Tc2_DAPPU                 : LQLLSKRDRRKTLPLLTQEVSSAVGTPVSM--STVRRSL : 100
```

```
                               120        *        140        *
gi|171691759_PODAN        : YRHGFRRRVA-RKKPPISEANQQKRLAWAIEHKDWTLEQ : 143
gi|10764493_FUSOX         : REMNKRKWIQ-KKRVALTPLQARKRLDWAIRYQG---ID : 141
gi|14574243_CAEEL         : KRSGVIVRQKLRPAPLLSADHKLKRLEFAKNNMG---TN : 136
gi|157887063_Minos_DROHY : KSIGYGFYKA-KEKPLLTLRQKKKRLQWARERMSWTQRQ : 147
gi|7641_Bari-1_DROME      : KEADFKTYVV-RKTIEITPTNKTKRLRFALEYVKKPLDF : 146
gi|157285978_SALSA        : RREGLKSCSA-RKVPLLKKAHIHARLKFASEHLNDSEDN : 145
gi|37678182_RANPI         : GKNGLHGRVP-RRKPLLSKKNIKARLNFSTTHLDDPQDF : 145
gi|5579035_PLEPL          : HKSGLYGRVA-RRKPLLKGIHKKSRLEFARSHVGDTANM : 144
gi|118596560_ONCMY        : HKSGLYGRVA-RRKPFLKDIHKKCRLKFATSHLGDTPNM : 144
gi|221124107_HYDMA        : LKSGLRGCVA-IRKPLLRRGNREKRLKYAKQHKDWTQEM : 143
gi|1196520_ANOAL          : HKFGYKAYNR-RKKPQISPINRKRRLEFAKKYVNHPPEF : 146
gi|4099803_ANOGA          : HAAGFCARRP-RKVRKLQHHHVEARIRFAEEHLAASIFW : 136
gi|4959263_HAECO          : REAGLFGRRP-AKKPLISAKNRKARLDWAQAHKNWTVRQ : 149
gi|7495271_CAEEL          : QQAGLHGRKP-VKKPFISKKNRMARVAWAKAHLRWGRQE : 149
Tc1_DAPPU                 : AKNALNGRVA-AKKPLLKSMNIKKRLAFAKAHVDWSEKQ : 149
Tc2_DAPPU                 : QSFDMNGRVA-CKKPLLRKANIRKRLKFAQEHVNWTVEQ : 138

                                 160        *        180        *
gi|171691759_PODAN        : WRTILWSDETWV-VGGPHRKQYVTRRIDEEWDPTCIVEK : 181
gi|10764493_FUSOX         : WRRVKWSDECMVRRGQGMRPIWTFLSPREALRVQDVQEA : 180
gi|14574243_CAEEL         : WSKVVFSDEKKFNLDGPDGCRYYWRDLRKE---PMVFSR : 172
gi|157887063_Minos_DROHY : WDTIIFSDEAKFDVSVGDTRKRVIRKRSETYHKDCLKRT : 186
gi|7641_Bari-1_DROME      : WFNILWTDESAFQYQGSYSKHFMHLKNNQKHL--AAQPT : 183
gi|157285978_SALSA        : WVKVLLADETKMELFGINSTHRVWRRRNAAYDPKNTIPT : 184
gi|37678182_RANPI         : WDNILWTDETKVELFGRCVSKYIWRRRNTAFHKKNIIPT : 184
gi|5579035_PLEPL          : WKKVLWSDETKIELFGLNAKRYVWRKPNTAHHPEHTIPT : 183
gi|118596560_ONCMY        : WKKVLWSDETKIELFGNNAKRYVWRKSNTAHHHEHTIPT : 183
gi|221124107_HYDMA        : FNRVLYTDESKFEIFGTKRRQYVRRRIGEAYQPECLNPT : 182
gi|1196520_ANOAL          : WKKVLFTDESKFNIFGWDGTIKVWRPPGEGLNPKYTAKT : 185
gi|4099803_ANOGA          : WSKIIFSDESRINLDGSDGIKYVWRFPNQAYHPKNTIKT : 175
gi|4959263_HAECO          : WRKVIWSDESKFLLFGTDGIKFVRRPVGTRYHPSYQLPT : 188
gi|7495271_CAEEL          : WAKHIWSDESKFNLFGSDGNSWVRRPVGSRYSPKYQCPT : 188
Tc1_DAPPU                 : WSRVLFADESKFEFFGNKRRHYVRRFTNELLKNYCLTPT : 188
Tc2_DAPPU                 : WNRVLFTDETKVEVFGTHRRIFVRRKAGERYRNECLVPT : 177

                                   200        *        220        *
gi|171691759_PODAN        : HQRKGGW-MFWGCFYGSTKGPGIFWEKEWGSINKYSYRK : 219
gi|10764493_FUSOX         : RRLGAVRQMFWAAFGHRSRTPLVPLV---GNVNAIGIYE : 216
gi|14574243_CAEEL         : RNFGGGTVMVWGAFTEKKKLEIQFVS---SKMNSTDYQN : 208
gi|157887063_Minos_DROHY : TKFPAST-MVWGCMSAKGLGKLHFIE---GTVNAEKYIN : 221
gi|7641_Bari-1_DROME      : NRFGGGTVMFWGCLSYYGFGDLVPIE---GTLNQNGYLL : 219
gi|157285978_SALSA        : VKHGGGNIMLWGCFSAKGTGQLHCIK---GTMDGAMYRQ : 220
gi|37678182_RANPI         : VKYGGGSVMVWGCFAASGPGRLAVIK---GTMNSAVYQE : 220
gi|5579035_PLEPL          : VKHGGGSIMLWGCFSSAGTGKLVRIE---GKMDGAKYRE : 219
gi|118596560_ONCMY        : VKHGGGSIMVWACFSSAGTGKMVKID---GKMDGAKYRT : 219
gi|221124107_HYDMA        : IKHGGGSLQVWGCLSSSGVGDLIKIE---GPLTGERYVD : 218
gi|1196520_ANOAL          : VKHNGGGVLVWGCMAANGVGNLQVID---GIMDQYVYIN : 221
gi|4099803_ANOGA          : LSHGGGHVMVWGCFFWHGTGPLFRIN---GTLNSEGYRK : 211
gi|4959263_HAECO          : VKGGGDSVMVHGSFCGKGAGPLHRIE---GKMDAKMYLN : 224
gi|7495271_CAEEL          : VKHGGGSVMVWGCFTSTSMGPLRRIQ---SIMDRFQYEK : 224
Tc1_DAPPU                 : VKHGGGSVMVWGGICVRGVTRLKLIV---GKMDSSAYNS : 224
Tc2_DAPPU                 : VKFGGGSVMCWGAICAKGALPLHRIE---GIMTKQTYHQ : 213
```

```
                                   240         *       260         *
gi|171691759_PODAN       : NILPNV----------HKFINDTFEQHHLQLSFMQDGAP : 248
gi|10764493_FUSOX        : LYSF-I----------LPWFLQ------SGDIFMHDNAS : 238
gi|14574243_CAEEL        : VLELEL----------SKYLRHY-SR--KDFRFQQDNAT : 234
gi|157887063_Minos_DROHY : ILQDSL----------LPSIPKLSDC--GEFTFQQDGAS : 248
gi|7641_Bari-1_DROME     : ILNNHA----------FTSGNRLFPT--TEWILQQDNAP : 246
gi|157285978_SALSA       : ILGENL----------LPSARALKMG--HGCVFQHDNDP : 247
gi|37678182_RANPI        : ILKENV----------RPSVRVLKLK--RTWVLQQDNDP : 247
gi|5579035_PLEPL         : ILEENL----------MQSAKDLRLG--RRFIFQQDNDP : 246
gi|118596560_ONCMY       : ILEENL----------MESAKDLRLG--RRFVFQQDNDP : 246
gi|221124107_HYDMA       : ILRQHA----------IPSGMRL-IG--HNFILQQDNDP : 244
gi|1196520_ANOAL         : ILKQNL----------GPSLEKLGMS--QDYWFQQDNDP : 248
gi|4099803_ANOGA         : ILSREM----------LPYARQQ-FGDEEHYIFQHDNDS : 239
gi|4959263_HAECO         : IMETVI----------WPFVRST-AR--RGFIFQQDNDP : 250
gi|7495271_CAEEL         : ILETTM----------RPWALQN-VG--RGFVFQQDNDP : 250
Tc1_DAPPU                : ILQHNV----------LPDGVRL-LG--KGFVLQQDNDP : 250
Tc2_DAPPU                : ILIRKVTKKFLFIFCAVPGGIRL-LG--EGFVYQEDNDP : 249

                                    280         *       300         *
gi|171691759_PODAN       : GHRAAGTKKDLEN-----------------------RKI : 264
gi|10764493_FUSOX        : VHTARIVKALLEE-----------------------LGV : 254
gi|14574243_CAEEL        : IHVSNSTRDYFKL-----------------------KKI : 250
gi|157887063_Minos_DROHY : SHTAKRTKNWLQY-----------------------NQM : 264
gi|7641_Bari-1_DROME     : CHKGRIPTKFLND-----------------------LNL : 262
gi|157285978_SALSA       : KHTAKATKEWLKK-----------------------KHI : 263
gi|37678182_RANPI        : KHTSKSTTEWLKK-----------------------NKM : 263
gi|5579035_PLEPL         : KHTARATKEWFGL-----------------------KNV : 262
gi|118596560_ONCMY       : KHKAKSTMEWFKN-----------------------KHI : 262
gi|221124107_HYDMA       : KHCSRVAKNYLNE-------------------MAEEGIL : 264
gi|1196520_ANOAL         : KHTAFNSRLFLLY-----------------------NTP : 264
gi|4099803_ANOGA         : KHTSRTVKCYLAN-----------------------QDV : 255
gi|4959263_HAECO         : KHKSKLLTKWFRD-----------------------NNV : 266
gi|7495271_CAEEL         : KHTSLHVRSWFQR-----------------------RRV : 266
Tc1_DAPPU                : KHKEKRVMHYLGN---------------------KEKDG : 268
Tc2_DAPPU                : KHSSHLCRGYLERKEKLGRQMNSRLIIISKICFLSLGVL : 288

                                     320         *       340         *
gi|171691759_PODAN       : RVVDWPPFSPDLNPIESCWNWMKDYIEDKYGL--EEKPS : 301
gi|10764493_FUSOX        : DLMTWPPYSPDLNPIENLWALMKAEIYRLHPELTHTEDT : 293
gi|14574243_CAEEL        : NLLDWPARSPDLNPIENLWGILVRIVYAQNKTYPTVAS- : 288
gi|157887063_Minos_DROHY : EVLDWPSNSPDLSPIENIWWLMKNQLRNEPQR--NISD- : 300
gi|7641_Bari-1_DROME     : AVLPWPPQSPDLNIIENVWAFIKNQRTIDKNR--KREG- : 298
gi|157285978_SALSA       : KVLEWPSQSPDLNPIENLWRELKVRVAKRPPR--NLND- : 299
gi|37678182_RANPI        : KTLEWPSQSPDLNPIEMLWYDLKKAVHARKPS--NVTE- : 299
gi|5579035_PLEPL         : NVLKWPSQSPDLNPIENLWQDLKIAVHRRSPS--NLTE- : 298
gi|118596560_ONCMY       : QVLEWPSQSPDLNPIENLWKELKTAVHKCSPS--NLTE- : 298
gi|221124107_HYDMA       : EMMTWPPQSPDLNIIEHIWDYLDRKKVEHAPR--NAEE- : 300
gi|1196520_ANOAL         : HQLKSPPQSPDLNPIEHAWELLERKIRQTRIK--NRVD- : 300
gi|4099803_ANOGA         : QVLPWPALSPDLNPIENLWSTLKRQLKNQPAR--SADD- : 291
gi|4959263_HAECO         : PLLMWPSLSPDLNATENLWERLKHQVKGLRAR--NEHE- : 302
gi|7495271_CAEEL         : HLLDWPSQSPDLNPIEHLWEELERRLGGIRAS--NADA- : 302
Tc1_DAPPU                : IFFSFHICTKDMCT--------------------NLKN- : 286
Tc2_DAPPU                : KRMVWPPQSPDLNPIEQVWDFVKSRLEESDRV--TVRT- : 324
```

```
                                        360         *         380         *
gi|171691759_PODAN        : YAK---LKRYVEEAWQELPESYLQTLLDSMPSRCEAVIA : 337
gi|10764493_FUSOX         : VATQHALVLAAMEAWDNIEDRVLKNLCETMPNRVTAVIT : 332
gi|14574243_CAEEL         : ------LKQGILDAWKSIPDNQLKSLVRSMEDRLFEIIR : 321
gi|157887063_Minos_DROHY : ------LKIKLQEMWDSISQEHCKNLLSSMPKRVKCVMQ : 333
gi|7641_Bari-1_DROME      : ------AIIEIAEIWSKLTLEFAQTLVRSIPKRLQAVID : 331
gi|157285978_SALSA        : ------LEKICKEEWDKIPPEMCANLVANYKKCLTSVIA : 332
gi|37678182_RANPI         : ------LGQFCKDEWAKIPPGRCKSLIARYRKRLVAVVA : 332
gi|5579035_PLEPL          : ------LHLFCQEEWTNLSISRCAKLVETYPKRLAAVIA : 331
gi|118596560_ONCMY        : ------LELFCKEEWEKMSVSRCAKLIETHPKRLTAVIA : 331
gi|221124107_HYDMA        : ------CFQLLQKEWHNIPQDFITNLYESISRRISAVIK : 333
gi|1196520_ANOAL          : ------LENKLKEAWITISEDYTQNLVNSMPRRLAEVIK : 333
gi|4099803_ANOGA          : ------LWTRCKFMWERIDRSESRNLIGDMAKRCQEVIA : 324
gi|4959263_HAECO          : ------KFNQLKTAWENIPQEEIDKLIESMPCRCQAVID : 335
gi|7495271_CAEEL          : ------IFNQLENAWKAIPMSVIHKLIDSMPRRCQAVID : 335
Tc1_DAPPU                 : ------FFEHLQKCWHSITRKTLKKYLFFVKDRCLAVIA : 319
Tc2_DAPPU                 : ------IWMELEKAWKMITPELIQRYIGTMADRCRAVIL : 357


gi|171691759_PODAN        : ANGMHTKY : 345
gi|10764493_FUSOX         : AEGWYTKY : 340
gi|14574243_CAEEL         : TQGNPINY : 329
gi|157887063_Minos_DROHY : AKGDVTQF : 341
gi|7641_Bari-1_DROME      : AKGGVTKY : 339
gi|157285978_SALSA        : NKGFSTKY : 340
gi|37678182_RANPI         : AKGGPTSY : 340
gi|5579035_PLEPL          : AKGGSTKY : 339
gi|118596560_ONCMY        : AKGGATKY : 339
gi|221124107_HYDMA        : AKGGHSKY : 341
gi|1196520_ANOAL          : MKGYATRY : 341
gi|4099803_ANOGA          : NNGHQIDR : 332
gi|4959263_HAECO          : ARGHATKY : 343
gi|7495271_CAEEL          : ANGYATKY : 343
Tc1_DAPPU                 : AKGGHTRV : 327
Tc2_DAPPU                 : AKGGHTRY : 365
```

Supplemental Figure S2b. Alignment of Pogo family elements from multiple species, including three new families, Pogo_A1.1_Dappu, Pogo_A2.1_Dappu, and Pogo_A3.1_Dappu from *Daphnia pulex*.

Species abbreviations

Drosophila melanogaster = DROME
Drosophila biofasciata = DROBI
Drosophila helvetica = DROHE
Drosophila willistoni = DROWI
Drosophila hydei = DROHY
Drosophila yakuba = DROYA
Drosophila persimilis = DROPE
Drosophila pseudoobscura = DROPS
Drosophila buzzati = DROBU
Drosophila ananassae = DROAN
Mus musculus = MUSMU
Danio rerio = DANRE
Arabidopsis thaliana = ARATH
Trichomonas vaginalis = TRIVA
Fusarium oxysporum = FUSOX
Podospora anserina = PODAN
Caenorhabditis briggsae = CAEBR
Caenorhabditis elegans = CAEEL
Caenorhabditis remanei = CAERE
Bombyx mori = BOMMO
Rana pipiens = RANPI
Salmo salar salar = SALSA
Oncorhynchus mykiss = ONCMY
Pleuronectes platessa = PLEPL
Anopheles albimanus = ANOAL
Anopheles gambiae = ANOGA
Haemonchus contortus = HAECO
Saccoglossus kowalevskii = SACKO
Ixodes scapularis = IXOSC
Halorubrum lacusprofundi = HALLA
Hahella chejuensis = HAHCH
Marinobacter aquaeolei = MARAQ
Acidithiobacillus caldus = ACICA
Aspergillus niger = ASPNI
Talaromyces stipitatus = TALST
Culex quinquefasciatus = CULQU
Tribolium castaneum = TRICA
Oryza sativa = ORYSA
Aaedes aegypti = AAEAE
Ciona intestinalis = CIOIN
Trichuris muris = TRIMU
Schistosoma japonicum = SCHJA
Schistosoma mansoni = SCHMA

Antirrhinum majus = ANTMA
Bactrocera dorsalis = BACDO
Phakopsora pachyrhizi = PHAPA
Oryzias latipes = ORYLA
Zea mays = ZEAMA
Tolypocladium inflatum = TOLIN
Strongylocentrotus purpuratus = STRPU
Nematostella vectensis = NEMVE
Hydra magnipapillata = HYDMA
Glyptapanteles indiensis = GLYIN
Acyrthosiphon pisum = ACYPI
Branchiostoma floridae = BRAFL
Candida albicans = CANAL
Chelonus inanitus bracorvirus = CHEIN
Cucumis melo = CUCME
Culex pipiens = CULPI
Ciona savigny = CIOSA
Yarrowia lipolytica = YARLI
Vitis vinifera = VITVI
Equus caballus = EQUCA
Entamoeba dispar = ENTDI
Entamoeba histolytica = ENTHI
Entamoeba invadens = ENTIN
Entamoeba moshkovskii = ENTMO
Glypta fumiferanae ichnovirus = GLYFU
Helobdella robusta = HELRO
Staphylococcus epidermidis = STAEP
Meloidogyne incognita = MELIN
Schmidtea mediterranea = SCHME
Mycobacterium smegmatis = MYCSM
Nassonia vitripennis = NASVI
Phytopthora infestans = PHYIN
Phytopthora sojae = PHYSO
Trichinella spiralis = TRISP
Penicillium chrysogenum = PENCH
Medicago truncatula = MEDTR
Deinococcus radiodurans = DEIRA
Monodelphis domestica = MONDO
Bos taurus = BOSTA
Haemonchus contortus = HAECO
Daphnia pulicara = DPULI
Myotis lucifigus = MYOLU

```
                                          *        20          *
Pogo_A1.1_DAPPU               : SLNADLDDSIIGFVKEILSAVVSLFRC-PEQFRQGLYT :  37
Pogo_A2.1_DAPPU               : SRYPELEDAVLKWFKELRNPT---NKCKPLSLSRAHIQ :  35
Pogo_A3.1_DAPPU               : SENKELDDAVYDWFLEMRNPK---FRCKPLSISRAHIQ :  35
gi|241851667|_IXOSC           : -----VEEALVLWLKKARTKN--------LPVSGPLLL :  25
gi|126291201|Tigger_MONDO     : ALYDDIDKAVFAWFQEVHAKN--------ILVSGSVIR :  30
gi|2133672|_Pogo_DROME        : GAHDLVEEALYIWFGQQESKN--------VILDRHVIL :  30
gi|109658525|_HOMSA           : --YDDIDKAVFAWFQEIHAKN--------ILVTGSVIR :  28
gi|193685886|_ACYPI           : GKFDELEHILLEWFNQARTLN--------LPVNDNIVT :  30
gi|185050043|_BOMMO           : --HTNIEEALLKWFKYQRSNN--------VPINGPILQ :  28
gi|156139177|_SACKO           : SAVDDVEQALLNWFTSARSMN--------VPISGPILQ :  30

                                40         *         60         *
Pogo_A1.1_DAPPU               : NQKRSGNSKRPMDGLEIGDEDMELARVFVSLEKLVMIS :  75
Pogo_A2.1_DAPPU               : ARAAHEAKLRGILNFKASDGWFRNWRNRCLIGPSLRLF :  73
Pogo_A3.1_DAPPU               : ARALREAELRGISGFSASDGWFRNWRRRYEIGVSIRLY :  73
gi|241851667|_IXOSC           : EKARFFATQLHHDDFVCSNGWLSRFRARYNI-ATRVIS :  62
gi|126291201|Tigger_MONDO     : KKALNLANMLGYDNFQASVGWLNRFRDRHGI-ALKAIC :  67
gi|2133672|_Pogo_DROME        : AKAKEFCQKFN-DAFEPDASWLWRWRKRHNI-KYGKIH :  66
gi|109658525|_HOMSA           : KKALNLANMLGYDNFQASVGWLNRFRDRHGI-ALKAVC :  65
gi|193685886|_ACYPI           : EKAHEIAKRLNIDEFSGSGGWIDRFKKRHGI-VYRQIC :  67
gi|185050043|_BOMMO           : EKANHFAKCFG-EDFVCSSSWIQRFRARHGI-VGGKMS :  64
gi|156139177|_SACKO           : AKAEDLGQRMNHSEFKASNGWLERFKSRHGI-TFKSVC :  67

                                  80         *        100          *
Pogo_A1.1_DAPPU               : RKK-------------------VIRDKLRKYNPKNIFN :  94
Pogo_A2.1_DAPPU               : GEA-------------------AIRNKLQRFNASNIFN :  92
Pogo_A3.1_DAPPU               : GEA--------------------LRVQLANYDLKNIFN :  91
gi|241851667|_IXOSC           : GKGA-----AADTDGAEEWQNGQLQQILTDYAPEDIFN :  95
gi|126291201|Tigger_MONDO     : RED-------------------EIIKLIADYSPDDIFN :  86
gi|2133672|_Pogo_DROME        : GET-------------------ILPGLLKGYNPEDIFN :  85
gi|109658525|_HOMSA           : REDSDRLMNGLGIDKINEWHAGEIIKLIADYSPDDIFN : 103
gi|193685886|_ACYPI           : GEAE-----SVNDADIAAWSENILPNILKEYSSNDIFN : 100
gi|185050043|_BOMMO           : REAA-----SVDKDTVEEWKTQKRPTLFEGYGPDEVFN :  97
gi|156139177|_SACKO           : GEAA-----SVSDDMVDDWVTLTLPGLIDGYAPRDIFN : 100

                                   120         *        140          *
Pogo_A1.1_DAPPU               : MDETGLLFRALPTSTYVSLEEGSRKKIRGTKVLRAKDR : 132
Pogo_A2.1_DAPPU               : MDETGLFFRALPTRTYVCREEGQRKTVRGTKALRAKDR : 130
Pogo_A3.1_DAPPU               : MDETGLFYRAMPARTYLAYNES-RKTVRGTKSLKAKDR : 128
gi|241851667|_IXOSC           : LDESALFFRLLPNRTLVSK----GQACTGGK--HAKDR : 127
gi|126291201|Tigger_MONDO     : ADETGMFFQLLPQHTLTAK----GDCCRGGK--KARQR : 118
gi|2133672|_Pogo_DROME        : ADETALFYKAMPNATFFTC----GKQLNGQK--SQRVR : 117
gi|109658525|_HOMSA           : ADETGVFFQLLPQHTLAAK----GDHCRGGK--KAKQR : 135
gi|193685886|_ACYPI           : ADEFGLFFKLMPDKSLVFK----REKCHGGK--LSKER : 132
gi|185050043|_BOMMO           : AAETGLFYNMTPDKTLKFK----GENCSGGK--MSKTR : 129
gi|156139177|_SACKO           : ADETGLFFRLMPDKTLSFK----NDVCSGGK--TSKER : 132
```

```
                                   160        *         180         *
Pogo_A1.1_DAPPU             : VTLVLCVNATG-TCKVAPLIVGTSKNPHCFRDSP-CP- : 167
Pogo_A2.1_DAPPU             : LTLVLCANATG-TCKIDPLLVGSAKNPHCFRDQQ-SP- : 165
Pogo_A3.1_DAPPU             : VTLVLCVNVDGGSCKIEPLIVGTAAKPHCFRDSP-CP- : 164
gi|241851667|_IXOSC         : ISVAFIVNMTG-SEKPPLLVIGKSEKPRCFKGGR-LPS : 163
gi|126291201|Tigger_MONDO   : LTALFCCNASG-TEKMKPLIVGKSATPRCFKNVHSLP- : 154
gi|2133672|_Pogo_DROME      : LTLLFICNATG-TYK-KTFVIGRSKSPRCFKNAN-VP- : 151
gi|109658525|_HOMSA         : LTALFCCNASG-TEKMRPLIVGRSASPHCLKNIHSLP- : 171
gi|193685886|_ACYPI         : LSVLACTNATG-SQKLRLLVIGKSKAPRCFKNVRTFP- : 168
gi|185050043|_BOMMO         : LTIMVAANMTG-SCKRKLLVIGESKKPKCFKNTHSLP- : 165
gi|156139177|_SACKO         : LTVMLCANMDG-SEKLKPLVIGKSKNPRCFKNVKSLP- : 168

                                     200        *         220
Pogo_A1.1_DAPPU             : -IPYVNQRNAWVDREVYKGWWLDIFLPAIRKFTKEPVA : 204
Pogo_A2.1_DAPPU             : -VPYVNQKNAWVDREIYRGWWNNIFLPAIRKHTKEPVA : 202
Pogo_A3.1_DAPPU             : -VPYIHQKNAWVDSAIYRHWWGNIFLPAVRAWTDEPVA : 201
gi|241851667|_IXOSC         : GVLYRSNTKAWMTAKIFEEYVRL--LDRRFAAKKRNVI : 199
gi|126291201|Tigger_MONDO   : -CDYRANQRAWMTRDLFNEWLMK--VDAKMKQAERRIL : 189
gi|2133672|_Pogo_DROME      : -IPYYANKKAWMTKDLWRKIMTG--FDEEMKKQNRKIL : 186
gi|109658525|_HOMSA         : -CDYRANQWAWMTRDLFNEWLMQ--VDARMKRAERRIL : 206
gi|193685886|_ACYPI         : -CDYVSQNRAWMTGDIFINWIKQ--LDLSFKKQNRNIL : 203
gi|185050043|_BOMMO         : -VTYENNVQSWMTSDIFERWLRN--WDAELKGNKQKVL : 200
gi|156139177|_SACKO         : -VDYNANKKAWMVSDLFIEWLHK--LDKKYKRQKRKIL : 203

                               *        240        *         260
Pogo_A1.1_DAPPU             : LIMDNCSGHDPTLTDPTGQVEIIFLP----------PN : 232
Pogo_A2.1_DAPPU             : LLMDNCSGHDPSCADPTGQVEIIFFP----------PN : 230
Pogo_A3.1_DAPPU             : LVMDNFSGHDVNCVDPTGQVKLHFSLFRHSVLFLKHPN : 239
gi|241851667|_IXOSC         : VVLDNASAH--VQVENLSAIKLVFLA----------PI : 225
gi|126291201|Tigger_MONDO   : MLIDNCSAH--NMLPRLERIQVGYLP----------SN : 215
gi|2133672|_Pogo_DROME      : LFIDNATSH--TTVKDFENIKLCFMP----------PN : 212
gi|109658525|_HOMSA         : LLIDNCSAH--NMLPHLERIQVGYLP----------SN : 232
gi|193685886|_ACYPI         : LFVDNCPAH--PTTIPLENIKLVFLP----------PN : 229
gi|185050043|_BOMMO         : LLVDNCPAH--PAVTNLKCIKLVFLP----------PN : 226
gi|156139177|_SACKO         : MFVDNCPAH--PAVKNLKAIKLVFLP----------PN : 229

                                 *        280        *         300
Pogo_A1.1_DAPPU             : CTSVYQPLDQGIISTLKTLYKSEMLSEFVNAYDN : 266
Pogo_A2.1_DAPPU             : CTSVFQPLDQGIITTLKTLYKREMLSSFAAAYDK : 264
Pogo_A3.1_DAPPU             : STSVHQPLDQGIISAVKINYKKLMLSKFIEAYED : 273
gi|241851667|_IXOSC         : TTALAQPLDQGIIRAVKQTYRKNLLRRMLLSMEN : 259
gi|126291201|Tigger_MONDO   : CTAVLQPLNLGVIQTMKVLYRSRLLKQIVLNLNN : 249
gi|2133672|_Pogo_DROME      : ATALLQPLDQGIIHSFKLEYRRILVKQQLIAVNC : 246
gi|109658525|_HOMSA         : CTAVLQPLNLGIIHTMKVLYRSHLLKQILLKLNS : 266
gi|193685886|_ACYPI         : ATAKLQPLDQGIIKVLKQKYRKKLVLRYLKEMES : 263
gi|185050043|_BOMMO         : VTSVLQPMEQGVIRCLKSHYRRLQVLKLIQNLYS : 260
gi|156139177|_SACKO         : TTSKLQPMDQ-VIRNFKCFYRQRVVQRMLVNLDS : 262
```

Supplemental Figure S2c. Alignment of Ant family elements from multiple species, including one new family, Ant_A1.1_Dappu, from *Daphnia pulex*.

Species abbreviations

Drosophila melanogaster	= DROME
Drosophila biofasciata	= DROBI
Drosophila helvetica	= DROHE
Drosophila willistoni	= DROWI
Drosophila hydei	= DROHY
Drosophila yakuba	= DROYA
Drosophila persimilis	= DROPE
Drosophila pseudoobscura	= DROPS
Drosophila buzzati	= DROBU
Drosophila ananassae	= DROAN
Mus musculus	= MUSMU
Danio rerio	= DANRE
Arabidopsis thaliana	= ARATH
Trichomonas vaginalis	= TRIVA
Fusarium oxysporum	= FUSOX
Podospora anserina	= PODAN
Caenorhabditis briggsae	= CAEBR
Caenorhabditis elegans	= CAEEL
Caenorhabditis remanei	= CAERE
Bombyx mori	= BOMMO
Rana pipiens	= RANPI
Salmo salar salar	= SALSA
Oncorhynchus mykiss	= ONCMY
Pleuronectes platessa	= PLEPL
Anopheles albimanus	= ANOAL
Anopheles gambiae	= ANOGA
Haemonchus contortus	= HAECO
Saccoglossus kowalevskii	= SACKO
Ixodes scapularis	= IXOSC
Halorubrum lacusprofundi	= HALLA
Hahella chejuensis	= HAHCH
Marinobacter aquaeolei	= MARAQ
Acidithiobacillus caldus	= ACICA
Aspergillus niger	= ASPNI
Talaromyces stipitatus	= TALST
Culex quinquefasciatus	= CULQU
Tribolium castaneum	= TRICA
Oryza sativa	= ORYSA
Aaedes aegypti	= AAEAE
Ciona intestinalis	= CIOIN
Trichuris muris	= TRIMU
Schistosoma japonicum	= SCHJA
Schistosoma mansoni	= SCHMA
Antirrhinum majus	= ANTMA
Bactrocera dorsalis	= BACDO
Phakopsora pachyrhizi	= PHAPA
Oryzias latipes	= ORYLA
Zea mays	= ZEAMA
Tolypocladium inflatum	= TOLIN
Strongylocentrotus purpuratus	= STRPU
Nematostella vectensis	= NEMVE
Hydra magnipapillata	= HYDMA
Glyptapanteles indiensis	= GLYIN
Acyrthosiphon pisum	= ACYPI
Branchiostoma floridae	= BRAFL
Candida albicans	= CANAL
Chelonus inanitus bracorvirus	= CHEIN
Cucumis melo	= CUCME
Culex pipiens	= CULPI
Ciona savigny	= CIOSA
Yarrowia lipolytica	= YARLI
Vitis vinifera	= VITVI
Equus caballus	= EQUCA
Entamoeba dispar	= ENTDI
Entamoeba histolytica	= ENTHI
Entamoeba invadens	= ENTIN
Entamoeba moshkovskii	= ENTMO
Glypta fumiferanae ichnovirus	= GLYFU
Helobdella robusta	= HELRO
Staphylococcus epidermidis	= STAEP
Meloidogyne incognita	= MELIN
Schmidtea mediterranea	= SCHME
Mycobacterium smegmatis	= MYCSM
Nassonia vitripennis	= NASVI
Phytopthora infestans	= PHYIN
Phytopthora sojae	= PHYSO
Trichinella spiralis	= TRISP
Penicillium chrysogenum	= PENCH
Medicago truncatula	= MEDTR
Deinococcus radiodurans	= DEIRA
Monodelphis domestica	= MONDO
Bos taurus	= BOSTA
Haemonchus contortus	= HAECO
Daphnia pulicara	= DPULI
Myotis lucifigus	= MYOLU

```
                                           *         20          *         40
gi|74676245|Ant1_ASPNI : DESGCDRRIGFRRTG--WSP--LGLAPIQVAKFHRDQRYQI :  37
gi|1196520|_ANOAL      : DESKFNI-FGWDGTIKVWRPPGEGLNPKYTAKTVKHNGGGV :  40
gi|170040156|_CULQU    : DEVSFDNRDMLRTKG--YGV--VGQKVIFRGEFCRKPRASM :  37
gi|242761252|_TALST    : DESGCDKRAGFRRTG--WSP--LGVAPAQVTKFHRDQRYQI :  37
gi|120553347|_MARAQ    : DEASIRSDYHSGTT---WAP--KGETPIIRNTGSRF-SINL :  35
gi|83648550|_HAHCH     : DETGLRSDAQHGRG---YAP--QGKTPVIRLNARRE-SVNM :  35
gi|255021838|_ACICA    : DETAVKEDAHWVRG---YAP--KGQTPILEHPA-RWTTLSM :  35
Ant_A1.1_DAPPU         : DEAGIQMNSNLRRT---WAP--RGKTPILKQVTRSYRKISA :  36

                                          *         60          *         80
gi|74676245|Ant1_ASPNI : LPAY--SQDG---IFFSRIFQG-STDASVFEDFIEE---LL :  69
gi|1196520|_ANOAL      : LVWGCMAANG---VGNLQVIDG-IMDQYVYINILKQ--NLG :  75
gi|170040156|_CULQU    : LCFL--GSGG---ILDSFWTEG-TFTRLKFFDCCRDFALKN :  72
gi|242761252|_TALST    : LPAY--AQDG---VIMYYVFKG-STDASFFENFIEE---LL :  69
gi|120553347|_MARAQ    : ISAI--SPRG---ELRFKTIQG-TMNTDAFLGFLKA---LV :  67
gi|83648550|_HAHCH     : ISAI--SNQG---KVRFQIYDG-TMDADRLTGFMKR---LI :  67
gi|255021838|_ACICA    : ISAI--SPRG---EIAFEIVEG-SIHAERFIAFLEK---LI :  67
Ant_A1.1_DAPPU         : MGAIAVTPKGRRSRLFFRLLENKNFNTEACICFIEQ---LK :  74

                                         *        100          *        120
gi|74676245|Ant1_ASPNI : QHCGRWPEPKSVIV-MDNASFHHSEKIEELCSQAG--VKIV : 107
gi|1196520|_ANOAL      : PSLEKLGMSQDYWFQQDNDPKHTAFNSRLFLLYNT--PHQL : 114
gi|170040156|_CULQU    : KKVQRYPGFHSVWI-MDGAKIHCDRNIIMYLRSIG--ILPI : 110
gi|242761252|_TALST    : HHCGKWPEPKSVIV-MDNASFHHSKNIETMCSKAG--VKLV : 107
gi|120553347|_MARAQ    : QDADK----PVFLI-LDNHPVHHARRVREYVESLDGKLKLF : 103
gi|83648550|_HAHCH     : KDARR----KVFLI-LDNLRVHHSKVVKAWLEENRDHIEVF : 103
gi|255021838|_ACICA    : TGAPQ----KVFLV-VDNLRVHHAKVVSAWLADKQDRIELV : 103
Ant_A1.1_DAPPU         : QNIRG----QIVLV-WDRLLAHRSKRMAAYLSKQR-RVKLV : 109

                                      *
gi|74676245|Ant1_ASPNI : YLPPYSPDLNPIE : 120
gi|1196520|_ANOAL      : KSPPQSPDLNPIE : 127
gi|170040156|_CULQU    : FLPAYCPFFNPLE : 123
gi|242761252|_TALST    : YLPPYSPDLNPIE : 120
gi|120553347|_MARAQ    : FLPPYSP------ : 110
gi|83648550|_HAHCH     : YLPAYSPELNPDE : 116
gi|255021838|_ACICA    : FLPPYAPESNPDE : 116
Ant_A1.1_DAPPU         : FLPAYAP------ : 116
```

Supplemental Figure S2d. Alignment of hAT family elements from multiple species, including four new families, hAT_A1.1_Dappu, hAT_A3.1_Dappu, hAT_A4.1_Dappu, and hAT_A5.1_Dappu from *Daphnia pulex*.

Species abbreviations

Drosophila melanogaster	= DROME	Antirrhinum majus	= ANTMA
Drosophila biofasciata	= DROBI	Bactrocera dorsalis	= BACDO
Drosophila helvetica	= DROHE	Phakopsora pachyrhizi	= PHAPA
Drosophila willistoni	= DROWI	Oryzias latipes	= ORYLA
Drosophila hydei	= DROHY	Zea mays	= ZEAMA
Drosophila yakuba	= DROYA	Tolypocladium inflatum	= TOLIN
Drosophila persimilis	= DROPE	Strongylocentrotus purpuratus	= STRPU
Drosophila pseudoobscura	= DROPS	Nematostella vectensis	= NEMVE
Drosophila buzzati	= DROBU	Hydra magnipapillata	= HYDMA
Drosophila ananassae	= DROAN	Glyptapanteles indiensis	= GLYIN
Mus musculus	= MUSMU	Acyrthosiphon pisum	= ACYPI
Danio rerio	= DANRE	Branchiostoma floridae	= BRAFL
Arabidopsis thaliana	= ARATH	Candida albicans	= CANAL
Trichomonas vaginalis	= TRIVA	Chelonus inanitus bracorvirus	= CHEIN
Fusarium oxysporum	= FUSOX	Cucumis melo	= CUCME
Podospora anserina	= PODAN	Culex pipiens	= CULPI
Caenorhabditis briggsae	= CAEBR	Ciona savigny	= CIOSA
Caenorhabditis elegans	= CAEEL	Yarrowia lipolytica	= YARLI
Caenorhabditis remanei	= CAERE	Vitis vinifera	= VITVI
Bombyx mori	= BOMMO	Equus caballus	= EQUCA
Rana pipiens	= RANPI	Entamoeba dispar	= ENTDI
Salmo salar salar	= SALSA	Entamoeba histolytica	= ENTHI
Oncorhynchus mykiss	= ONCMY	Entamoeba invadens	= ENTIN
Pleuronectes platessa	= PLEPL	Entamoeba moshkovskii	= ENTMO
Anopheles albimanus	= ANOAL	Glypta fumiferanae ichnovirus	= GLYFU
Anopheles gambiae	= ANOGA	Helobdella robusta	= HELRO
Haemonchus contortus	= HAECO	Staphylococcus epidermidis	= STAEP
Saccoglossus kowalevskii	= SACKO	Meloidogyne incognita	= MELIN
Ixodes scapularis	= IXOSC	Schmidtea mediterranea	= SCHME
Halorubrum lacusprofundi	= HALLA	Mycobacterium smegmatis	= MYCSM
Hahella chejuensis	= HAHCH	Nassonia vitripennis	= NASVI
Marinobacter aquaeolei	= MARAQ	Phytopthora infestans	= PHYIN
Acidithiobacillus caldus	= ACICA	Phytopthora sojae	= PHYSO
Aspergillus niger	= ASPNI	Trichinella spiralis	= TRISP
Talaromyces stipitatus	= TALST	Penicillium chrysogenum	= PENCH
Culex quinquefasciatus	= CULQU	Medicago truncatula	= MEDTR
Tribolium castaneum	= TRICA	Deinococcus radiodurans	= DEIRA
Oryza sativa	= ORYSA	Monodelphis domestica	= MONDO
Aaedes aegypti	= AAEAE	Bos taurus	= BOSTA
Ciona intestinalis	= CIOIN	Haemonchus contortus	= HAECO
Trichuris muris	= TRIMU	Daphnia pulicara	= DPULI
Schistosoma japonicum	= SCHJA	Myotis lucifigus	= MYOLU
Schistosoma mansoni	= SCHMA		

```
                                         *        20        *       4
gi|6468207|Tol2_ORYLA     : SLKPTTHEASKELDGY-LACVSDTRESLLTFPAICSLSI : 38
gi|41018380|HOMSA         : ELSNFKSQK--VLGLN-EDPLKWWSDRLALFPLLPKVLQ : 36
gi|1542944|restless_TOLIN : EYERYIQTFTHADDKYQFRPLSWWQEHEMEYPNLCRMAT : 39
gi|19698550|hopper_BACDO  : SLEQYLRQD--FVERH-QNPLNYWDSKKATFPELYELSN : 36
Daphnia_hATA1.1/1-70      : EVKLYLSEP--TICKT-SDPLLYWKEKKEAFPCLYVMAM : 36
Daphnia_hatA3.1/1-70      : EVDNYLSET--ALGHQ-LDPLEWWKERRDRYPRLVVLVR : 36
gi|112702965|DANRE        : -IEMYRKEP--SISLT-ACPLKWWKENAQRYPLLSTLAM : 35
gi|73536305|MUSDO         : EFEFYRKEIV-ILSED-FKVXEWWNLNSKKYPKLSKLAL : 37
gi|113193577|hobo_DROME   : EIERYIRQRV-PLSQN-FEVIEWWKNNANLYPQLSKLAL : 37
gi|66730716|hAT1.1_PHAPA  : EINQYLSSE--REDKC-VQPLLWWKEKSGLFPSLARMAK : 36
gi|42600942|Herves_ANOGA  : ELHQYLSVE--NIDLE-NDPLLWWKEHQVLYPSLYTLAM : 36
gi|75194110|ARATH         : ELDQYLEESLIPRSQD-FEVLGWWSLNRTKYPTLSKMAA : 38
gi|13345976|Ac_ZEAMA      : ELDKYMSEPLLKHSGQ-FDILSWWRGRVAEYPILTQIAR : 38
gi|75220682|ANTMA         : EIHLFVQKPPQKFDKD-FDILKWWRQNESLTPVLARIAR : 38
hAT_A1.1_DAPPU            : EVKLYLSEP--TICKT-SDPLLYWKEKKEAFPCLYVMAM : 36
hAT_A3.1_DAPPU            : EVDNYLSET--ALGHQ-LDPLEWWKERRDRYPRLVVLVR : 36
hat_A4.1_DAPPU            : EV-----EKPEPLNCDPTAVYSYWANRSSRWPRLGQMAR : 34
hat_A5.1_DAPPU            : ----LASQDALECLKP-NIEKVYWKAHMTCYPRLSCMAR : 34

                            0          *        60          *
gi|6468207|Tol2_ORYLA     : KTNTPLPASAACERLFSTAGLLFSPKRARLDTNNFENQL : 77
gi|41018380|HOMSA         : KYWCVTATRVAPERLFGSAANVVSAKRNRLAPAHVDEQV : 75
gi|1542944|restless_TOLIN : DLLSIPTMSAETERSFSSAGKMVSPLRTRLDRHTIGMAQ : 78
gi|19698550|hopper_BACDO  : KYLCIPATSVPSERVFSKAGQIINDRRNRLKGEK----- : 70
Daphnia_hATA1.1/1-70      : KHLSVCATSTPSERAFSGGRLVCSHLRGSLSSEK----- : 70
Daphnia_hatA3.1/1-70      : RYLCIIMNSVPCERIFSKMGLVVTDRRTNLTAEK----- : 70
gi|112702965|DANRE        : SYLSVPATSVPNERVFSVAGDIVNAQRAQLLPDNVDMLI : 74
gi|73536305|MUSDO         : SLLSIPASSAASERTFSLAGNIITEKRNRIGQQTVDSLL : 76
gi|113193577|hobo_DROME   : KLLSIPASSAAAERVFSLAGNIITEKRNRLCPKSVDSLL : 76
gi|66730716|hAT1.1_PHAPA  : MYLAIPATSVPSERLFSTGRRMITDYRGRLTEGQIE--- : 72
gi|42600942|Herves_ANOGA  : STLCIPGTSVPCERLFSKAGQIYSEKRSRLAPKK----- : 70
gi|75194110|ARATH         : DVLSVPFCTVSPDSVFDTEVKKMDNYRSSLRHVTLEALF : 77
gi|13345976|Ac_ZEAMA      : DVLAIQVSTVASESAFSAGGRVVDPYRNRLGSEIVEALI : 77
gi|75220682|ANTMA         : DLLSSQMSTVASERAFSAGHRVLTDARNRLKPGSVKFCM : 77
hAT_A1.1_DAPPU            : KHLSVCATSTPSERAFSGGRLVCSHLRGSLSSEKLTALM : 75
hAT_A3.1_DAPPU            : RYLCIIMNSVPCERIFSKMGLVVTDRRTNLTAE------ : 69
hat_A4.1_DAPPU            : DLLSIPATSAASERAFSVGKDVFGISRMSLKPETVEALI : 73
hat_A5.1_DAPPU            : DYLAITATSASSERVFSTGKDLLGICRLCLKPTTMEACM : 73
```

```
                             80
gi|6468207|Tol2_ORYLA      : LLKLN : 82
gi|41018380|HOMSA          : FLYEN : 80
gi|1542944|restless_TOLIN  : GMRSW : 83
gi|19698550|hopper_BACDO   : ----- :  -
Daphnia_hATA1.1/1-70       : ----- :  -
Daphnia_hatA3.1/1-70       : ----- :  -
gi|112702965|DANRE         : FLKKN : 79
gi|73536305|MUSDO          : FLNSF : 81
gi|113193577|hobo_DROME    : FLHSY : 81
gi|66730716|hAT1.1_PHAPA   : ----- :  -
gi|42600942|Herves_ANOGA   : ----- :  -
gi|75194110|ARATH          : CAKDW : 82
gi|13345976|Ac_ZEAMA       : CTKDW : 82
gi|75220682|ANTMA          : IWKDM : 82
hAT_A1.1_DAPPU             : CLKSW : 80
hAT_A3.1_DAPPU             : ----- :  -
hat_A4.1_DAPPU             : CLRSW : 78
hat_A5.1_DAPPU             : CLRSW : 78
```

Supplemental Figure S2e. Alignment of P family elements from multiple species, including one new family, Pelement_A1.1_Dappu, from *Daphnia pulex*.

Species abbreviations

Drosophila melanogaster = DROME
Drosophila biofasciata = DROBI
Drosophila helvetica = DROHE
Drosophila willistoni = DROWI
Drosophila hydei = DROHY
Drosophila yakuba = DROYA
Drosophila persimilis = DROPE
Drosophila pseudoobscura = DROPS
Drosophila buzzati = DROBU
Drosophila ananassae = DROAN
Mus musculus = MUSMU
Danio rerio = DANRE
Arabidopsis thaliana = ARATH
Trichomonas vaginalis = TRIVA
Fusarium oxysporum = FUSOX
Podospora anserina = PODAN
Caenorhabditis briggsae = CAEBR
Caenorhabditis elegans = CAEEL
Caenorhabditis remanei = CAERE
Bombyx mori = BOMMO
Rana pipiens = RANPI
Salmo salar salar = SALSA
Oncorhynchus mykiss = ONCMY
Pleuronectes platessa = PLEPL
Anopheles albimanus = ANOAL
Anopheles gambiae = ANOGA
Haemonchus contortus = HAECO
Saccoglossus kowalevskii = SACKO
Ixodes scapularis = IXOSC
Halorubrum lacusprofundi = HALLA
Hahella chejuensis = HAHCH
Marinobacter aquaeolei = MARAQ
Acidithiobacillus caldus = ACICA
Aspergillus niger = ASPNI
Talaromyces stipitatus = TALST
Culex quinquefasciatus = CULQU
Tribolium castaneum = TRICA
Oryza sativa = ORYSA
Aaedes aegypti = AAEAE
Ciona intestinalis = CIOIN
Trichuris muris = TRIMU
Schistosoma japonicum = SCHJA
Schistosoma mansoni = SCHMA

Antirrhinum majus = ANTMA
Bactrocera dorsalis = BACDO
Phakopsora pachyrhizi = PHAPA
Oryzias latipes = ORYLA
Zea mays = ZEAMA
Tolypocladium inflatum = TOLIN
Strongylocentrotus purpuratus = STRPU
Nematostella vectensis = NEMVE
Hydra magnipapillata = HYDMA
Glyptapanteles indiensis = GLYIN
Acyrthosiphon pisum = ACYPI
Branchiostoma floridae = BRAFL
Candida albicans = CANAL
Chelonus inanitus bracorvirus = CHEIN
Cucumis melo = CUCME
Culex pipiens = CULPI
Ciona savigny = CIOSA
Yarrowia lipolytica = YARLI
Vitis vinifera = VITVI
Equus caballus = EQUCA
Entamoeba dispar = ENTDI
Entamoeba histolytica = ENTHI
Entamoeba invadens = ENTIN
Entamoeba moshkovskii = ENTMO
Glypta fumiferanae ichnovirus = GLYFU
Helobdella robusta = HELRO
Staphylococcus epidermidis = STAEP
Meloidogyne incognita = MELIN
Schmidtea mediterranea = SCHME
Mycobacterium smegmatis = MYCSM
Nassonia vitripennis = NASVI
Phytopthora infestans = PHYIN
Phytopthora sojae = PHYSO
Trichinella spiralis = TRISP
Penicillium chrysogenum = PENCH
Medicago truncatula = MEDTR
Deinococcus radiodurans = DEIRA
Monodelphis domestica = MONDO
Bos taurus = BOSTA
Haemonchus contortus = HAECO
Daphnia pulicara = DPULI
Myotis lucifigus = MYOLU

```
                               *        20        *         40
gi|7155|DROBI       : -----------IVRGLKKSWKQPIFFDFSTRMDADT-LNN---- :  28
gi|12830679|DROHE   : -----------IVRGLKKSWKQPIFFDFSTRMDADT-LNN---- :  28
gi|221131281|HYDMA  : -----NHVLVVMVRGIAIKWKQPIAYFYSNNSVQSVKLSQIILE :  39
gi|51102853|DROWI   : -----------IVRGLKKSWKQPVFFDFNTRMDPDT-LNN---- :  28
gi|213517460|GLYIN  : --A--SKVLAIVVKGLCKRWKQVIGTLLTGPSVNSDKQWQCIKN :  40
gi|83356293|ANOGA   : -----------MARALFKNWKQPIYIGFDKKMTK-----EILMN :  28
gi198413217|CIOIN   : ---VATHILCFMVKGITQKWKQVIAYYYTGDSVSGEDLWNTCKS :  41
Pelement_A1.1_DAPPU : LLA--NRLLGFLMTGLSTSYKIPVAFFFVRKLTAVQ-LYKLTCY :  41

                           *        60        *         80
gi|7155|DROBI       : IIRKLHTKGYPVVAIVSDLGSGNQRLWSELG-----VSESK-TW :  66
gi|12830679|DROHE   : IIRKLHTKGYPVVAIVSDLGSGNQKLWSELG-----VSESK-SW :  66
gi|221131281|HYDMA  : AIDKLTSIGLHVRAVVCNQSTTNISALKLLR-----FLNTR-PY :  77
gi|51102853|DROWI   : ILRKLHRKGYLVVAIVSDLGTGNQKLWTELG-----ISESK-TW :  66
gi|213517460|GLYIN  : LIQQLEEIGLDVRGVSSDMGPNNVGMWSLLGIHATRGAKVS-CS :  83
gi|83356293|ANOGA   : IIIKLDEKKINVAGIVSDNCSSNISCWRDLG-----AHDYTKPY :  67
gi198413217|CIOIN   : IVIELGKRSFHVKSVVSDMGASNQAMWREAG------IQSTKLA :  79
Pelement_A1.1_DAPPU : VIREIELEGFSVERVVTDNASTNVKMFKYFG-----NGEVV-PF :  79

                        *        100        *         120        *
gi|7155|DROBI       : F----SHPTDEHLKIFVFSDTPHLIKLVRNHYVDSG-------- :  98
gi|12830679|DROHE   : F----SHPTDEHLKISVFPDTPHLIKLVRNHYVDSG-------- :  98
gi|221131281|HYDMA  : I----S-PSSIQTNVYIIFDPPHLIKSLRNNLIKHD-------- : 108
gi|51102853|DROWI   : F----SHPADDHLKIFVFSDTPHLIKLVRNHYVDSG-------- :  98
gi|213517460|GLYIN  : I----SHPVNPAKNLYFSPDVAHILKNLWSSLVNQD-------- : 115
gi|83356293|ANOGA   : F----EHPI-TKKNIYVSPDAPHLLKLLRNWFIDHG-------- :  98
gi198413217|CIOIN   : LNVESAHPHFPNEKLRFVADCPHLLKNLRTAFLNHMIYLPENIV : 123
Pelement_A1.1_DAPPU : I----SHPMDPTRKLFFSYDYTHLIKNMRNLFIDRV-------- : 111

                          140        *        160         *
gi|7155|DROBI       : --LTL----------NGKKLTKTTVQQTLNHCTKSDVS-I--L- : 126
gi|12830679|DROHE   : --LTL----------YGKKLTKTTVQQTLNYCAKSDVS-I--L- : 126
gi|221131281|HYDMA  : --IST----------NGKTVSWKYLQSFYNIDKQNPVR-L--A- : 136
gi|51102853|DROWI   : --LTI----------NGKKLTKKTIQ-ALHLCNKSDLS-I--L- : 125
gi|213517460|GLYIN  : --FYIPENIKIKHGFMTNVISSRYVKKLVELQDKSENRGIT-LS : 156
gi|83356293|ANOGA   : --FVF----------NGTIVTAQPLRDLVEGRLGAE---ITPL- : 126
gi198413217|CIOIN   : MKYSL----------PTNEVSFDWVLKLVQYEKDHE------L- : 150
Pelement_A1.1_DAPPU : --FDV----------CGEKVSFEPIKKVREIQKEF-------L- : 135

                       180        *        200        *         220
gi|7155|DROBI       : FKISENHLNVR-----SLDKQKVKLATQLFSNTTASSIRRC--- : 162
gi|12830679|DROHE   : FKISENHLNVR-----SLDKQKVNLATQLFSNTTASSIRRC--- : 162
gi|221131281|HYDMA  : PKLTDSHLESG-----SLLSMRVKLATQVFSHQVSAAMSLC--- : 172
gi|51102853|DROWI   : FKINENHINVR-----SLAKQKVKLATQLFSNTTASSIRRC--- : 161
gi|213517460|GLYIN  : FKLTEDHVN-----PTGFTKMRVHYAAEFFSRRTAKALRLC--- : 192
gi|83356293|ANOGA   : FKLNTGHLE-----LSSQERQNVRRAAELLSRTTAVSLR----- : 160
gi198413217|CIOIN   : -KLAP-KLSMKHVFPSKYEKMNVGTAAQVLSHTTSSAIR----- : 187
Pelement_A1.1_DAPPU : FFRPMRHLTFKHTQPNSLDRMKVRFAKEIFSKEMIATLRLCQNN : 179
```

Supplemental Figure S2f. Alignment of Mutator family elements from multiple species, including five new families, Mutator3_Dappu, Mutator4_Dappu, Mutator5_Dappu, Mutator8_Dappu and Mutator10_Dappu from *Daphnia pulex*.

Species abbreviations

Drosophila melanogaster = DROME
Drosophila biofasciata = DROBI
Drosophila helvetica = DROHE
Drosophila willistoni = DROWI
Drosophila hydei = DROHY
Drosophila yakuba = DROYA
Drosophila persimilis = DROPE
Drosophila pseudoobscura = DROPS
Drosophila buzzati = DROBU
Drosophila ananassae = DROAN
Mus musculus = MUSMU
Danio rerio = DANRE
Arabidopsis thaliana = ARATH
Trichomonas vaginalis = TRIVA
Fusarium oxysporum = FUSOX
Podospora anserina = PODAN
Caenorhabditis briggsae = CAEBR
Caenorhabditis elegans = CAEEL
Caenorhabditis remanei = CAERE
Bombyx mori = BOMMO
Rana pipiens = RANPI
Salmo salar salar = SALSA
Oncorhynchus mykiss = ONCMY
Pleuronectes platessa = PLEPL
Anopheles albimanus = ANOAL
Anopheles gambiae = ANOGA
Haemonchus contortus = HAECO
Saccoglossus kowalevskii = SACKO
Ixodes scapularis = IXOSC
Halorubrum lacusprofundi = HALLA
Hahella chejuensis = HAHCH
Marinobacter aquaeolei = MARAQ
Acidithiobacillus caldus = ACICA
Aspergillus niger = ASPNI
Talaromyces stipitatus = TALST
Culex quinquefasciatus = CULQU
Tribolium castaneum = TRICA
Oryza sativa = ORYSA
Aaedes aegypti = AAEAE
Ciona intestinalis = CIOIN
Trichuris muris = TRIMU
Schistosoma japonicum = SCHJA
Schistosoma mansoni = SCHMA

Antirrhinum majus = ANTMA
Bactrocera dorsalis = BACDO
Phakopsora pachyrhizi = PHAPA
Oryzias latipes = ORYLA
Zea mays = ZEAMA
Tolypocladium inflatum = TOLIN
Strongylocentrotus purpuratus = STRPU
Nematostella vectensis = NEMVE
Hydra magnipapillata = HYDMA
Glyptapanteles indiensis = GLYIN
Acyrthosiphon pisum = ACYPI
Branchiostoma floridae = BRAFL
Candida albicans = CANAL
Chelonus inanitus bracorvirus = CHEIN
Cucumis melo = CUCME
Culex pipiens = CULPI
Ciona savigny = CIOSA
Yarrowia lipolytica = YARLI
Vitis vinifera = VITVI
Equus caballus = EQUCA
Entamoeba dispar = ENTDI
Entamoeba histolytica = ENTHI
Entamoeba invadens = ENTIN
Entamoeba moshkovskii = ENTMO
Glypta fumiferanae ichnovirus = GLYFU
Helobdella robusta = HELRO
Staphylococcus epidermidis = STAEP
Meloidogyne incognita = MELIN
Schmidtea mediterranea = SCHME
Mycobacterium smegmatis = MYCSM
Nassonia vitripennis = NASVI
Phytopthora infestans = PHYIN
Phytopthora sojae = PHYSO
Trichinella spiralis = TRISP
Penicillium chrysogenum = PENCH
Medicago truncatula = MEDTR
Deinococcus radiodurans = DEIRA
Monodelphis domestica = MONDO
Bos taurus = BOSTA
Haemonchus contortus = HAECO
Daphnia pulicara = DPULI
Myotis lucifigus = MYOLU

```
                                *        20          *        40
OsNP_918808MuDR : RPLICIDGC-------------HIKTK-----FGGKLLTAVG-----M :  25
ZmM76978.1MuDR  : RPYLSVDST-------------ALNGR-----WNGHLASATG-----V :  25
AtNP_178710MuDR : RKVIVIDGT-------------HLRGR-----YGGYLVAASA-----Q :  25
Cm_MuDR         : RPVIVMDGT-------------FLKNK-----YRGQLIVAVC-----L :  25
Vv_MuDR         : RPVVAVDGT-------------FLKAK-----YLRTLFIAAC-----K :  25
Nve_MuDR        : GEMVFVDAT-------------SNTEEHN--L---KVFVMCT------ :  24
Tvmutator1      : ---HFIDAT-------------HSLLE-----FKMLFYMISA-----K :  22
Phantom_Ca1.1   : VHLFSLDAT-------------HGLVKSLNGQSNAYLFVLTG-----I :  30
Phantom_Is      : -------ST-------------RDVLPAL------GCSPLQR------ :  16
Phantom_Da1.1   : NNCLFIINSETMVCTVCVTFNQISSCP--------FLFYLQT------ :  34
Phantom_Sp      : ----FMDGN-------------FAMSPKL--FK--QLYIIRV-----P :  22
Phantom_Tv      : ATDLFCDGT-------------FKITPKH--FQKGQMLTIMI----CE :  29
Phantom_Ei      : IAYVCMDGT-------------YNSCP----ISYKQLYTIHC-----V :  26
Phantom_Cib1.1  : SQELYVDGT-------------FKCVPKD--IGAKQLLIFHM-----R :  28
Phantom_Cb1.1   : GFLYLLQMSVLSCLLIRIRF--FATAPLI--VF--RHHSLNSSHYTHT :  42
Phantom_Pi      : GAHLFVDGT-------------FRCTP----KGYHQFVTLMM-----Y :  26
Phantom_Ps      : SVSLFLDGT-------------FRSVP----RGFRQCLILMV-----D :  26
Phantom_Ec1.1   : S-TLFSDVT-------------FKTAP----TQYTQLFPVHG-----V :  25
Phantom_Ap      : CDTIYIDGT-------------FKICP----IQFTQLFTIHG-----M :  26
Phantom_Ts1.1   : CRTWGMDGT-------------FKIVP----KWYQQLFTIHA-----F :  26
Phantom_Cp1.1   : AKYWVIDGT-------------FRTAP----TLFRQLMTIIA----SC :  27
Phantom_Aa      : -----VDGT-------------FSTVP----NGFAQIFTIHG---XVG :  23
Phantom_Bf1.1   : ARRWYMDGT-------------FKVVH----APFKQLFSVHA---FVR :  28
Phantom_Tc1.3   : KDIIFGDGT-------------FDKVP----NMFYQLYTWHA-----Q :  26
Phantom_Mi      : MEHIFMDGTFSGNFKTCPRFLFFSVCP----SLFHQLFIILS------ :  38
Phantom_Cs1.1   : SSVWFADGT-------------FAVVP----SMFFQLYTIHV-----K :  26
Phantom_Hr1.1   : SELFLGDGT-------------FKCVP----SLFFQLYTVHA-----K :  26
Phantom_Gfi     : CTEWFADGT-------------FFKGK----SLFTQLYTIHG-----G :  26
Phantom_Nv1.1   : ------DGT-------------FDLCP----NIFQQIYTLHG-----L :  20
Phantom_Sm      : CEHWFADGT-------------FRVSP----NGYDQLYTIHG------ :  25
Mutator3_DAPPU  : ------------------------------------------------ :   -
Mutator4_DAPPU  : ------------------------------------------------ :   -
Mutator5_DAPPU  : SRRWYMDGT-------------FKIVK----SPFMQLWTIHA---FIR :  28
Mutator8_DAPPU  : TVRWYVDGT-------------FKIID----KPFTQLFSIHG---FLK :  28
Mutator10_DAPPU : ----MEDGT-------------FDIIA----LPFKQLWSIHG--TILG :  25
```

```
                         *         60         *         80          *
OsNP_918808MuDR : DPNDCI-FPIAMAVVEVESFVSWEWLLETL---------KSELGIDNT :  63
ZmM76978.1MuDR  : DGHNWM-YPVCFGFFQAETVDNWIWFMKQL--------KKVVGDMT-- :  62
AtNP_178710MuDR : DANFQV-FPIAFRIVNSENDEAWTWFMTKL--------TEAIPDDP-- :  62
Cm_MuDR         : DGNNQI-YPLAFGVVDRETDDSIQWFLEKL--------KGAIGEVP-- :  62
Vv_MuDR         : DGNNQI-YPLAFGIGDSENDASWEWFLQKL--------HDALGHID-- :  62
Nve_MuDR        : HNVSGA-LPLGILIASDERESTLKQGFKML-CSC-LPEYAFNGRGKEN :  69
Tvmutator1      : FPNTHA-FPIFQFVVYPNTSENIAFCLKAF---------FNWAKVK-- :  58
Phantom_Ca1.1   : IPSSRNTFPLSFMLTNYTGKITIQHWLNNL---------KTEFGIN-- :  67
Phantom_Is      : ----QG-ASNCLLPLGKQARSDVKNNMRVL-LEA-T--DDATSSPL-- :  53
Phantom_Da1.1   : -------FPFIYILMMRKTREVFEHLFAFW--------NANFFKFQ-- :  65
Phantom_Sp      : LGE-VE-ITVLYAFLQNKSRETYQELFQAV-LDK-I--AELGLQPM-- :  62
Phantom_Tv      : PSTNQY-LPLLFIFMKNRTFESYKKAFEYI-FTV----KGI-QFSK-- :  68
Phantom_Ei      : LSNGQC-FTFCHCLMTNKTELDYLNLFHLL-NEYFITHMGYLLFDE-- :  70
Phantom_Cib1.1  : KMD-VG-IASVLVLCTNQGTDLYKGIWNFL-LE-----RVPRLKEN-- :  66
Phantom_Cb1.1   : YLRLSF-AQLFFFLLTDKQTSSYEAVLKIV-LES----HPSLTQWK-- :  82
Phantom_Pi      : DQYTELYVPVFFTLATNKTEKLYNKMFKCI-------EFSLGKKPN-- :  65
Phantom_Ps      : DPARKLFLPVLHAATTSMTRKSYVRLLQCV-------QDAVGSKLV-- :  65
Phantom_Ec1.1   : VLS-YT-MSLIYALTMKR-ENTYRYTYEKV-IAF-T--RGRNLDSN-- :  64
Phantom_Ap      : KNN-NY-IPLVFCLLLDKASTTYEASFELI-ISE-C--QKLNLCLN-- :  66
Phantom_Ts1.1   : VAG-KL-VPVVYCLCTGKDIGTYGYIFQAL-IDK-A--AVLEVDLN-- :  66
Phantom_Cp1.1   : GPDHQSAYPAIFTLMTSKTELLYRSVFAKIIEAA----EAIDLDLS-- :  69
Phantom_Aa      : DGESRRFVPFVYALLPKKNETTYTAAFTALIKAA----ENIEVELE-- :  65
Phantom_Bf1.1   : CGDNIKQVPLAFALMTSRCRTDYMAVLTEL-------KKALPREIE-- :  67
Phantom_Tc1.3   : IGSSYR--PCIYILLLRKDTKISDKMFKIM--------KQLVPNMV-- :  62
Phantom_Mi      : KRGGFV-FPVLFCLLPDKTEQTYTRLFTLI--------KEIWPMFN-- :  75
Phantom_Cs1.1   : IGNNYP--PCIYFLLPNKTRQTYERMLDAI--------QNLVQNAR-- :  62
Phantom_Hr1.1   : VGS-SY-PPCLYFFLPDKTEDTYERMIQYL--------KQLAPLAN-- :  62
Phantom_Gfi     : YEGNVV--PLIYIFLPNKSKEIYRTVLMKL--------KGFIPDIK-- :  62
Phantom_Nv1.1   : YDS-SV-VPLLYAFLPSKTTDIYIYIYIKL-LKV-I--KTLKEGLN-- :  60
Phantom_Sm      : FINGEV-FPVVYALLSSRTEEAYQHLLQEI--------LILKAGLN-- :  62
Mutator3_DAPPU  : -------------MANKREKADYEKILKTI-------LEMLDNEVA-- :  26
Mutator4_DAPPU  : -------------MANKREKADYEKILKTI-------LEMLDNEVA-- :  26
Mutator5_DAPPU  : VKEQKKMVPLMYVLMSRRTRKDYKGILKII------VNKILKNNIA-- :  68
Mutator8_DAPPU  : KNDNLKQVPLCYVFMSGRTERDYRKVFEKI--------CDLVGPCD-- :  66
Mutator10_DAPPU : EGGESKSVPLLHVAMTRRKEADYVAVLELI--------KSKLHASA-- :  63
```

```
                          100         *        120         *        140
OsNP_918808MuDR : YPWTIMTNKQKGLIPAVKKVF-PDTEHRFCVRHLYSNFQEKFK----- : 105
ZmM76978.1MuDR  : -LLAICSDAQKGLMHAVNEVF-PYAERRECFRHLMGNYVKHHA----- : 103
AtNP_178710MuDR : -ELVFVSDRHNSVYASIRKVY-PMSSHAACVVHLKRNIEASFK----- : 103
Cm_MuDR         : -NLGFVTDRKTCFAKGISSVF-PSAFHGLCVQHLSQNLHDKYK----- : 103
Vv_MuDR         : -DLFVISDRHGSIEKAVHKVF-PHARHGVCTYHVGQNLKTKFK----- : 103
Nve_MuDR        : GPKVILTDNCKEERNALKSIW-PLSTLLLCTFHMLQQLWRWLH----- : 111
Tvmutator1      : -PKYFMSDCAQEIENAIINSF-PEVILHWCAVHVMRAFRKNLK----- :  99
Phantom_Ca1.1   : -PTQFVIDADPAEISGIQSIF-KDTKIVLCYFHVLRAVTIKLK----- : 108
Phantom_Is      : -QVHVGIDFEKDEVNAFRECA-PGAKVHGCLFHLAQSIWRRIQ----- :  94
Phantom_Da1.1   : -PKSFTTDYEIAFRKALKTVF-PTAMINGCWFHYCQALRRNSL----- : 106
Phantom_Sp      : -LATIITDFEIAAMRAAEAVF-TPHRNQGCFYHLTQSTWRRVQVNITS : 108
Phantom_Tv      : -LIQIHCDFEKGMIEALQQIF-PKVRIIGCLFHFKQALHRKLV----- : 109
Phantom_Ei      : -NTSVMLDFETAVINALIG---FKCCVLGCYFHFTQCIWRKVQ----- : 109
Phantom_Cib1.1  : -LKFVMMDFERALIKSTKETM-PSVVIRGCWFHFTKAISTYWQ----- : 107
Phantom_Cb1.1   : -PATVICDYETGLKNAFESQF-PRASVQGCLFHLVQSWRKKAE----- : 123
Phantom_Pi      : -PANVVCDFEAALIFAIRDNF-PSTRIVGCLFHFKQACSRKLK----- : 106
Phantom_Ps      : -PKDVVCDFESSLIGALREFF-PDVTIIGCLFHFKQACRRKMK----- : 106
Phantom_Ec1.1   : -PTLCMMDFEIASINVIQLLL-PNVQVN-CLFHFSQSL-RKIS----- : 103
Phantom_Ap      : -PKTVFADFEMAIHVAVNKVW-PSTRLRGCRFHLGQAWFRQIQ----- : 107
Phantom_Ts1.1   : -PDTIICDFETALIPAIRGYF-PNTRVQGCYFHFCQAVHRKVG----- : 107
Phantom_Cp1.1   : -PSVILSDFEKAIINASKLEF-PESKHNCCFFHFSKNLLDHIR----- : 110
Phantom_Aa      : -PETILADFELAEINAVKACW-PDSEVHGCYFHFSQSMFXKLK----- : 106
Phantom_Bf1.1   : -LEEGVTDFEVGLCQAFQEVF-QGTRMKGCAFHWCQAIWRKVQ----- : 108
Phantom_Tc1.3   : -SQKILLDFEKACMNAARTAF-PESEKKGCYFHLCQSLIRKIN----- : 103
Phantom_Mi      : -PTSISCDFERAIHNSIRTCF-PESSIFCCFFHLRQNLRKHIS----- : 116
Phantom_Cs1.1   : -PQQVFLDFEAAAILAFKHRF-GDTVVSGCHFHFCQSVLRKVT----- : 103
Phantom_Hr1.1   : -PNQILLDFELAAINAFRHEF-PNAHISGCFFHFCQSILRKVQ----- : 103
Phantom_Gfi     : -LQRLMVDFEMAFIIACREVF-PNVKIKGCYFHFRQAISRHIC----- : 103
Phantom_Nv1.1   : -PEKIMIDMELSFKNAFLNEF-PNASVSYCYFHFNQALYRNLA----- : 101
Phantom_Sm      : -PASIMVDFELAXIRAFQSTF-PTATITGCMFHFGQCVWRKLQ----- : 103
Mutator3_DAPPU  : -VEEVVTDFEKATWRAFQSVF-PDARLFGCAFHWTQALFRNLK----- :  67
Mutator4_DAPPU  : -VEEVVTDFEKATWRAFQSVF-PDARLFGCAFHWTQALFRNLK----- :  67
Mutator5_DAPPU  : -VTQCVMDFEKAVWLALGDVFGDDVELFGCGFHWTQCIFRRLK----- : 110
Mutator8_DAPPU  : -VKEFVSDFERAIWLGAKNAFPDGVKMFGCAFHWCQAVY--------- : 104
Mutator10_DAPPU : -IKPFTSDHEKALWKAVARVF-PEVQHWGFSFHQIQAQMRRLK----- : 104
```

```
                                *        160         *        180         *
OsNP_918808MuDR : ---------GEIRHLNARFEVVAAAPAAIAPHHVLLLHLGCAPYRP-- : 142
ZmM76978.1MuDR  : ---------GSEHMYPAA----------RAYRRDVF-----EHHVS-- : 125
AtNP_178710MuDR : -------YEQLGTLVS------------SAARAYRL------------ : 120
Cm_MuDR         : -------NDTVATLFYNASRTYRESTFVEAWRHLLSFPNGSGKYLNDV : 144
Vv_MuDR         : -----------------NPVIHKLFH--DAAHAYRV------------ : 120
Nve_MuDR        : -------ESKNNVNLADR--PF--IL--NLFKKS-------LYAET-- : 137
Tvmutator1      : -------------DYAFESSDKLILD--TKMNYLAY-----GRNGK-- : 125
Phantom_Ca1.1   : -------EVVILPDKEQQKAIHDLIT--QDLKR--------ILFNS-- : 137
Phantom_Is      : -------KLGMHARFIGD--SS--YP--LIRKEFIA-----LPLRC-- : 122
Phantom_Da1.1   : -------KIRNFGAMLKN--TD--CY--KWYRTFLS-----LPLIQ-- : 134
Phantom_Sp      : ITVFFMQELGLGEHYRED--AD--FR--HFAGMLDG-----LAFLP-- : 143
Phantom_Tv      : ------------ALYTKN--FNTLQN--SLFKLYSI-----TPFMS-- : 134
Phantom_Ei      : -------PLGLSQQYYPETS----IW--SIVNNKT------LDFLK-- : 136
Phantom_Cib1.1  : -------DLGLKNVPSSS------VQ--DILSLSWV-----LPLLP-- : 133
Phantom_Cb1.1   : ------------------------------------------------ :   -
Phantom_Pi      : -------EYSMPKDESA-------IA--MSFGVFDM-----LTVVD-- : 131
Phantom_Ps      : -------SIGLPVGEIK-------VA--MSRNVFDI-----LTVID-- : 131
Phantom_Ec1.1   : -------SLVLTQKYMTTERNTH-----KCASMLFG-----LPFVP-- : 132
Phantom_Ap      : -------SLGLVNEYKNKNSEIG-----KYLKTFFG-----LSFLS-- : 136
Phantom_Ts1.1   : -------ELGLKTRYRQHEETK------RKIRMLLA-----TAFLP-- : 135
Phantom_Cp1.1   : -------SEKMSHLFDQNQHNF------NIYKKVQA-----LAFLP-- : 138
Phantom_Aa      : -------QLHLQKSYGNDNTVH------LTFKKILA-----LAFLP-- : 134
Phantom_Bf1.1   : -------ELGLQRAYNNDHRVY------SWVRRLMA-----LPFLP-- : 136
Phantom_Tc1.3   : -------SVGLKTEYESDIDVK------LRLKSLVA-----LAFVP-- : 131
Phantom_Mi      : -------QSNLLNLYNNDPD----FA--LKCKMIIS-----LAFVP-- : 144
Phantom_Cs1.1   : -------SLGLKSRYETDSNFR------LLVKCLPA-----LSFVP-- : 131
Phantom_Hr1.1   : -------FLGLKGEYERDAHLN------MLIKSLCS-----LAFVP-- : 131
Phantom_Gfi     : -------QSSLGSLYNRDLELA------HEIKKICA-----LMFVP-- : 131
Phantom_Nv1.1   : -------EHNLKTLYDSDES----FS--QKIKTLSA-----LAFVP-- : 129
Phantom_Sm      : -------AEGFSERYRNEPD----FA--LLVKRLLA-----LAFVP-- : 131
Mutator3_DAPPU  : -------KNGLVHLYRYDANVQ------KVCKRAMC-----LHLLP-- :  95
Mutator4_DAPPU  : -------KNGLVHLYRYDANVQ------KVCKRAMC-----LHLLP-- :  95
Mutator5_DAPPU  : -------KLGLTSAYRSKQS--------NQVRTICR-----YSFL--- : 135
Mutator8_DAPPU  : ------------------------------------------------ :   -
Mutator10_DAPPU : ------DKEHLSIAYQTD------IY--SKHRSTHS-----LIR---- : 129
```

```
                       200         *       220         *       240
OsNP_918808MuDR : --------------------------------------PQTGAAIANF : 152
ZmM76978.1MuDR  : ---K----------------------------------VRNVHKIAEY : 136
AtNP_178710MuDR : --------------------------------------TDFNRIFAEV : 130
Cm_MuDR         : GIARWSRVHCPGRRYNMMTTNIAESMNSILKEPRDLPIASFLENVRAL : 192
Vv_MuDR         : --------------------------------------SEFNFIFGQL : 130
Nve_MuDR        : ---E----------------------------------QEFENSFSEL : 148
Tvmutator1      : ---K----------------------------------EWIEPTFKKI : 136
Phantom_Ca1.1   : --------------------------------------ENTENDIQKF : 147
Phantom_Is      : ---F----------------------------------RRLRQIS--- : 130
Phantom_Da1.1   : ---R----------------------------------PKIQESL-QF : 144
Phantom_Sp      : ---E----------------------------------GDVVRGF-EY : 153
Phantom_Tv      : ---H----------------------------------EEFVLTM-HI : 144
Phantom_Ei      : ---Q----------------------------------VKYHTDLILL : 147
Phantom_Cib1.1  : --------------------------------------KKHFPTAIAF : 143
Phantom_Cb1.1   : ---K-------------------------------------------- : 124
Phantom_Pi      : ---P----------------------------------DKISVQGVAW : 142
Phantom_Ps      : ---P----------------------------------TKIAVQGIAW : 142
Phantom_Ec1.1   : ----------------------------------------LVDVNATF : 140
Phantom_Ap      : ---P----------------------------------PDVNDCFTDD : 147
Phantom_Ts1.1   : --------------------------------------EPQVDMGVSL : 145
Phantom_Cp1.1   : ---S----------------------------------EEIPEAFATL : 149
Phantom_Aa      : ---P----------------------------------QDIPTVFNKL : 145
Phantom_Bf1.1   : ---H----------------------------------KAIQPALRAL : 147
Phantom_Tc1.3   : ---M----------------------------------QDVRKNF-DF : 141
Phantom_Mi      : ---E----------------------------------NDVINAL-NV : 154
Phantom_Cs1.1   : ---V----------------------------------SDLVFRFEEL : 142
Phantom_Hr1.1   : ---L----------------------------------NELTDVFELL : 142
Phantom_Gfi     : ---S----------------------------------ENVLSSYEKL : 142
Phantom_Nv1.1   : ---P----------------------------------DDIKDCFEDI : 140
Phantom_Sm      : ---P----------------------------------QDVIDLFEHL : 142
Mutator3_DAPPU  : ---P----------------------------------TKIKKVFAVL : 106
Mutator4_DAPPU  : ---P----------------------------------TKIKKVFAVL : 106
Mutator5_DAPPU  : ------------------------------------------------ :   -
Mutator8_DAPPU  : ------------------------------------------------ :   -
Mutator10_DAPPU : ------------------------------------------------ :   -
```

```
                            *       260         *       280
OsNP_918808MuDR : VPCIAC----------CRRCSLHPLTAL--------------HLIYAT : 176
ZmM76978.1MuDR  : LDQHHKFLWYRSGFNKDIKCDYITNNMA--------------EVY-NN : 169
AtNP_178710MuDR : RAKHG-----------PCADYLEGIGFE--------------HWTRSL : 153
Cm_MuDR         : LQPLVLGASRRSIKVTSTLTKWAELVIQKKQEGALTMKVNPIDCYQFH : 240
Vv_MuDR         : ----------------EMIDPRAARYLM--------------DIGV-- : 146
Nve_MuDR        : LNDDHCMENPTLVSYLQKLYNDKESFALCFRKELPVRGNHTNNFAEAQ : 196
Tvmutator1      : L---------------EIAEKFPE--FS--------------KYIQNQ : 153
Phantom_Ca1.1   : L---------------EKYASFRR--FK--------------SYFQKQ : 164
Phantom_Is      : ----------------HTRKTAAG--LL-------------------- : 140
Phantom_Da1.1   : LRTNCWSCQK------TG-TTFNV--FI--------------TLKRNG : 169
Phantom_Sp      : VNA-------------VAPPEGNA--LL--------------QYFERT : 172
Phantom_Tv      : INQ-------------NKVDSIKD--YI--------------DYFNKV : 163
Phantom_Ei      : TTKPSVVNSM------IKQNNLME--LL--------------MYFKLN : 173
Phantom_Cib1.1  : YEKKAEEVEP------EYQDKVNK--FI--------------AYLKKQ : 169
Phantom_Cb1.1   : ------------------------------------------------ :   -
Phantom_Pi      : VKREIKKRCDRKSITYSR-EKWNK--FW--------------AYFAKT : 173
Phantom_Ps      : VKAKICSVCEVKGLEYSR-SKWRT--FW--------------SYFQRT : 173
Phantom_Ec1.1   : VRKQGIVE--------SAEENLDD--LM--------------GYVERV : 164
Phantom_Ap      : LIS-------------ILPQDYRVERFT--------------DYILEN : 168
Phantom_Ts1.1   : LEA-------------GTTGTLAA--LF--------------QYFRQE : 164
Phantom_Cp1.1   : KK--------------EAPENLHE--FF--------------AYVEEF : 167
Phantom_Aa      : KK--------------STPRNMVQ--FM--------------KYVGEF : 163
Phantom_Bf1.1   : QS--------------RAAGALTD--LA--------------DYVQKT : 165
Phantom_Tc1.3   : LAALF-----------PNEDKFNE--IL--------------TYHL-- : 160
Phantom_Mi      : LEN-------------ELDDRFEP--LI--------------SWFVST : 173
Phantom_Cs1.1   : AENFAA----------NYPNDMACDELM--------------TYFDGT : 166
Phantom_Hr1.1   : CQQF------------PDGDELTP--LL--------------AYVDQT : 162
Phantom_Gfi     : KKSSYYA---------ENIEVLSE--LL--------------SYFETT : 165
Phantom_Nv1.1   : VQEVFSE---------SNSEGVDG--YL--------------QYFQKN : 163
Phantom_Sm      : IED-------------PAYRDIEV--IC--------------DYMEDN : 161
Mutator3_DAPPU  : KTNAGA----------LHIDQLDN--FF--------------TYIENT : 128
Mutator4_DAPPU  : KTNAGA----------LHIDQLDN--FF--------------TYIE-- : 126
Mutator5_DAPPU  : ------------------------------------------------ :   -
Mutator8_DAPPU  : ------------------------------------------------ :   -
Mutator10_DAPPU : ------------------------------------------------ :   -
```

```
                        *       300         *       320         *
OsNP_918808MuDR : TATA-------------------------------------------- : 180
ZmM76978.1MuDR  : WVKD-------------------HKDLPVC------------------ : 180
AtNP_178710MuDR : FVGNRYNVMTSN--IAESLNNVLTLEITR------------------- : 180
Cm_MuDR         : VKDLDKEEVVNLQTKECTCKEFQAEQLPCSHAIAAARVRNINVYSLCA : 288
Vv_MuDR         : ------------------------------------------------ :   -
Nve_MuDR        : FLVL-KDIILRR--TKEYNVVGLLDKLTI------------------- : 222
Tvmutator1      : WISN------QE--RWTAAERDENLALTN------------------- : 174
Phantom_Ca1.1   : WMSKI------------------------------------------- : 169
Phantom_Is      : WMHG-IALRLPR--LLYLGRSTKDPRFPI------------------- : 166
Phantom_Da1.1   : WVKD-YFSFLI-------------LLYPC------------------- : 184
Phantom_Sp      : YIRG-PEQQRQG--QQFNMRYRLPAIFPP------------------- : 198
Phantom_Tv      : WL--------------------PHY--------------------- : 168
Phantom_Ei      : WLEK----------------------WPI------------------- : 180
Phantom_Cib1.1  : WLRI----------------------AEIV------------------ : 177
Phantom_Cb1.1   : ------------------------------------------------ :   -
Phantom_Pi      : WL--------------------ETYPP------------------- : 180
Phantom_Ps      : WL--------------------ETYPP------------------- : 180
Phantom_Ec1.1   : YVGR---------HGRGRRRPEAPRFPQ------------------ : 183
Phantom_Ap      : YISE-------------------TSKYPP------------------- : 178
Phantom_Ts1.1   : WMTD----------------------ERL------------------ : 171
Phantom_Cp1.1   : YVLG-----KVTRVNKKKKVFRSDPLFPP------------------- : 191
Phantom_Aa      : YIHG-------------PVGKKRGPTFPP------------------- : 179
Phantom_Bf1.1   : WVDS--------------------TVFPP------------------- : 174
Phantom_Tc1.3   : --------------------------LC------------------ : 162
Phantom_Mi      : YIGRIRCNGTRA-----------NPIFSI------------------- : 191
Phantom_Cs1.1   : YIRG-----------RRVGARDAPPIYPP------------------- : 184
Phantom_Hr1.1   : YIRG-----------IQMRNRRTPPRFPP------------------- : 180
Phantom_Gfi     : WIGT----------PKRRNRGQKAPLFEI------------------- : 184
Phantom_Nv1.1   : YIGM------------KIGRRDIELRFTI------------------- : 180
Phantom_Sm      : FIGR-LRRRRRG--P---------PRFSI------------------- : 178
Mutator3_DAPPU  : ------------------------------------------------ :   -
Mutator4_DAPPU  : ------------------------------------------------ :   -
Mutator5_DAPPU  : ------------------------------------------------ :   -
Mutator8_DAPPU  : ------------------------------------------------ :   -
Mutator10_DAPPU : ------------------------------------------------ :   -
```

```
                   340         *       360         *       380
OsNP_918808MuDR : ----------------------DVI-ASGMGDVTRCPRTAAVLT--VL : 203
ZmM76978.1MuDR  : ----------------------DLAEKIREMTMELFHRRRRIGH--KL : 204
AtNP_178710MuDR : ----------------------RQA-ARTEDNILPPKVHDMVIE--NY : 203
Cm_MuDR         : NYYTNECLLAAYAEAVYPVGNQSDW-KTSEDYVHMTVLPPKVVK--RV : 333
Vv_MuDR         : ----------------------DRWTRSYSTEKRYNIMTTGIV----- : 167
Nve_MuDR        : ----------------------DLE-DHYKNKLLSIADGSFDGT--YR : 245
Tvmutator1      : ----------------------NIS-ESINKKIKYYYFGGTIFM--RF : 197
Phantom_Ca1.1   : ----------------------NMWLRTENNTFDILLLTNNLTE--NF : 193
Phantom_Is      : ----------------------SMW-NCKRVTEVSLPRTNSSVK--GW : 189
Phantom_Da1.1   : ----------------------RGY-YNFGQKCATQRRDLRPYK--VY : 207
Phantom_Sp      : ----------------------HVW-NVHQATIDGNPRTNNACE--SW : 221
Phantom_Tv      : ----------------------NLI-SQYNNATAIF--TNDCLE--SM : 189
Phantom_Ei      : ----------------------KLW-CQFNQKL----RTDNLSE--SY : 199
Phantom_Cib1.1  : ----------------------SLW--------GSPIRTNNICE--SF : 193
Phantom_Cb1.1   : ------------------------------------------------ :   -
Phantom_Pi      : ----------------------DLW-NIYGVQRQIVNRTNNPLE--RF : 203
Phantom_Ps      : ----------------------EYW-NVFGMRRDIISRTNNPLE--RF : 203
Phantom_Ec1.1   : ----------------------ETW-KVYTSVLNGGHRINNTVE--GW : 206
Phantom_Ap      : ----------------------EMW-ASFTSSLA---RTTNSCE--SF : 198
Phantom_Ts1.1   : ----------------------PLW-NVHSVNI----RTNNLLE--GW : 190
Phantom_Cp1.1   : ----------------------ALW-SVYDSVKQRVPRTTNQIE--AF : 214
Phantom_Aa      : ----------------------KLW-SVHNNIMNGVXRTSNNLE--GW : 202
Phantom_Bf1.1   : ----------------------RRW-SVYMQAIRT------------- : 186
Phantom_Tc1.3   : ----------------------CFI-YLYVKYRKKPSNFNSNFN--YV : 185
Phantom_Mi      : ----------------------PLW-NVHTRTILNIHR---------- : 206
Phantom_Cs1.1   : ----------------------SLW-NHFTSASTALPKTTNCCE--GY : 207
Phantom_Hr1.1   : ----------------------ELW-NRTNDALACAPKTTNASE--GY : 203
Phantom_Gfi     : ----------------------EMW-NHYSSVVNGEPRTNNNVE--GW : 207
Phantom_Nv1.1   : ----------------------NMW-NQYENTVNNLPRTNNSVE--GW : 203
Phantom_Sm      : ----------------------QLW-SQFSRVIDNLPRSNNAIE--GW : 201
Mutator3_DAPPU  : ------------------------------------------------ :   -
Mutator4_DAPPU  : --------------------------NT-------------------- : 128
Mutator5_DAPPU  : ------------------------------------------------ :   -
Mutator8_DAPPU  : ------------------------------------------------ :   -
Mutator10_DAPPU : ------------------------------------------------ :   -
```

```
                       *        400        *        420        *
OsNP_918808MuDR : MAAPPRFC---------------------------------------- : 211
ZmM76978.1MuDR  : HGIILPSV----------------------------LAILKARTRGLG : 224
AtNP_178710MuDR : EKGAGCVV---------------------------------------- : 211
Cm_MuDR         : GRPKKKRIPSVGEAPKLHKCGRCKQIGHNRLTCTNPISYTDKSSIQDF : 381
Vv_MuDR         : ------------------------------------------------ :   -
Nve_MuDR        : HRFMGKGKGKGKGSFGFNVPDRKELDGYLSSVESFGNNTFKVGSSSDG : 293
Tvmutator1      : DRFVMKLIDFIVPSFYYRISQDIRLRDK-IPNPLPSEKKPKKSTIRLD : 244
Phantom_Ca1.1   : FSVLKTVI---------------------------LKNQPN------- : 207
Phantom_Is      : RRVFQNYA---------------------------GASHPT------- : 203
Phantom_Da1.1   : IFLIRITS-----------------------VDMSMSVCPS------- : 225
Phantom_Sp      : N----------------------------------------------- : 222
Phantom_Tv      : HSEFSSLK------------------------------HPN------- : 200
Phantom_Ei      : HSALVKRL---------------------------CCKKPT------- : 213
Phantom_Cib1.1  : HRWVSKRL----------------------------GNHPN------- : 206
Phantom_Cb1.1   : ------------------------------------------------ :   -
Phantom_Pi      : HRELNARI----------------------------KTHPS------- : 216
Phantom_Ps      : HRELNKRF----------------------------NAHPP------- : 216
Phantom_Ec1.1   : RSKFQKLMVEHH------------------------------------ : 218
Phantom_Ap      : HSKINAMF---------------------------YSAHPN------- : 212
Phantom_Ts1.1   : HNRLNRKA---------------------------------------- : 198
Phantom_Cp1.1   : HRRWNSLV---------------------------------------- : 222
Phantom_Aa      : HNKWNSLFKNKVSFXGVLRQFQLEERSASTQVXRXLQLEPTRKXMKAS : 250
Phantom_Bf1.1   : ------------------------------------------------ :   -
Phantom_Tc1.3   : TQTWVVSE---------------------------------------- : 193
Phantom_Mi      : ------------------------------------------------ :   -
Phantom_Cs1.1   : HNALQSLF---------------------------LCKHPS------- : 221
Phantom_Hr1.1   : HHELNAMF---------------------------SCHHPG------- : 217
Phantom_Gfi     : YRRLSSRT---------------------------------------- : 215
Phantom_Nv1.1   : HR---------------------------------------------- : 205
Phantom_Sm      : HNAFNNVV---------------------------GFAHPT------- : 215
Mutator3_DAPPU  : ------------------------------------------------ :   -
Mutator4_DAPPU  : ------------------------------------------------ :   -
Mutator5_DAPPU  : ------------------------------------------------ :   -
Mutator8_DAPPU  : ------------------------------------------------ :   -
Mutator10_DAPPU : ------------------------------------------------ :   -
```

```
                       440         *         460         *         480
OsNP_918808MuDR : ------HRRALHPLVATP------------------------------ : 223
ZmM76978.1MuDR  : HLSIVKCDNYMAEVRDSTNCMTKHVVNAE------------------- : 253
AtNP_178710MuDR : LKIGDGLYEVIEKRGSAFAVNLW------------------------- : 234
Cm_MuDR         : SSTLKGIFPNFSYYNVTLFLCLYFKRFNFCLQKLSLCSYIIIVKRLGF : 429
Vv_MuDR         : ------------------------------------------------ :   -
Nve_MuDR        : SQRSVYFLYIYSLYNTFEWHTAEYAMHHLKENTSEKWDILQYTTRIHC : 341
Tvmutator1      : TEKCIMLTDKIKSLIVNSSANLNTLQLGLDDLLDKVVKAAKVQKRMTQ : 292
Phantom_Ca1.1   : KRLDSLVYVLVEIVIPRFW----------------------------- : 226
Phantom_Is      : ------------------------------------------------ :   -
Phantom_Da1.1   : ------VYFYAK------------------------------------ : 231
Phantom_Sp      : ------------------------------------------------ :   -
Phantom_Tv      : ------IYEAIKKISQIQL----------------------------- : 213
Phantom_Ei      : ------LNKLLITLMKHEDSEL-------------------------- : 229
Phantom_Cib1.1  : ------FYVFI------------------------------------- : 211
Phantom_Cb1.1   : ------------------------------------------------ :   -
Phantom_Pi      : ------LNNFVRVIEQFA------------------------------ : 228
Phantom_Ps      : ------MKTFVTSLENLA------------------------------ : 228
Phantom_Ec1.1   : ------------------------------------------------ :   -
Phantom_Ap      : ------IYQFINILL--------------------------------- : 221
Phantom_Ts1.1   : GKSHNGLYELLQLLIAEQGVM--------------------------- : 219
Phantom_Cp1.1   : -GLNPGVFKVIEELRKEELQT--------------------------- : 242
Phantom_Aa      : EEKEHKLLKIVKEYSKYQNKMDFVLGVALILMK--------------- : 283
Phantom_Bf1.1   : ------------------------------------------------ :   -
Phantom_Tc1.3   : ------FYVVLLIYKSKNIFILQV------------------------ : 211
Phantom_Mi      : ------------------------------------------------ :   -
Phantom_Cs1.1   : ------VWKLLNGLKKDV------------------------------ : 233
Phantom_Hr1.1   : ------VWKLFQGIKNDI------------------------------ : 229
Phantom_Gfi     : LKNNSTFWAILDLIISEQ------------------------------ : 233
Phantom_Nv1.1   : ------------------------------------------------ :   -
Phantom_Sm      : ------TTKLARKLQQEQ------------------------------ : 227
Mutator3_DAPPU  : ------------------------------------------------ :   -
Mutator4_DAPPU  : ------------------------------------------------ :   -
Mutator5_DAPPU  : ------------------------------------------------ :   -
Mutator8_DAPPU  : ------------------------------------------------ :   -
Mutator10_DAPPU : ------------------------------------------------ :   -
```

```
                           *        500       *        520
OsNP_918808MuDR : ------------------------------------------------ :   -
ZmM76978.1MuDR  : ------------------------------------------------ :   -
AtNP_178710MuDR : ------------------------------------------------ :   -
Cm_MuDR         : RVRLLSSIYYIHFRMAVPSEKYFPATVSCQVHKIGSLIKDKLTKDQLQ : 477
Vv_MuDR         : ------------------------------------------------ :   -
Nve_MuDR        : ITFFMPRHRK-------------------------------------- : 351
Tvmutator1      : YFSKLSIPMEIKLSILTQITEFGCNTPQFIVENAKSFQDKIITDFHLQ : 340
Phantom_Ca1.1   : ------------------------------------------------ :   -
Phantom_Is      : ------------------------------------------------ :   -
Phantom_Da1.1   : ------------------------------------------------ :   -
Phantom_Sp      : ------------------------------------------------ :   -
Phantom_Tv      : ------------------------------------------------ :   -
Phantom_Ei      : ------------------------------------------------ :   -
Phantom_Cib1.1  : ------------------------------------------------ :   -
Phantom_Cb1.1   : ------------------------------------------------ :   -
Phantom_Pi      : ------------------------------------------------ :   -
Phantom_Ps      : ------------------------------------------------ :   -
Phantom_Ec1.1   : ------------------------------------------------ :   -
Phantom_Ap      : ------------------------------------------------ :   -
Phantom_Ts1.1   : ------------------------------------------------ :   -
Phantom_Cp1.1   : ------------------------------------------------ :   -
Phantom_Aa      : ------------------------------------------------ :   -
Phantom_Bf1.1   : ------------------------------------------------ :   -
Phantom_Tc1.3   : ------------------------------------------------ :   -
Phantom_Mi      : ------------------------------------------------ :   -
Phantom_Cs1.1   : ------------------------------------------------ :   -
Phantom_Hr1.1   : ------------------------------------------------ :   -
Phantom_Gfi     : ------------------------------------------------ :   -
Phantom_Nv1.1   : ------------------------------------------------ :   -
Phantom_Sm      : ------------------------------------------------ :   -
Mutator3_DAPPU  : ------------------------------------------------ :   -
Mutator4_DAPPU  : ------------------------------------------------ :   -
Mutator5_DAPPU  : ------------------------------------------------ :   -
Mutator8_DAPPU  : ------------------------------------------------ :   -
Mutator10_DAPPU : ------------------------------------------------ :   -
```

```
                      *       540         *       560         *
OsNP_918808MuDR : ------------------------------------------------ :   -
ZmM76978.1MuDR  : ------------------------------------------------ :   -
AtNP_178710MuDR : ------------------------------------------------ :   -
Cm_MuDR         : MFEKTIFGPLLNVNMVFNGQLIHHFLLRQIPEDGNADGICFSVLGKNV : 525
Vv_MuDR         : ------------------------------------------------ :   -
Nve_MuDR        : ------------------------------------------------ :   -
Tvmutator1      : IYTTL------------------------------------------- : 345
Phantom_Ca1.1   : ------------------------------------------------ :   -
Phantom_Is      : ------------------------------------------------ :   -
Phantom_Da1.1   : ------------------------------------------------ :   -
Phantom_Sp      : ------------------------------------------------ :   -
Phantom_Tv      : ------------------------------------------------ :   -
Phantom_Ei      : ------------------------------------------------ :   -
Phantom_Cib1.1  : ------------------------------------------------ :   -
Phantom_Cb1.1   : ------------------------------------------------ :   -
Phantom_Pi      : ------------------------------------------------ :   -
Phantom_Ps      : ------------------------------------------------ :   -
Phantom_Ec1.1   : ------------------------------------------------ :   -
Phantom_Ap      : ------------------------------------------------ :   -
Phantom_Ts1.1   : ------------------------------------------------ :   -
Phantom_Cp1.1   : ------------------------------------------------ :   -
Phantom_Aa      : ------------------------------------------------ :   -
Phantom_Bf1.1   : ------------------------------------------------ :   -
Phantom_Tc1.3   : ------------------------------------------------ :   -
Phantom_Mi      : ------------------------------------------------ :   -
Phantom_Cs1.1   : ------------------------------------------------ :   -
Phantom_Hr1.1   : ------------------------------------------------ :   -
Phantom_Gfi     : ------------------------------------------------ :   -
Phantom_Nv1.1   : ------------------------------------------------ :   -
Phantom_Sm      : ------------------------------------------------ :   -
Mutator3_DAPPU  : ------------------------------------------------ :   -
Mutator4_DAPPU  : ------------------------------------------------ :   -
Mutator5_DAPPU  : ------------------------------------------------ :   -
Mutator8_DAPPU  : ------------------------------------------------ :   -
Mutator10_DAPPU : ------------------------------------------------ :   -
```

```
OsNP_918808MuDR : --- :   -
ZmM76978.1MuDR  : --- :   -
AtNP_178710MuDR : --- :   -
Cm_MuDR         : RFT : 528
Vv_MuDR         : --- :   -
Nve_MuDR        : --- :   -
Tvmutator1      : --- :   -
Phantom_Ca1.1   : --- :   -
Phantom_Is      : --- :   -
Phantom_Da1.1   : --- :   -
Phantom_Sp      : --- :   -
Phantom_Tv      : --- :   -
Phantom_Ei      : --- :   -
Phantom_Cib1.1  : --- :   -
Phantom_Cb1.1   : --- :   -
Phantom_Pi      : --- :   -
Phantom_Ps      : --- :   -
Phantom_Ec1.1   : --- :   -
Phantom_Ap      : --- :   -
Phantom_Ts1.1   : --- :   -
Phantom_Cp1.1   : --- :   -
Phantom_Aa      : --- :   -
Phantom_Bf1.1   : --- :   -
Phantom_Tc1.3   : --- :   -
Phantom_Mi      : --- :   -
Phantom_Cs1.1   : --- :   -
Phantom_Hr1.1   : --- :   -
Phantom_Gfi     : --- :   -
Phantom_Nv1.1   : --- :   -
Phantom_Sm      : --- :   -
Mutator3_DAPPU  : --- :   -
Mutator4_DAPPU  : --- :   -
Mutator5_DAPPU  : --- :   -
Mutator8_DAPPU  : --- :   -
Mutator10_DAPPU : --- :   -
```

Supplemental Figure S2g. Alignment of PIF family elements from multiple species, including one new family, Dappu_PIF1, from *Daphnia pulex*.

Species abbreviations

Drosophila melanogaster = DROME
Drosophila biofasciata = DROBI
Drosophila helvetica = DROHE
Drosophila willistoni = DROWI
Drosophila hydei = DROHY
Drosophila yakuba = DROYA
Drosophila persimilis = DROPE
Drosophila pseudoobscura = DROPS
Drosophila buzzati = DROBU
Drosophila ananassae = DROAN
Mus musculus = MUSMU
Danio rerio = DANRE
Arabidopsis thaliana = ARATH
Trichomonas vaginalis = TRIVA
Fusarium oxysporum = FUSOX
Podospora anserina = PODAN
Caenorhabditis briggsae = CAEBR
Caenorhabditis elegans = CAEEL
Caenorhabditis remanei = CAERE
Bombyx mori = BOMMO
Rana pipiens = RANPI
Salmo salar salar = SALSA
Oncorhynchus mykiss = ONCMY
Pleuronectes platessa = PLEPL
Anopheles albimanus = ANOAL
Anopheles gambiae = ANOGA
Haemonchus contortus = HAECO
Saccoglossus kowalevskii = SACKO
Ixodes scapularis = IXOSC
Halorubrum lacusprofundi = HALLA
Hahella chejuensis = HAHCH
Marinobacter aquaeolei = MARAQ
Acidithiobacillus caldus = ACICA
Aspergillus niger = ASPNI
Talaromyces stipitatus = TALST
Culex quinquefasciatus = CULQU
Tribolium castaneum = TRICA
Oryza sativa = ORYSA
Aaedes aegypti = AAEAE
Ciona intestinalis = CIOIN
Trichuris muris = TRIMU
Schistosoma japonicum = SCHJA
Schistosoma mansoni = SCHMA
Antirrhinum majus = ANTMA
Bactrocera dorsalis = BACDO
Phakopsora pachyrhizi = PHAPA
Oryzias latipes = ORYLA
Zea mays = ZEAMA
Tolypocladium inflatum = TOLIN
Strongylocentrotus purpuratus = STRPU
Nematostella vectensis = NEMVE
Hydra magnipapillata = HYDMA
Glyptapanteles indiensis = GLYIN
Acyrthosiphon pisum = ACYPI
Branchiostoma floridae = BRAFL
Candida albicans = CANAL
Chelonus inanitus bracorvirus = CHEIN
Cucumis melo = CUCME
Culex pipiens = CULPI
Ciona savigny = CIOSA
Yarrowia lipolytica = YARLI
Vitis vinifera = VITVI
Equus caballus = EQUCA
Entamoeba dispar = ENTDI
Entamoeba histolytica = ENTHI
Entamoeba invadens = ENTIN
Entamoeba moshkovskii = ENTMO
Glypta fumiferanae ichnovirus = GLYFU
Helobdella robusta = HELRO
Staphylococcus epidermidis = STAEP
Meloidogyne incognita = MELIN
Schmidtea mediterranea = SCHME
Mycobacterium smegmatis = MYCSM
Nassonia vitripennis = NASVI
Phytopthora infestans = PHYIN
Phytopthora sojae = PHYSO
Trichinella spiralis = TRISP
Penicillium chrysogenum = PENCH
Medicago truncatula = MEDTR
Deinococcus radiodurans = DEIRA
Monodelphis domestica = MONDO
Bos taurus = BOSTA
Haemonchus contortus = HAECO
Daphnia pulicara = DPULI
Myotis lucifigus = MYOLU

```
                         *        20         *        40         *
AEDAE_PLT2   : RIPQFSRYLATGISFNALSYTFR--IGRSTAAEIVKETCLAVWDKLHELHM :  49
AEDAE_PLT1   : --SDYFSYLAHGPDIPFLSWSFK--IGESTSRGIIHEVCDVLWVELKGEFL :  47
ANOGA_PLT2a  : RLMITLRYLSTGIPFKSLSFTYC--IAHNTIGLIVYETCEAIWNTFNEEFI :  49
CIOIN_HARB1  : RLALTLRYLATGNSMRSLSFSFR--IGYSTCCEIIGETCEAIWSTLRPLYL :  49
DROWI_DPLT2  : RLALTLRFLATGDSYHSLMYLFR--IPVSTVAVIVPECCRAVYECLKREYL :  49
ANOGA_HARB1  : RLLITLRYLATGETFTSLQYVFR--VSRHSISRIVKETCACLIEALRSQHQ :  49
DROPE_DPLT3  : RIAIALYTLGSSSEYRTVGRLFG--VAPNSVCNILHEFCRALIDEFSKEYM :  49
DANRE_HARB1  : RVAICIWRLATNVEFRTISHLFG--IGQSTAVSITNCVASAIVKNLLSIFI :  49
DROPS_DPLT4  : QLASVMRYLATGCYQWAVAKDHHINIGRSTFGKILHKLIPLMDRLLCVEFI :  51
ANOGA_PLT3c  : KFAATLRFLAEGSYQTGVGNDFNIAIAQPTFSVIFTQCLNIIEQTFSAKWI :  51
DANRE_HARB2  : QLLAAVRYYATGSFLQVLGDGLG--LSKPSVSRAVQAVTYALLPL-AAEHI :  48
HOMSA_HARBI1 : QVLAALGFYTSGSFQTRMGDAIG--ISQASMSRCVANVTEALVER-ASQFI :  48
DANRE_HARB3  : QVLTTLGFLATGSFQRELADRSG--LSQSSLSRAMPAVWDGIIRM-SSRYI :  48
BOMMO_PLT1   : RVLIALRFYATGNFQVRAXATCEK-ISQSVVSKTVANISKRLALK-SRQFI :  49
TRICA_PLT1   : KILITMRYLATGSFQQLVGDTVA--VHKSTVCVVIKSVIQKIAQL-KPQFI :  48
ORYSA_PIF1   : KLGHFLYMISHNASYEDLQHEFH--HSGETIHRHIKAVFKVIPSLTYRFIK :  49
ARATH_PIF2   : MVATFLITVGQNSRYCHTMDTFK--RSKFSTSINFHKVLRALNMLAPTLMA :  49
DROYA_DPLT1  : KLIITLRFLATGASFASLAYSFK--IGRTTVSVVVKETVIALWEELQPLHM :  49
DROPE_DPLT1  : RLFVTLRYLSTGISMRALAFSFR--IAESTLRKIIPETCSAVWEELNSTHM :  49
DAPPU_PIF1   : QILAALNFYATGTFQKEVGHVLR--MSQSSVCRSVYDVSSALCSI-AREWI :  48

                     60         *        80         *       100
AEDAE_PLT2   : PQ-PTE-ELFKNVAKDYWEMWNFP-----NCIGSIDGKHIRIKCPP-NTGT :  92
AEDAE_PLT1   : PE-LTT-ADWTRNASQFYQLWNLP-----NCCGAVDGKHVKIECPP-NSGS :  90
ANOGA_PLT2a  : PF-PTT-SAFRNVEKEFRHKWNFP-----NCIGAIDGKHIRMKAPA-FSGT :  92
CIOIN_HARB1  : KT-PTSPQEWRLIASKFESNWNFP-----LCMGAIDGKHVMIQAPE-KQGS :  93
DROWI_DPLT2  : K--PNSTNEWLKISSDFESIWNFP-----HCIGALDGKHVVMKAPP-NSGS :  92
ANOGA_HARB1  : RL-PSTEEEWLAISRRFEQRWRFP-----HAIGAIDGKHVEIICPR-NSGS :  93
DROPE_DPLT3  : SPNYLTSDKIDECVKGF-EAIGFP-----QCLGAIDGCHIEIKPPA-AEAV :  93
DANRE_HARB1  : RT--PSEQEFESIIQGFRDKWGFP-----QCGGAIDGTHIGILAPP-VSSA :  92
DROPS_DPLT4  : SL-QMNNHQLQQSYEYFYRNFKLP-----RIGACVDGTHIRILKPV-QNHF :  95
ANOGA_PLT3c  : NL-EMEPGQQQEARRYFFGKSGIP-----GVVMCADGTHIKIIAPQ-NDRD :  95
DANRE_HARB2  : KF-PASRQAMSDIQEYFLTHYHIP-----QVIGVIDGTLIPISTPS-VDGH :  92
HOMSA_HARBI1 : RF-PADEASIQALKDEFYGLAGMP-----GVMGVVDCIHVAIKAPN-AEDL :  92
DANRE_HARB3  : RF-PYHAVDQPNIKAQFAAIAGFP-----NVIGAIDCTHIAIKAPS-EDEF :  92
BOMMO_PLT1   : KF-PALNE-RVETKRKFYRIAGFP-----GVIGCIDCTHIPIKNPSRARGE :  93
TRICA_PLT1   : KM-PNREE-LHNVQLKFYRKRRMP-----RVIGAIDCSHVRIESPGGPNAE :  92
ORYSA_PIF1   : QTTRVETHWKISTDQLFFPYF-------QNCLGAIDGTHVPITISQ-DLQA :  92
ARATH_PIF2   : KVTNTVPS-KISKTTRFYPYFKVHIFLCEDCVGAIDGTHINAMVQG-PEKA :  98
DROYA_DPLT1  : PQ-PTK-EIISQTADKFWNLWNFP-----NCAGAIDGKHIRIKCPA-DSGS :  92
DROPE_DPLT1  : IP-PDC-TEYKKIAHDFYEKSSFP-----NCIGAIDGKHSRIKCPK-NSGS :  92
DAPPU_PIF1   : SF----------VCLEFSEFAGFR-----GIIGAIDGCHIKIARPW-VDEK :  83
```

```
                         *        120         *        140         *
AEDAE_PLT2    : MFFNYKKFFSIV-LQAVADAKCKFIAIEVGSYGKQSDGGIFKSSTLFKLMK : 142
AEDAE_PLT1    : QFFNYKGDHSIN-LMAVCDANYKFLSVDVGAYGGHSDGGVFASSEFGKRLF : 140
ANOGA_PLT2a   : QYYNYKKYFSLH-LQAVADVNWKFIAIDVGEYGSRSDSGVFNSSSLFELIR : 142
CIOIN_HARB1   : EYYNYKGYHSIILLVALCDAEYCFTAVDIGDSGRHSDGGAFSNSQFGKCFT : 144
DROWI_DPLT2   : TYFNYKGTHSVV-LMVLADANYKIIYFDVGGKGRISDGRIFNASSLSYELQ : 142
ANOGA_HARB1   : EYHNYQKFFSIV-LMVVVDADYNFLWADAGGKGGISDGGIFKNTRLYHKLE : 143
DROPE_DPLT3   : DHHNYKGWYSTV-LFALVDFRYRFTYVNIGSAGRCNDSMIYQKSSLAKHIE : 143
DANRE_HARB1   : DYYNRKGFYSVI-LQGVVDHRLMFWDINVGWPGKVHDARVFANSSLFDRGQ : 142
DROPS_DPLT4   : VFYNRKGFYSMN-AMVVCNYNMEIIAIDATHPGSCHDSFIWNHSPAREYLS : 145
ANOGA_PLT3c   : QHYNRKGFYSLN-ALIVCDHKLTVRFVNAKFSGANHDSHIWNVCGIDTFFA : 145
DANRE_HARB2   : TYICRKGYPAIN-CQVICDHNCLITDIVARWPGSTHDSYIFTNSSVGQEAQ : 142
HOMSA_HARBI1  : SYVNRKGLHSLN-CLMVCDIRGTLMTVETNWPGSLQDCAVLQQSSLSSQFE : 142
DANRE_HARB3   : AYVNRKHFHSIN-VQIICDAQMRLTNIVARWPGSTHDSFILTNSMVGMRLQ : 142
BOMMO_PLT1    : IFRNRKGWFSIN-VQIICGPQMEIYDIVVRWPGSVHDSRIFNNSRCCLRFE : 143
TRICA_PLT1    : IFRNRKGFFSIN-VQAVCDADLQIRNIVARWPGSVHDSTIFNDSSLCAHLE : 142
ORYSA_PIF1    : PYRNRKGTLSQN-VMLVCDFDLNFLFIPSGWEGSATDARVLRSAMLK-GFN : 141
ARATH_PIF2    : SYRNRKGVISQN-VLAACNFDLEFIYVLSGWEGSAHDSKVLQDALTR-RTN : 147
DROYA_DPLT1   : MFYNYKKYFSIV-LQAVADANCKFIAIEVGGYGKQSDGGTFNASQLYMMLK : 142
DROPE_DPLT1   : DYFNYKKFFSMV-LQGVADSNCKFIFIELGFKGSQSDGEIFAASRLQQAII : 142
DAPPU_PIF1    : IYVNRKNFHSIN---AICDANGRALSVYAKKPGSTNDAAMFVESIIGQRLA : 131

                   160         *       180         *       200
AEDAE_PLT2    : SGE--LDIPPDCHLPG-TNISVPHVLVADEAYPLLPHVLRPYA-------- : 182
AEDAE_PLT1    : DGS--LNLPPAACLPN-STIEIPHYIVGDAAFPLKPNLMRPFP-------- : 180
ANOGA_PLT2a   : SNR--LNIPPPKPLPG-TTQRMPHVFIGDQGYPLKPFLLRPFP-------- : 182
CIOIN_HARB1   : QKENVLCVPQDDLLPG-SSIRVPYCIVGDAAFPLRKNIMRPYP-------- : 186
DROWI_DPLT2   : NEQ--LNVPGEQPLPG-RELKVPYVLVADDAFALKRYIMKPYP-------- : 182
ANOGA_HARB1   : NDQ--LNIPPATPLQVPYQTPVPYFILGDKAFAFTNYCLRPYS-------- : 184
DROPE_DPLT3   : ASA--LLQEKAKEISG---VNVPVMLIGDSAFRFSKKLMKPYP-------- : 181
DANRE_HARB1   : GNS--LFPPNIERFGD---VDVPVMLLGDAAYPLMPWLMKPYP-------- : 180
DROPS_DPLT4   : -TT-------ING-----------FVLADSGYALESFVLTPY--------- : 168
ANOGA_PLT3c   : EKN-------QNGEAG-------YFVLADAAYPSKPWLITPK--------- : 173
DANRE_HARB2   : NS--------NGH----------WRLLGDSGYPLRPYLFTPV--------- : 166
HOMSA_HARBI1  : AGM-------HKD----------SWLLGDSSFFLRTWLMTPL--------- : 167
DANRE_HARB3   : GGR-------VRD----------GWLLGDRGYPLKTWLLTPL--------- : 167
BOMMO_PLT1    : EGD-------LV-----------GILVGDSGYAQTGFMYTPV--------- : 167
TRICA_PLT1    : RGE-------YEN----------GFLLGDSGYACRPFLLTPV--------- : 167
ORYSA_PIF1    : -------------------VPQGKYYLVDGGYANTPSFLAPYRGVRYHLKE : 173
ARATH_PIF2    : RLQ----------------VPEGKYYLADCGFPNRRNFLAPLRSTRYHLQD : 182
DROYA_DPLT1   : ARQ--F-LPPDSCLPG-TNTKMPYVFIGDEAYPLLGNLLRPYS-------- : 181
DROPE_DPLT1   : RNE--LDIPSEEVLPN-SDIKAPFVFIGDEAYPLTKYLMRPYP-------- : 182
DAPPU_PIF1    : RGD----------------FSPFHLIGDSGYACTPYLLTPY--------- : 156
```

```
                          *        220         *        240         *
AEDAE_PLT2    : ---RRDCS-PDEEYFNARFSRARKSVECSFGAINSKWRLLWKP---IETEV : 226
AEDAE_PLT1    : ---GKLLP-PIRENFNNRLSRARRTIENAFGILVARWRVLKTT---LVMLP : 224
ANOGA_PLT2a   : ---DSEDP-AKN-YFNHLLSMARRCVECAF-------------------- : 207
CIOIN_HARB1   : ---GRYLP-DDQQIFNYRLSRARRVVENAFGILSSRWRIFRRP---IIAEP : 230
DROWI_DPLT2   : ---FRNQP-APNRVFNYRLSRARRVVENVFGIMANRFRVLRKP---LELGP : 226
ANOGA_HARB1   : ---GVHPPDSMERTFNKMHSTCRMPVENSLGILANRWRVLKG----IQLQP : 228
DROPE_DPLT3   : ---FSTVASLEQRTFNYNLSKARRVVENAFGHLKARFRIIGKG---LDSHH : 226
DANRE_HARB1   : ---ENQLTTPAQSTFNNRLSKARMTVERAFGPLKGRWRCLMKR---CDCHI : 225
DROPS_DPLT4   : ---RSAEIATYQHRFNIKHTGARNIIERTIGVLKSRFRCLQ---RTLNYPP : 213
ANOGA_PLT3c   : ---RNPAPGSPDADYNTKHSQGREIVERTIGMIKNRFRCILGA-RQLHYTP : 220
DANRE_HARB2   : ---ANPVSNS-EAHFNEAHRVARSTVERTLGRWKLRFRAIHKSSGGLLFVP : 213
HOMSA_HARBI1  : ---HIPETPA-EYRYNMAHSATHSVIEKTFRTLCSRFRCLDGSKGALQYSP : 214
DANRE_HARB3   : ---NNPQTDQ-ERRYNDAHSHTRSVVERAIGQLKCRWRCLDKSGGVLLYRP : 214
BOMMO_PLT1    : ---LNPQTDP-EHRYNRAHLLTRNIIERVNGVLKRRFACLCRK---LQNST : 211
TRICA_PLT1    : ---LNPRTAA-EEAYNLSHRTTRNAIERCFGVLKRRFPCLSLG---LRTKM : 211
ORYSA_PIF1    : FGRGQQRPRNYKELFNHRHAILRNHIERAIGVLKKRFPILKVG---THHRI : 221
ARATH_PIF2    : FRGEGRDPTNQNELFNLRHASLRNVIERIFGIFKSRFLIFKSA---PPFSF : 230
DROYA_DPLT1   : ---RRDIN-ANNEYFNSRLSRARRCIECAFGIITSKWRLLWKP---IETDP : 225
DROPE_DPLT1   : ---RRSLT-EPRSVFNERLSSARRCVECAFGILTEKWRLMKRE---IDATP : 226
DAPPU_PIF1    : ----AAPSNEPERRFNVAHKKSRCVIERFFGMVKRRFPALFFG---IRMQP : 200

                   260         *
AEDAE_PLT2    : QTAETITKAICILHNVI : 243
AEDAE_PLT1    : KHAEKVVLASVILHNYL : 241
ANOGA_PLT2a   : ----------------- :   -
CIOIN_HARB1   : KKVIAITKACCCLHNFL : 247
DROWI_DPLT2   : EKTIDVVSAICALHNWL : 243
ANOGA_HARB1   : DVAKNIVLTTVYLHNFL : 245
DROPE_DPLT3   : KNNSAIIMSCCILHNIL : 243
DANRE_HARB1   : DNINSIISACCVLHNYC : 242
DROPS_DPLT4   : IFCCQIINVCCALHNIC : 230
ANOGA_PLT3c   : EKATQITNVCCILHNMC : 237
DANRE_HARB2   : QKCCAVITVTAMLHNIA : 230
HOMSA_HARBI1  : EKSSHIILACCVLHNIS : 231
DANRE_HARB3   : NKVCRIVLACGVLHNVA : 231
BOMMO_PLT1    : PNTCNIIVSCAVLHNIC : 228
TRICA_PLT1    : NTTLATIVACAVLHNIA : 228
ORYSA_PIF1    : KNQVKIPVATVVFHNLI : 238
ARATH_PIF2    : KTQAEIVLSCAALHNFL : 247
DROYA_DPLT1   : SFVDIIVKSICILHNTI : 242
DROPE_DPLT1   : KTAIILIKAMCLLHNII : 243
DAPPU_PIF1    : GRACRVIMACFILDNIA : 217
```

Supplemental Figure S2h. Alignment of Merlin family elements from multiple species, including one new family, Dappu_Merlin, from *Daphnia pulex*.

Species abbreviations

Drosophila melanogaster = DROME
Drosophila biofasciata = DROBI
Drosophila helvetica = DROHE
Drosophila willistoni = DROWI
Drosophila hydei = DROHY
Drosophila yakuba = DROYA
Drosophila persimilis = DROPE
Drosophila pseudoobscura = DROPS
Drosophila buzzati = DROBU
Drosophila ananassae = DROAN
Mus musculus = MUSMU
Danio rerio = DANRE
Arabidopsis thaliana = ARATH
Trichomonas vaginalis = TRIVA
Fusarium oxysporum = FUSOX
Podospora anserina = PODAN
Caenorhabditis briggsae = CAEBR
Caenorhabditis elegans = CAEEL
Caenorhabditis remanei = CAERE
Bombyx mori = BOMMO
Rana pipiens = RANPI
Salmo salar salar = SALSA
Oncorhynchus mykiss = ONCMY
Pleuronectes platessa = PLEPL
Anopheles albimanus = ANOAL
Anopheles gambiae = ANOGA
Haemonchus contortus = HAECO
Saccoglossus kowalevskii = SACKO
Ixodes scapularis = IXOSC
Halorubrum lacusprofundi = HALLA
Hahella chejuensis = HAHCH
Marinobacter aquaeolei = MARAQ
Acidithiobacillus caldus = ACICA
Aspergillus niger = ASPNI
Talaromyces stipitatus = TALST
Culex quinquefasciatus = CULQU
Tribolium castaneum = TRICA
Oryza sativa = ORYSA
Aaedes aegypti = AAEAE
Ciona intestinalis = CIOIN
Trichuris muris = TRIMU
Schistosoma japonicum = SCHJA
Schistosoma mansoni = SCHMA

Antirrhinum majus = ANTMA
Bactrocera dorsalis = BACDO
Phakopsora pachyrhizi = PHAPA
Oryzias latipes = ORYLA
Zea mays = ZEAMA
Tolypocladium inflatum = TOLIN
Strongylocentrotus purpuratus = STRPU
Nematostella vectensis = NEMVE
Hydra magnipapillata = HYDMA
Glyptapanteles indiensis = GLYIN
Acyrthosiphon pisum = ACYPI
Branchiostoma floridae = BRAFL
Candida albicans = CANAL
Chelonus inanitus bracorvirus = CHEIN
Cucumis melo = CUCME
Culex pipiens = CULPI
Ciona savigny = CIOSA
Yarrowia lipolytica = YARLI
Vitis vinifera = VITVI
Equus caballus = EQUCA
Entamoeba dispar = ENTDI
Entamoeba histolytica = ENTHI
Entamoeba invadens = ENTIN
Entamoeba moshkovskii = ENTMO
Glypta fumiferanae ichnovirus = GLYFU
Helobdella robusta = HELRO
Staphylococcus epidermidis = STAEP
Meloidogyne incognita = MELIN
Schmidtea mediterranea = SCHME
Mycobacterium smegmatis = MYCSM
Nassonia vitripennis = NASVI
Phytopthora infestans = PHYIN
Phytopthora sojae = PHYSO
Trichinella spiralis = TRISP
Penicillium chrysogenum = PENCH
Medicago truncatula = MEDTR
Deinococcus radiodurans = DEIRA
Monodelphis domestica = MONDO
Bos taurus = BOSTA
Haemonchus contortus = HAECO
Daphnia pulicara = DPULI
Myotis lucifigus = MYOLU

```
                                            *        20         *        4
gi|198411948|_CIOIN      : WCGFLREVCSEDLIRNT---APIGGPG--QIVAIDETHV :  34
gi|160774047|_DANRE      : ICVAALQRLRRRTG------LRMGGKSRRKFISIDESKF :  33
gi|157749299|_CAEBR      : WCQWFRDIMAESLYQPA---IMIGGVG--ETVQIDETNI :  34
gi|19671605|_ANOGA       : WFKILSEISAEYVETHQ---QQIGGEG--LTVGINESVI :  34
gi|13592884|_TRIMU       : WNESMRGVAAEVLMRQP---LVIWGPG--LTVEVDETVY :  34
gi|28346737|_SCHJA       : WHQPCRDICNEMMSRLN---LSQGGVG--NIIEIDGTAI :  34
gi|15809179|Merl_HOMSA   : -YNFYSKVCKYYFELHL---IQIGGPG--PYLQIDKYCF :  33
gi|52548230|Merlin_SCHMA : WYEFCRDICATKLTSIH---QLYGGVG--KIVEIDETVV :  34
DAPPU_Merlin             : WTKYLREVVFHLVFGLSAQFTQIGGVG--KVVEIDESLF :  37

                           0         *        60         *
gi|198411948|_CIOIN      : ARRKP---GNAQARPVRAQWVFGGICIGTGE--CFMRLV :  68
gi|160774047|_DANRE      : -----YNRGRFRSTWRRNSWVFGMLEIKGQRRLPVLKIV :  67
gi|157749299|_CAEBR      : -VKRKYNVGRIV----RNGWLIGGIQNNTRA--VFIEIV :  66
gi|19671605|_ANOGA       : -TKRKYHRGRIAD--NNQVWLVGGICRETKE--IFLELV :  68
gi|13592884|_TRIMU       : -SKRKYQRGRLY-----PQQVFGGICRETKE--CFLVRV :  65
gi|28346737|_SCHJA       : -KNRKYSRQRVC----KTEWVPGIYYETLVK--GYFQYI :  66
gi|15809179|Merl_HOMSA   : SHKLNYYHGHVL---EREIWGFGIINTTRQLAVGYLTIL :  69
gi|52548230|Merlin_SCHMA : -RKRKYNRGRCI----KEDWVFGIYDRSLQK--GHFQRV :  66
DAPPU_Merlin             : GKKRKYKRGNCK----KGEWVFGGVERGTNC--CFLVIV :  70

                           80         *        100         *
gi|198411948|_CIOIN      : DTRDAATLLPIIADCIAPNSTIYSDEWRAYA-GISAMPQ : 106
gi|160774047|_DANRE      : KNRSRQTLVPIIRRHARRGSTIFSDCWRAYAQALPRH-- : 104
gi|157749299|_CAEBR      : DKRDQATCERIIQQYVAPGTTVITDCWRGYN-GLAAL-- : 102
gi|19671605|_ANOGA       : QKRDAGNLQGIIMNNVAPGTTIVTDGWRAYI-GLDGK-- : 104
gi|13592884|_TRIMU       : PDRSSRTLIPLIQQYVRPGTMVITDCWRGYD-SLSQV-- : 101
gi|28346737|_SCHJA       : PNTPR-TLIPIIQLYVIPRSTIYTDDWRAYR-PLHRL-- : 101
gi|15809179|Merl_HOMSA   : PN-----FAAYFVCIVQPGSPI-SDLWVSHS-NIQSL-- :  99
gi|52548230|Merlin_SCHMA : RNRQAPTIIPIIQQYVLPGTTVYTDDWRAYR-CLGRL-- : 102
DAPPU_Merlin             : PDRKRKTLLAIIKQYIAPGTTIISDEWRAYK-CL-RH-- : 105

                           120        *        140         *
gi|198411948|_CIOIN      : NYTHKTVNH---SYQFVSESGV--HPNHVENLWRNCKRE : 140
gi|160774047|_DANRE      : GYRHFTVNH---SENFVNPYTGC-HTQHIERAWQSIKSQ : 139
gi|157749299|_CAEBR      : GYDHKTVNH---SQNFVDPATGL-HTQRVESLWSHLKRR : 137
gi|19671605|_ANOGA       : GYEHEMINH---SENFVDPSDPLVHTQTIENLWRWVKPF : 140
gi|13592884|_TRIMU       : GYTHQRVNH----CQFREPCRRGAH------------- : 122
gi|28346737|_SCHJA       : GYFHHVVFY---KRYLVDSSIDV-HKQNIKGYWSHLKEF : 136
gi|15809179|Merl_HOMSA   : ICFHDQSIHNDPSFHFISPLDV--HTPNIKSYWKKYKSN : 136
gi|52548230|Merlin_SCHMA : GYVHEVVIH---KRHFVDPTTGV-HTNNIEAMWSRLKEF : 137
DAPPU_Merlin             : GCIHLTVNH---SKHFKDPITGA-HTNTVEGMWAHAKRT : 140
```

```
gi|198411948|_CIOIN       : LAK : 143
gi|160774047|_DANRE       : VNK : 142
gi|157749299|_CAEBR       : IKP : 140
gi|19671605|_ANOGA        : LRS : 143
gi|13592884|_TRIMU        : --- :   -
gi|28346737|_SCHJA        : RKP : 139
gi|15809179|Merl_HOMSA    : CKT : 139
gi|52548230|Merlin_SCHMA : LRP : 140
DAPPU_Merlin              : LIK : 143
```

Supplemental Figure S2i. Alignment of CACTA family elements from multiple species, including three new families, Dappu_CacataA1.1, Dappu_CacataA3.1, and Dappu_CacataA4.1 from *Daphnia pulex*.

Species abbreviations

Drosophila melanogaster = DROME
Drosophila biofasciata = DROBI
Drosophila helvetica = DROHE
Drosophila willistoni = DROWI
Drosophila hydei = DROHY
Drosophila yakuba = DROYA
Drosophila persimilis = DROPE
Drosophila pseudoobscura = DROPS
Drosophila buzzati = DROBU
Drosophila ananassae = DROAN
Mus musculus = MUSMU
Danio rerio = DANRE
Arabidopsis thaliana = ARATH
Trichomonas vaginalis = TRIVA
Fusarium oxysporum = FUSOX
Podospora anserina = PODAN
Caenorhabditis briggsae = CAEBR
Caenorhabditis elegans = CAEEL
Caenorhabditis remanei = CAERE
Bombyx mori = BOMMO
Rana pipiens = RANPI
Salmo salar salar = SALSA
Oncorhynchus mykiss = ONCMY
Pleuronectes platessa = PLEPL
Anopheles albimanus = ANOAL
Anopheles gambiae = ANOGA
Haemonchus contortus = HAECO
Saccoglossus kowalevskii = SACKO
Ixodes scapularis = IXOSC
Halorubrum lacusprofundi = HALLA
Hahella chejuensis = HAHCH
Marinobacter aquaeolei = MARAQ
Acidithiobacillus caldus = ACICA
Aspergillus niger = ASPNI
Talaromyces stipitatus = TALST
Culex quinquefasciatus = CULQU
Tribolium castaneum = TRICA
Oryza sativa = ORYSA
Aaedes aegypti = AAEAE
Ciona intestinalis = CIOIN
Trichuris muris = TRIMU
Schistosoma japonicum = SCHJA
Schistosoma mansoni = SCHMA
Antirrhinum majus = ANTMA
Bactrocera dorsalis = BACDO
Phakopsora pachyrhizi = PHAPA
Oryzias latipes = ORYLA
Zea mays = ZEAMA
Tolypocladium inflatum = TOLIN
Strongylocentrotus purpuratus = STRPU
Nematostella vectensis = NEMVE
Hydra magnipapillata = HYDMA
Glyptapanteles indiensis = GLYIN
Acyrthosiphon pisum = ACYPI
Branchiostoma floridae = BRAFL
Candida albicans = CANAL
Chelonus inanitus bracorvirus = CHEIN
Cucumis melo = CUCME
Culex pipiens = CULPI
Ciona savigny = CIOSA
Yarrowia lipolytica = YARLI
Vitis vinifera = VITVI
Equus caballus = EQUCA
Entamoeba dispar = ENTDI
Entamoeba histolytica = ENTHI
Entamoeba invadens = ENTIN
Entamoeba moshkovskii = ENTMO
Glypta fumiferanae ichnovirus = GLYFU
Helobdella robusta = HELRO
Staphylococcus epidermidis = STAEP
Meloidogyne incognita = MELIN
Schmidtea mediterranea = SCHME
Mycobacterium smegmatis = MYCSM
Nassonia vitripennis = NASVI
Phytopthora infestans = PHYIN
Phytopthora sojae = PHYSO
Trichinella spiralis = TRISP
Penicillium chrysogenum = PENCH
Medicago truncatula = MEDTR
Deinococcus radiodurans = DEIRA
Monodelphis domestica = MONDO
Bos taurus = BOSTA
Haemonchus contortus = HAECO
Daphnia pulicara = DPULI
Myotis lucifigus = MYOLU

```
                                     *        20          *         40
gi|117644083|SCHMA : YHLGLKTNLLRYVELWLCTCDF---DSLQLYINVDGLSMSRSSSQ :  42
gi|118026895|STRPU : YHFGVADGINRISEICIATKDA---TRINLQINIDGLPLFKSSKT :  42
gi|118026885|DROPS : LHIGLKTQLSKL--FYILKLE----ESLCFDINIDGLPLYKSSNA :  39
gi|122091425|TRICA : VYFGITPTLLYLQHNLVKECISLQNNILQVSFNIDGIPLFNSSNK :  45
gi|118026879|AEDAE : WHNGLESCLKRIFRDMS-TSMT-----VSINVNVDGLPIYNSTTK :  39
gi|198413725|CIOIN : YYFNILHQIVSIVHMYSTAKETNNLDVLDLSINIDGLPVFSSTKS :  45
gi|118026887|DANRE : IHLSLAEQIKKALKRYP-SEITSTIHVLEISFNVDGLPLFKSTSL :  44
DAPPU_CactaA1.1    : HHFSLKRGLLAKLKKGMINKESRVM---KIQINIDGTQIFKTNSL :  42
DAPPU_CactaA3.1    : FHFGLI--KYLFEFLNSLSPTQIPLCI-ELCVNVDGIPLYKSSRS :  42
DAPPU_CactaA4.1    : QHFGLIKGLLLKLKSGIIDGRNGI----KLQFNIDGSNLYKSGTK :  41

                         *        60          *         80          *
gi|117644083|SCHMA : HLWPVLGRIVAPRLSDV--FMIGIYGGNTKPA-QFNEISADTISE :  84
gi|118026895|STRPU : QFWPILGRISMPFESET--FIIGLYCGESKPH-SVQHYLTQFVSE :  84
gi|118026885|DROPS : QLWPILIRLVNVKNAPI--LPIGIFLGKSKPT-CCDEFLRKFTCE :  81
gi|122091425|TRICA : QFWPILCRV-HLNQVKIKPFPVALFYGDSKPF-SIFEYLNEFIKE :  88
gi|118026879|AEDAE : NFWPILCNIYEYPSIAP--FTVGIYYGNGKPK-DINQFMSPFVEE :  81
gi|198413725|CIOIN : SFWPVLACITNITPQIV--FPVAICYGASKPS-N-LDFLSDTIEH :  86
gi|118026887|DANRE : SLWPVLCCI-HLEPVTV--FPVTLTLGPQRPL-D-LKFLEDAVTE :  84
DAPPU_CactaA1.1    : DLWPILVRVTNSLDALP--FVVSLFIGKGKPT-NIEDFLKPFLEE :  84
DAPPU_CactaA3.1    : QFWPILGLLRNIDGAKP--FVIGIFEGTSKPL-DVNLFLEDFVRE :  84
DAPPU_CactaA4.1    : AFWPILCRVSNANDSRP--FPVSIFCGDGEKPPDLNLYLEPFLNE :  84

                               100         *        120         *
gi|117644083|SCHMA : IKEMTETGLLSVRFNKYIAIKLSAVICDAPARSDVRYTVNHNGKA : 129
gi|118026895|STRPU : MKGLEESGGVMIKSTNDIPISIACFICDAPARAFVKQTKPHNAYY : 129
gi|118026885|DROPS : LQDLLQNG-VELG--NKIKISIRAIVCDAPARAFISGTPGHTSSH : 123
gi|122091425|TRICA : INQLTSEG-LQINE-FRFEIRVMCITCDAPARSYVKGIKGHNAYF : 131
gi|118026879|AEDAE : LVNLLRLG-VTING-YQLNLRIRCFICDTPARSFVKAVISFNGKY : 124
gi|198413725|CIOIN : ILLAINEG-LEVHG-KFYSVRLQSIVCDAPARSFVKATKLFSGYH : 129
gi|118026887|DANRE : LKQLFESS-LTFDG--NIQVKLRCIVCDAPAKAMVKSIKQFSAYY : 126
DAPPU_CactaA1.1    : LIALQTEG-LQFEDIS-YSVEVSSFVCDAPARAFLKVITGHTGYF : 127
DAPPU_CactaA3.1    : --VKFLETNVILNGK--IPFKISAILCDAPARSFLTDVKGHTAYF : 125
DAPPU_CactaA4.1    : LKPLEENG-MDVNDRH-LVVKSIAFICDAPARSFVKGIIGHTGKY : 127

                        140          *        160          *        180
gi|117644083|SCHMA : GCDRCVVNGRR--LDGKMTFPNG---EYT-LRTDD-SFRNQTQYI : 167
gi|118026895|STRPU : GCDKCVQKGD---WSNKVIFPET---DSP-LRTD-SAFKNMVQKE : 166
gi|118026885|DROPS : GCSKCTQVARK--LNGTLTF-KT---DCG-ILITDNDFTNRIYPE : 161
gi|122091425|TRICA : GCETCIQKGVN--LNRRIIYIET---NSH--PRTHQNFVSKEYPA : 169
gi|118026879|AEDAE : GCLKCTTKGRYSMLTRTMTYPEL---TAP-LRTDE-KFRSMEYSN : 164
gi|198413725|CIOIN : GCDKCMQRGL---WCGRMTYPEV---ENFIERTDD-SFRRQTNIQ : 167
gi|118026887|DANRE : GCDRCEQKGF---WEKRMTYQEV---DAFELRTDC-SFRVQKQPE : 164
DAPPU_CactaA1.1    : GCERCNQKGVYD-LYYRCTTFPQ-LTDCV-LRTD-ASFRAKLQRQ : 168
DAPPU_CactaA3.1    : GCPKCETKGKYA-KNHNSKKGRVTFPTSNARLRNNHSFRTRRQAE : 169
DAPPU_CactaA4.1    : ACERCTVIGETV--NNHMTFTAM---SSRPRTNDS--FRSGRDRR : 165
```

```
                                           *         200          *
gi|117644083|SCHMA : HHKGH--LFESLSIDMILTFP------LDPMHMVYLG : 196
gi|118026895|STRPU : HHNNKSPISE-LSVGLVSQFP------LDPMHLVHLG : 196
gi|118026885|DROPS : QHSQKY-LTKMTALESV-NVKMVTQIPLDCMHLIDLG : 196
gi|122091425|TRICA : HHLFDTPLKNMLNIDLIKQVT------LDYMHLCCLG : 200
gi|118026879|AEDAE : HQRGQTPLMQ-LPIDMIQDII-----VGDSLHLLELG : 195
gi|198413725|CIOIN : HHHSVSPFCS-LPIDMIKAFP------IDYMHQSCLG : 197
gi|118026887|DANRE : HHNGTTPFAE-LPIDMIGQFP------IDYMHQACLG : 194
DAPPU_CactaA1.1    : HHKGSSPLLD-LKIDMISCIP------LDYMHLVLLG : 198
DAPPU_CactaA3.1    : HHKGRSLLEE-LNLDMVLNIP------LDSMHLCDLG : 199
DAPPU_CactaA4.1    : HHNEPTPLLR-LRMDIVKCFP------IDYMHLTCLG : 195
```

Supplemental Figure S2j. Alignment of Maverick family elements from multiple species, including one new family, Mav_2.1_Dappu, from *Daphnia pulex*.

Species abbreviations

Drosophila melanogaster = DROME
Drosophila biofasciata = DROBI
Drosophila helvetica = DROHE
Drosophila willistoni = DROWI
Drosophila hydei = DROHY
Drosophila yakuba = DROYA
Drosophila persimilis = DROPE
Drosophila pseudoobscura = DROPS
Drosophila buzzati = DROBU
Drosophila ananassae = DROAN
Mus musculus = MUSMU
Danio rerio = DANRE
Arabidopsis thaliana = ARATH
Trichomonas vaginalis = TRIVA
Fusarium oxysporum = FUSOX
Podospora anserina = PODAN
Caenorhabditis briggsae = CAEBR
Caenorhabditis elegans = CAEEL
Caenorhabditis remanei = CAERE
Bombyx mori = BOMMO
Rana pipiens = RANPI
Salmo salar salar = SALSA
Oncorhynchus mykiss = ONCMY
Pleuronectes platessa = PLEPL
Anopheles albimanus = ANOAL
Anopheles gambiae = ANOGA
Haemonchus contortus = HAECO
Saccoglossus kowalevskii = SACKO
Ixodes scapularis = IXOSC
Halorubrum lacusprofundi = HALLA
Hahella chejuensis = HAHCH
Marinobacter aquaeolei = MARAQ
Acidithiobacillus caldus = ACICA
Aspergillus niger = ASPNI
Talaromyces stipitatus = TALST
Culex quinquefasciatus = CULQU
Tribolium castaneum = TRICA
Oryza sativa = ORYSA
Aaedes aegypti = AAEAE
Ciona intestinalis = CIOIN
Trichuris muris = TRIMU
Schistosoma japonicum = SCHJA
Schistosoma mansoni = SCHMA

Antirrhinum majus = ANTMA
Bactrocera dorsalis = BACDO
Phakopsora pachyrhizi = PHAPA
Oryzias latipes = ORYLA
Zea mays = ZEAMA
Tolypocladium inflatum = TOLIN
Strongylocentrotus purpuratus = STRPU
Nematostella vectensis = NEMVE
Hydra magnipapillata = HYDMA
Glyptapanteles indiensis = GLYIN
Acyrthosiphon pisum = ACYPI
Branchiostoma floridae = BRAFL
Candida albicans = CANAL
Chelonus inanitus bracorvirus = CHEIN
Cucumis melo = CUCME
Culex pipiens = CULPI
Ciona savigny = CIOSA
Yarrowia lipolytica = YARLI
Vitis vinifera = VITVI
Equus caballus = EQUCA
Entamoeba dispar = ENTDI
Entamoeba histolytica = ENTHI
Entamoeba invadens = ENTIN
Entamoeba moshkovskii = ENTMO
Glypta fumiferanae ichnovirus = GLYFU
Helobdella robusta = HELRO
Staphylococcus epidermidis = STAEP
Meloidogyne incognita = MELIN
Schmidtea mediterranea = SCHME
Mycobacterium smegmatis = MYCSM
Nassonia vitripennis = NASVI
Phytopthora infestans = PHYIN
Phytopthora sojae = PHYSO
Trichinella spiralis = TRISP
Penicillium chrysogenum = PENCH
Medicago truncatula = MEDTR
Deinococcus radiodurans = DEIRA
Monodelphis domestica = MONDO
Bos taurus = BOSTA
Haemonchus contortus = HAECO
Daphnia pulicara = DPULI
Myotis lucifigus = MYOLU

```
                                       *        20        *
gi|13924|ZEAMA              : RRGS--SMVVYFHNLSQFDGIMIL------SFLTKSY- :  29
gi|156398391|NEMVE          : FRAP-KFFPVLFHNLSGYDSHLFV------KNLGTSE- :  30
gi|152079074|Mav_Cr1.1_CA : KRKN-NFIPVFFHNLKGYDSHLIINDEKSAEFLVDKG- :  36
Mav_2.1_DAPPU               : KRKNQRFLKIFMHNGSRYDFHFLV------KALAKKDI :  32

                              40         *       60        *
gi|13924|ZEAMA              : KNCHIEPIMRNDCIYSIKLYKVSKNG-------DKRLV :  60
gi|156398391|NEMVE          : GKINCIPNNEEKYISFTKQVVVDRFTNKEGKQVDVKRD :  68
gi|152079074|Mav_Cr1.1_CA : VTIKNISANIEKFISFSYHFEGEEFS-RNGKFFTKKYE :  73
Mav_2.1_DAPPU               : KNLYVLPYNMENF----RMIKFNSFM----------- :  54

                                80        *        100        *
gi|13924|ZEAMA              : LTFMDSYLLLKVKLADLAD-------------SFCPE :  84
gi|156398391|NEMVE          : IRFIDSFKFMSASLDSLVK----------NLPRESFKN :  96
gi|152079074|Mav_Cr1.1_CA : IRFLDSFGFMACSLDHLSS----------LLKTEQCAI : 101
Mav_2.1_DAPPU               : --LLDSLAFLQSSLSQLADELKESNHDYPILKQSHIVK :  90

                                 120        *       140        *
gi|13924|ZEAMA              : LGG-------------KGSFDHQNVTV------DKLPS : 103
gi|156398391|NEMVE          : LTIHYKGEQ-LQLLLRKGVFPYDWFCNFDQLDAIQLPP : 133
gi|152079074|Mav_Cr1.1_CA : TKQYYNNEDTFKLMKRKGVYPYDFIDSFEKYSNTELPS : 139
Mav_2.1_DAPPU               : TRGYFDNEK-FEMVLQKGFFPYEYWVKF-----YYLPQ : 122

                                   160        *       180        *
gi|13924|ZEAMA              : IREDSLTYLKQDILITAAVMQRAKAIIWEEYGIDILKV : 141
gi|156398391|NEMVE          : K--EAFYSTLNDTDISEEDYQHAQK-VWETFKMSTMRD : 168
gi|152079074|Mav_Cr1.1_CA : I--ESFYNTLTDENISNESFEYAQK-VWKETNCETLKD : 174
Mav_2.1_DAPPU               : I--STLYCFTTIIGVS----------IGRRTRIAI--- : 145

                                     200        *       220
gi|13924|ZEAMA              : LT---------ISALALKIFRRV-----------YYKD : 159
gi|156398391|NEMVE          : YHDLYLESDVLLLADVFENFRDVCLENYGLDPAWYYTA : 206
gi|152079074|Mav_Cr1.1_CA : YTEKYMINDVLLLADVFESFRKVSLEKYHLDPCWYYTS : 212
Mav_2.1_DAPPU               : -----------------------------LSPAWIGRS : 154

                               *        240        *       260
gi|13924|ZEAMA              : -----------DDDNRIYIPDDNEAQFIREGYYGG--- : 183
gi|156398391|NEMVE          : PGLAWDAALKITRVELELLTDCDMLLMFEEGIRGGVSM : 244
gi|152079074|Mav_Cr1.1_CA : PGLAWDAMLLKTDVKLQTIKDVEMYNFIEKGIRGGMCN : 250
Mav_2.1_DAPPU               : RGV--------------------INQFVTSMRMENCE : 171

                                 *        280        *        300
gi|13924|ZEAMA              : ----HTDVYKPYGEN---------LYYYDVNSLYPSSM : 208
gi|156398391|NEMVE          : ISTRHSKANNPYMREYDPNLPTKYITYLDANNLYGWAM : 282
gi|152079074|Mav_Cr1.1_CA : AMLRHSKANNKYMPDYNLEEESKYLLYLDANNLYGWAM : 288
Mav_2.1_DAPPU               : RMCLPEKILYDYVYK---------LIFLFFQNLYGLSQ : 200
```

```
                                      *       320         *       340
gi|13924|ZEAMA               : LDDMPIGKTRWVSDLGSKKSKIVLNDMFG----FIRAF : 242
gi|156398391|NEMVE           : SKPLPTHGFRWMTDQELKE--------WGRHPCVVEVD : 312
gi|152079074|Mav_Cr1.1_CA    : SQKLPYDEFEFVENFELEM--IDDLTANG-KGCILEVD : 323
Mav_2.1_DAPPU                : IGKLPLSDFEWVPDYHLTSFDVDDIDLDGKKGYILEVD : 238

                                        *       360         *       380
gi|13924|ZEAMA               : IICPKHIKK-----PLLPYKKDDG-------------- : 261
gi|156398391|NEMVE           : MAYPRHLHDSHDDYPLAPESIKIN-------------- : 336
gi|152079074|Mav_Cr1.1_CA    : LDYPKELHDKHNDLPFCPENKRVG-------------- : 347
Mav_2.1_DAPPU                : LEYPEELHYHHNDFPLAPESYNIELDDLSKYSKECYLK : 276

                                          *       400         *       4
gi|13924|ZEAMA               : ---TIIFPTGRFLGVYFSEELKYAV---------SLGY : 287
gi|156398391|NEMVE           : -------KVGKLIPN-LNDKTKYVVHHETLKLYESLGL : 366
gi|152079074|Mav_Cr1.1_CA    : -------TSNKLISD-FSPKRNYVIHYKMLQQVLDHGL : 377
Mav_2.1_DAPPU                : SNQNVTYKSTKLTAT-FLDRLNYVVHIKNLKLYLDLGL : 313

                               20         *       440         *
gi|13924|ZEAMA               : KVYPICGYIFDRKESPFKRFVYDIYSKRLDAKAKGEKA : 325
gi|156398391|NEMVE           : KVTKVHRGITFEESGWLKTYIDLNTSLRAKATNDFEKD : 404
gi|152079074|Mav_Cr1.1_CA    : MLKKIHRVVTFKESNWLSSYIELNTKLRTTAENDFEKD : 415
Mav_2.1_DAPPU                : KLKKIHRILMFNQESFLEPFITKCTEERRNAKTVFEKN : 351

                                 460         *       480         *
gi|13924|ZEAMA               : LDFIYKITMNSLYGR-FGISPESTTTQIVSTEESRKLA : 362
gi|156398391|NEMVE           : F---FKLMNNSVFGKTMENIRNRVDIRLITNEKQARKL : 439
gi|152079074|Mav_Cr1.1_CA    : F---FKLMNNSVFGKTMENVRSRVDVKVLSDMDKVMKL : 450
Mav_2.1_DAPPU                : Q---FKKISNSCYGKTIENVRDYITVKIHINEKSFKKA : 386

                                  500         *       520         *
gi|13924|ZEAMA               : LYNDGFVQSYELSSDKCLVTCKNVRSLDLLKLSSDRPT : 400
gi|156398391|NEMVE           : ISKPNY-KHRTIFCENLAAI-----HMRKTRLVFNKPV : 471
gi|152079074|Mav_Cr1.1_CA    : AGSNNF-KQRHIINDNMILV-----EMTQKSIKLDKPI : 482
Mav_2.1_DAPPU                : ISKHTF-KSFSIIDENLIIT-----SHKLPEILHSKPY : 418

                                    540         *       560         *
gi|13924|ZEAMA               : YAAVQISAAV-TGYARIRMHPFISRDDC-------YYT : 430
gi|156398391|NEMVE           : YLGMCILDL--SKNLMYDFHYGYVKPKYGDK-AKLLFT : 506
gi|152079074|Mav_Cr1.1_CA    : YVGMSILDL--SKYLMYEFHYDVMLPKYGNN-LKLCYQ : 517
Mav_2.1_DAPPU                : AVGFTILEYVSKKYVLVMPRFELGSADYKTGMLTITPH : 456


gi|13924|ZEAMA               : DTDSV : 435
gi|156398391|NEMVE           : DTDSL : 511
gi|152079074|Mav_Cr1.1_CA    : DTDSF : 522
Mav_2.1_DAPPU                : DLRNI : 461
```
