# Supplementary material for: DNA transposons and the role of recombination in mutation accumulation in Daphnia pulex
Source: Genome Biol. 2010 Apr 30;11(4):R46. doi: 10.1186/gb-2010-11-4-r46 (PMC2884549; doi:10.1186/gb-2010-11-4-r46)
Supplement: Additional file 5 — Trace file from transposon display reactions showing evidence for a putative germline gain of a copy of the hATA1.1 element. The top four traces show separate runs for the sample, indicating a new, replicable peak is found at 385 bp (red box). A putative somatic insertion is also visible in the top trace file (at 436 bp; blue box) where a new peak was observed in only this replicate. The bottom two traces are from another line and represent the ancestral state for the lineages lacking these new copies. [file gb-2010-11-4-r46-S5.pdf]

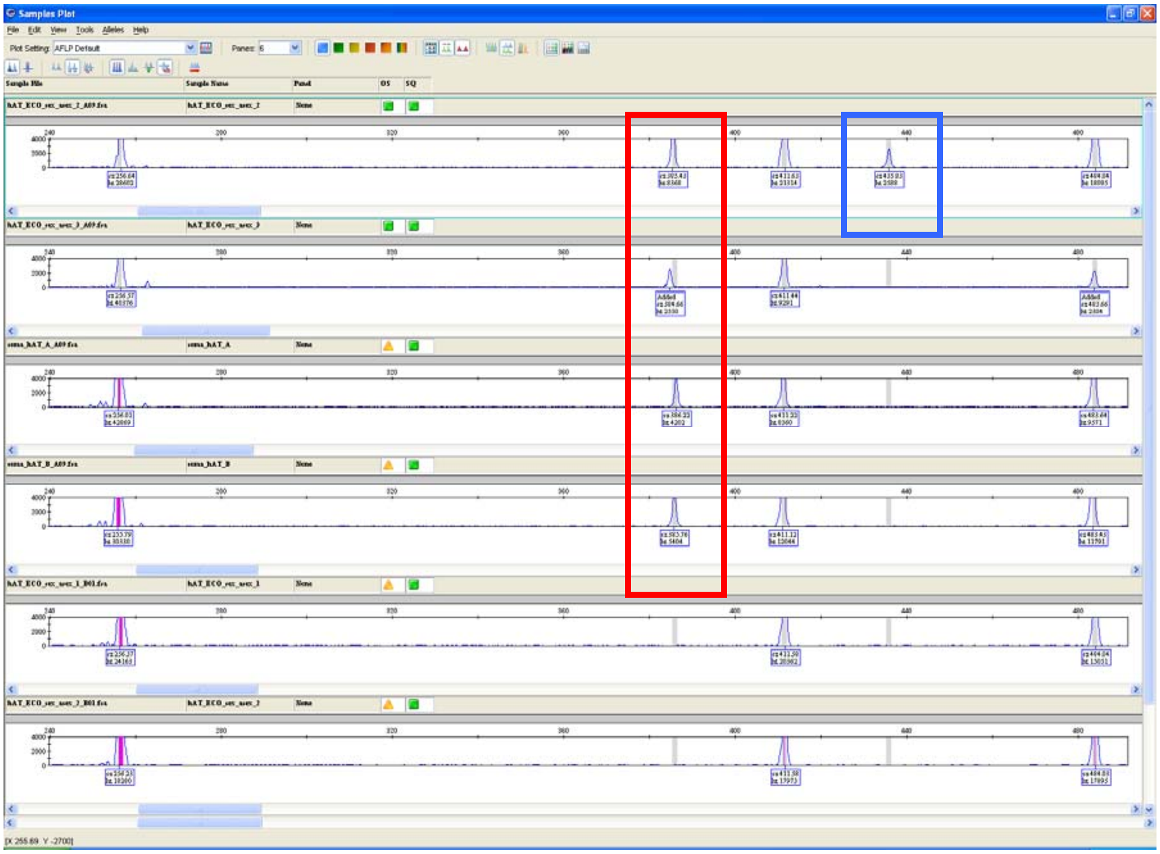

Supplemental Figure 4. Trace file from transposon display reactions showing evidence for a putative germline gain of a copy of the hATA1.1 element. The top four traces show separate runs for the sample indicating a new, replicable peak is found at 385 bp (red box). A putative somatic insertion is also visible in the top trace file (at 436 bp; blue box) where a new peak was observed in only this replicate. The bottom two traces are from another line and represent the ancestral state for the lineages lacking these new copies.
