# Supplementary material for: DNA transposons and the role of recombination in mutation accumulation in Daphnia pulex
Source: Genome Biol. 2010 Apr 30;11(4):R46. doi: 10.1186/gb-2010-11-4-r46 (PMC2884549; doi:10.1186/gb-2010-11-4-r46)
Supplement: Additional file 6 — Peak heights for transposon display performed for hATA1.1 using MA lines of D. pulex. All peaks were above minimum thresholds for inclusion (see Additional file 4). Peaks were scored based on replicability as a: 1, ancestral insertion (common across lineages and replicable within a lineage); 2, new germline insertion (found only in one lineage, replicable in all TD reactions); or 3, putative somatic insertion (unique to one lineage and not replicable in three TD reactions). Peak heights are based on heights scored by Genemapper software for each of three TD replicate reactions performed for each lineage, with the heights for the new germline insertion shown in pink (described in Additional file 5). Lines represent a best fit for each group. [file gb-2010-11-4-r46-S6.pdf]

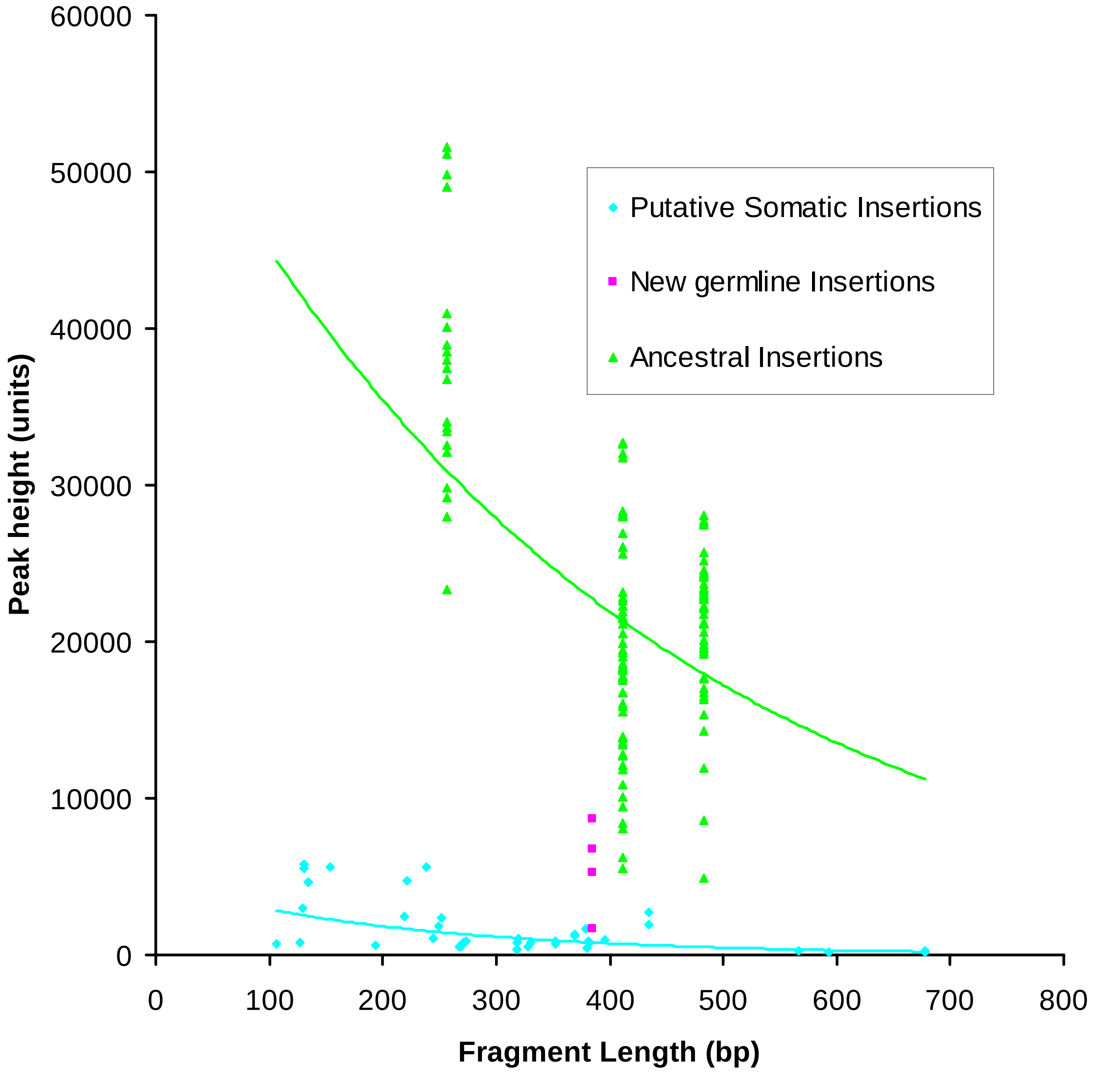

Supplemental Figure 5. Peak heights for transposon display performed for hATA1.1 using MA lines of *D. pulex*. All peaks were above minimum thresholds for inclusion (see Supp. Fig. 3). Peaks were scored based on replicability as a 1) ancestral insertion (common across lineages and replicable within a lineage), 2) new germline insertion (found only in one lineage, replicable in all TD reactions), or 3) putative somatic insertion (unique to one lineage and not replicable in three TD reactions). Peak heights are based on heights scored by Genemapper software for each of three TD reactions performed for each lineage, with the heights for the new germline insertion described in Supplemental Figure 4 shown in pink. Lines represent a best fit for each group.
